# Dissection of artifactual and confounding glial signatures by single-cell sequencing of mouse and human brain

**DOI:** 10.1038/s41593-022-01022-8

**Published:** 2022-03-08

**Authors:** Samuel E. Marsh, Alec J. Walker, Tushar Kamath, Lasse Dissing-Olesen, Timothy R. Hammond, T. Yvanka de Soysa, Adam M. H. Young, Sarah Murphy, Abdulraouf Abdulraouf, Naeem Nadaf, Connor Dufort, Alicia C. Walker, Liliana E. Lucca, Velina Kozareva, Charles Vanderburg, Soyon Hong, Harry Bulstrode, Peter J. Hutchinson, Daniel J. Gaffney, David A. Hafler, Robin J. M. Franklin, Evan Z. Macosko, Beth Stevens

**Affiliations:** 1 F.M. Kirby Neurobiology Center, Boston Children’s Hospital, Boston, MA USA; 2 Harvard Medical School, Boston, MA USA; 3 Stanley Center for Psychiatric Research, Broad Institute of MIT and Harvard, Cambridge, MA USA; 4 Wellcome–Medical Research Council Cambridge Stem Cell Institute, Cambridge Biomedical Campus, University of Cambridge, Cambridge, UK; 5 Department of Neurology and Department of Immunobiology, Yale School of Medicine, New Haven, CT USA; 6 UK Dementia Research Institute, University College London, London, UK; 7 Department of Clinical Neurosciences, University of Cambridge and Cambridge University Hospitals NHS Foundation Trust, Cambridge, UK; 8 Wellcome Sanger Institute, Wellcome Genome Campus, Hinxton, UK; 9 Broad Institute of MIT and Harvard, Cambridge, MA USA; 10 Department of Psychiatry, Massachusetts General Hospital, Boston, MA USA; 11 Howard Hughes Medical Institute, Boston Children’s Hospital, Boston, MA USA

**Keywords:** Neuroimmunology, Microglia, Gene expression analysis

## Abstract

A key aspect of nearly all single-cell sequencing experiments is dissociation of intact tissues into single-cell suspensions. While many protocols have been optimized for optimal cell yield, they have often overlooked the effects that dissociation can have on ex vivo gene expression. Here, we demonstrate that use of enzymatic dissociation on brain tissue induces an aberrant ex vivo gene expression signature, most prominently in microglia, which is prevalent in published literature and can substantially confound downstream analyses. To address this issue, we present a rigorously validated protocol that preserves both in vivo transcriptional profiles and cell-type diversity and yield across tissue types and species. We also identify a similar signature in postmortem human brain single-nucleus RNA-sequencing datasets, and show that this signature is induced in freshly isolated human tissue by exposure to elevated temperatures ex vivo. Together, our results provide a methodological solution for preventing artifactual gene expression changes during fresh tissue digestion and a reference for future deeper analysis of the potential confounding states present in postmortem human samples.

## Abstract (web summary)

Marsh et al. demonstrate that enzymatic dissociation induces an aberrant ex vivo gene expression signature, most prominently in microglia, which when not addressed can substantially confound downstream analyses. They also identify a similar signature in postmortem human brain in snRNA-seq.

## Main

Single-cell and single-nucleus RNA-sequencing (scRNA-seq and snRNA-seq, respectively) has expanded rapidly, as quantified by the dramatic rise in the number of studies and numbers of cells profiled^1,2^. Due to the cost and effort necessary to conduct these assays, considerable resources have been put toward optimizing protocols to isolate high-quality, viable cells. Enzymatic digestion has become the preferred method of isolation from many solid tissues, due to the ability of proteolytic enzymes to easily digest tough tissue substructures^3–9^. Most of these isolation protocols are performed at elevated temperatures to maximize enzyme efficacy and thereby cell yield and viability. However, as the goal of scRNA-seq studies is to produce transcriptional profiles that are reflective of the in vivo state, it is critical to determine exactly how an isolation protocol modifies the transcriptional state of cells ex vivo.

Furthermore, profiling human tissue often requires the use of single-nucleus isolation to enable the profiling of frozen samples from archival tissue banks^10,11^. snRNA-seq is especially critical for tissues such as the brain, where obtaining tissue from living donors is difficult and only possible under very specific disease/injury conditions. However, few studies have examined the human brain for cell-type-specific differences in transcriptional states as a result of tissue handling or acute pre/postmortem processes^12^, and to our knowledge none have done so in a cell-type-specific manner using snRNA-seq.

In this study we sought to understand and characterize the cell-type-specific transcriptional responses to dissociation of mouse brain tissue with standard enzymatic digestion, and to evaluate how acute pre/postmortem variables may affect the transcriptional profiles of nuclei isolated from human brain. In the mouse CNS, we find microglia are preferentially sensitive to ex vivo artifacts and characterize this ex vivo response through in-depth comparative analyses of multiple microglial isolation protocols. We also perform reanalysis of several published datasets to demonstrate how the use of enzymatic and/or room-temperature processing can impact downstream results and conclusions, underscoring its prevalence and the need for new, flexible protocols that address this issue. When enzymatic digestion is experimentally required, we provide an optimized and rigorously validated flexible protocol that utilizes the addition of transcriptional and translational inhibitors during multiple steps of the dissociation process. We demonstrate that this protocol effectively eliminates the artifactual ex vivo transcriptional signature in mouse CNS tissue, as well as in non-CNS cell types and across species.

To examine whether postmortem human brain also displays evidence of similar transcriptional response, we perform snRNA-seq on postmortem brain and reanalyze several published datasets. We find a similar gene signature is present in postmortem microglia and astrocytes, across all snRNA-seq datasets analyzed, although it is highly variable between subjects. Through the use of acutely resected neurosurgical tissue, we reveal that a similar signature can be detected in microglia following prolonged exposure to room temperature. These results suggest that the presence of this signature in postmortem brain samples may be the result of a combination of acute premortem (agonal state, cause of death, comorbidities and so on) and postmortem (postmortem interval (PMI), storage time, RNA quality and so on) variables. Together, our results provide a methodological solution for preventing artifactual gene expression changes during enzymatic digestion of fresh tissue and a reference for future deeper analysis on the potential confounding states present in postmortem human samples.

## Results

### scRNA-seq reveals that mouse microglia are highly sensitive to ex vivo alterations

As the tissue-resident macrophages of the brain, microglia are highly sensitive to perturbations in their environment^13,14^. Previously, we optimized a cold mechanical dissociation protocol to isolate microglia for scRNA-seq with minimal ex vivo transcriptional alterations^15^. However, the yield using this protocol is lower than enzymatic-based protocols, especially in older mice, and therefore it may not be suitable for all experimental designs.

**Fig. 1:**
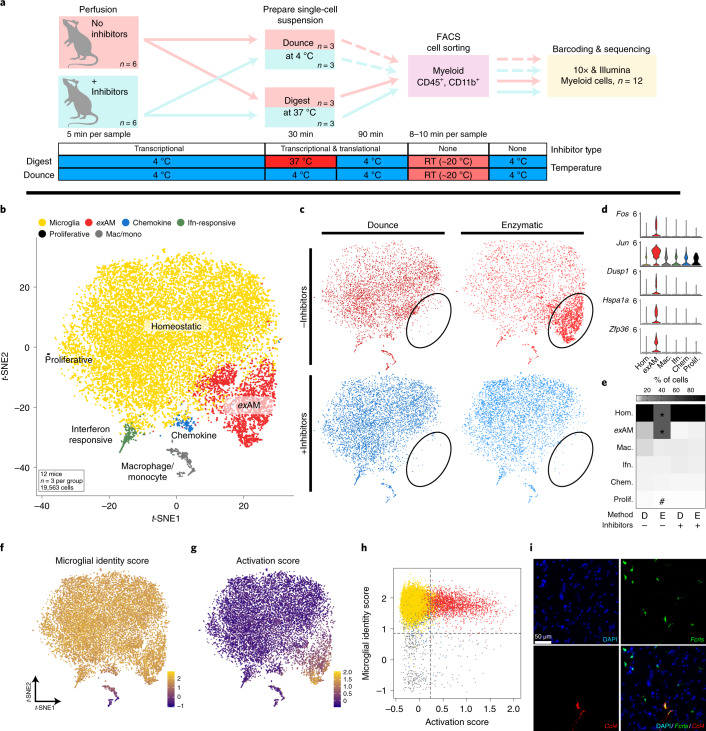
Analysis of sorted microglia confirms profound effect of enzymatic digestion on microglial gene expression via scRNA-seq. **a**, Experimental design schematic for sorted mouse myeloid cells scRNA-seq experiment (Methods and Supplementary Fig. 1a–c). **b**, *t*-SNE plot for the 19,563 cells from *n* = 12 mice (*n* = 3 per group) colored and annotated by cluster (Supplementary Fig. 2). **c**, *t*-SNE plot split by experimental subgroup highlights enrichment of *ex*AM cluster (circled) in ENZ-NONE group. **d,** Gene expression of several exAM cluster markers across each cluster. **e**, Heatmap of the mean percentage of cells in each cluster across conditions (*FDR < 0.005 for ENZ-NONE versus all other groups; ^#^FDR < 0.05 DNC-NONE versus ENZ-INHIB; Benjamin and Hochberg correction for multiple comparisons; (Supplementary Table 3); Methods: differential abundance testing). **f**,**g**, Visualization of gene module scoring results plotted on *t*-SNE coordinates. **f**, Microglial identity score (Methods and Supplementary Table 4). **g**, Activation score based on consensus DEGs from ‘Metacell’ pseudobulk analysis (Methods and Supplementary Tables 4 and 5). **h**, Plot of microglial identity score versus activation score colored by cluster annotation from panel **b**. **i**, smFISH using RNAscope for microglial marker *Fcrls* (green), cluster marker *Ccl4* and counterstained with DAPI; representative images from *n* = 2 independent experiments. Scale bar, 50 μm. Chem, Chemokine; hom, Homeostatic; mac, macrophage; mono, monocyte; prolif, proliferative.

To systematically characterize the microglial response to enzymatic dissociation, we compared our previously optimized cold mechanical dissociation with traditional enzymatic digestion. As we suspected that enzymatic dissociation would induce an aberrant transcriptional response, we modified a protocol previously designed to prevent ex vivo neuronal activity^16^ and added a cocktail of transcriptional or transcriptional and translational inhibitors during multiple steps of the experiment (Fig. 1a). We also maintained tissue/cells on ice with the exception of the enzymatic digestion (37 °C). Microglia/myeloid cells were then sorted as any live dual-positive CD45^+^/CD11b^+^ cells using fluorescence-activated cell sorting (FACS) (Fig. 1a and Supplementary Fig. 1a,b). Of note, we observed substantial loss of some cell-surface receptors following enzymatic digestion, in line with previous work in peripheral immunology (Supplementary Fig. 1d–f and Supplementary Note 1)^3,5–9^. This finding has important implications for any study investigating extracellular proteins, but is especially critical for single-cell techniques such as flow cytometry, cytometry by time of flight (CyTOF) and multimodal sequencing methods (for example, cellular indexing of transcriptomes and epitopes by sequencing (CITE-Seq))^17,18^ (Supplementary Note 1).

Following quality control, we analyzed 19,563 cells from 12 mice (*n* = 3 per condition) (Supplementary Table 1). In addition to a small number of brain-/brain-border-associated macrophages/monocytes, we identified four primary clusters of microglia that were present in all four groups (Fig. 1b). In line with our previous work^15^, we find that adult microglia are largely homogenous. We also identified several smaller clusters of microglia, which we annotated on the basis of the cluster-specific marker genes: proliferative (for example, *Top2a*, *Birc5*), interferon-responsive (*Ifitm3*, *Irf7*) and chemokine-expressing (*Ccl3*, *Ccl4*) (Fig. 1b and Supplementary Fig. 2).

An additional cluster was nearly exclusively composed of cells from samples enzymatically digested without inhibitors (ENZ-NONE) (Fig. 1c,e), characterized by expression of several immediate early genes (IEGs) (for example, *Fos*, *Jun*), stress-induced (*Hspa1a*, *Dusp1*) and immune-signaling genes (*Ccl3*, *Ccl4*) (Fig. 1d and Supplementary Fig. 2c,d). Given the nature of the genes expressed and its overwhelming enrichment in the ENZ-NONE group, we termed this cluster ex vivo ‘activated’ microglia (*ex*AM). We found that ENZ-NONE samples exhibited a significant decrease in the proportion of cells in the homeostatic cluster and a concomitant increase in proportion of cells in the *ex*AM cluster (*false discovery rate (FDR) ≤ 0.004; ENZ-NONE versus all other groups; Methods) (Fig. 1e and Supplementary Table 3). To confirm *ex*AM cells did not simply represent low-quality or dead/dying cells, we analyzed several standard scRNA-seq quality control metrics and demonstrate that the *ex*AM cluster was of equal or significantly better quality compared with either the homeostatic cluster or all non-*ex*AM cells in all of the metrics examined (*P* < 0.05) (Supplementary Fig. 3a).

To identify the differentially expressed genes (DEGs) following enzymatic digestion, we performed differential state analysis using sample-level comparisons^19,20^ by creating pseudobulk ‘Metacells’^15^ by aggregating expression across all cells within each biological replicate and then performing differential expression (DE) analysis using DESeq2 (Supplementary Tables 4–12)^15,19–21^. We also performed marker analysis between clusters using model-based analysis of single-cell transcriptomes (MAST) (Supplementary Tables 13–17 and Methods), but due to issues with subjectivity in clustering^22,23^ and inflation of *P* values in cluster versus cluster comparisons^19,24,25^ we did not use this analysis as the basis for differential state comparisons. We identified a consensus upregulated DE signature shared between the comparisons of each experimental group with the ENZ-NONE group (Supplementary Tables 4–8). This signature was composed of a number of different types of genes including IEGs (for example, *Fos*, *Jun*), genes induced by cellular response to stress (*Hspa1a*, *Dusp1*)^26–28^ and genes associated with regulation of transcription (*Hist1h1d*, *Hist1h2ac*), immune-signaling (*Ccl3*, *Ccl4*) and parts of the NF-κB signaling cascade (*Nfkbiz*, *Nfkbid*) (Supplementary Tables 4 and 5). To visualize which cells in the dataset were enriched for this signature, we performed gene module scoring^29^. We created two scores, one using a core microglial gene signature (Fig. 1f and Supplementary Table 4) aggregated from previous publications and another score, which we refer to as the ‘activation’ score, using the consensus DEG list (Fig. 1g,h and Supplementary Table 4). Analysis of enrichment of this ‘activation’ signature overlaid onto *t*-distributed stochastic neighbor embedding (*t*-SNE) coordinates (Fig. 1g) and against the microglial identity score (Fig. 1h) demonstrated that this activation signature was almost exclusively enriched in cells from the *ex*AM cluster.

The overlap of two cluster markers (*Ccl3*, *Ccl4*) between the chemokine and *ex*AM clusters required additional examination to confirm that the small chemokine population was truly an in vivo state. We performed single-molecule fluorescence in situ hybridization (smFISH) on acutely isolated and snap-frozen tissue, which preserves cells in as close to an vivo state as possible. In line with our previous study and others^15,30^, we confirm that, while extremely rare, the chemokine cluster *Ccl4*^+^ cells are a true in vivo state and not merely an additional dissociation-induced response (Fig. 1i).

To examine whether presence of inhibitors had any adverse impacts on gene expression, we performed further DE comparisons. Analysis of Dounce without inhibitors (DNC-NONE) versus Dounce with inhibitors (DNC-INHIB) groups found no significant DEGs between the groups (Supplementary Tables 9 and 12), indicating that the use of inhibitors in current scRNA-seq pipeline(s) appears to have negligible adverse effect on gene expression.

To further delve into the prevalence of the *ex*AM signature, we generated/analyzed two additional microglial datasets. First, analysis of data from a separate mouse experiment revealed that artifacts are possible even when using ideal isolation methods when there are small differences in sample processing before cell capture (Supplementary Fig. 4 and Supplementary Note 2). We found that an issue resulting in increased time at room temperature for one sample during cell sorting induced the *ex*AM signature selectively in that replicate but not the other three (Supplementary Fig. 4), highlighting the importance of the inclusion of true biological replicates in scRNA-seq experiments. We also confirmed that the *ex*AM signature was not sensitive to differences in single-cell capture/library preparation (Supplementary Figs. 5 and 6).

### Mouse microglia are especially sensitive to ex vivo alterations in gene expression compared with other CNS cell types

**Fig. 2:**
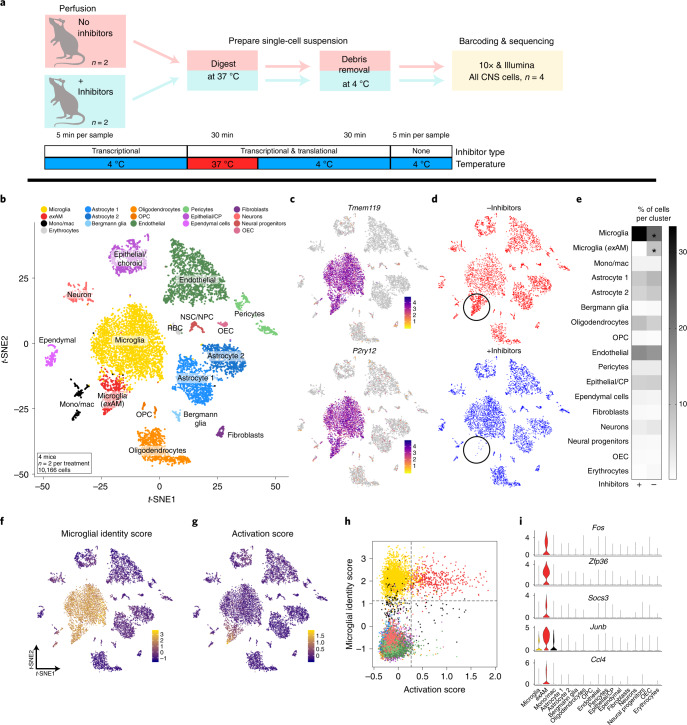
Enzymatic dissociation induces cell-type-specific artifactual gene expression in mice. **a**, Experimental design schematic for scRNA-seq of all CNS cell types (Methods). **b**, *t*-SNE plot of 10,166 cells from 4 mice (*n* = 2 per group), annotated and colored by cell type (Supplementary Fig. 7). **c**, Expression of microglial-specific genes *Tmem119* and *P2ry12* clearly defines two microglial clusters. **d**, *t*-SNE plot split by experimental group (±inhibitor cocktail) highlights lack of *ex*AM cluster in samples digested with inhibitors present (bottom; circled). **e**, Heatmap displaying mean percentage of cells in each cluster across conditions (*FDR < 0.0005; Benjamini and Hochberg correction for multiple comparisons; Supplementary Table 18; Methods: differential abundance testing). **f**,**g**, Gene module scoring results plotted on *t*-SNE coordinates. **f**, Microglial identity score (Methods and Supplementary Table 4). **g**, Activation score based on DEGs from ‘Metacell’ pseudobulk analysis (Methods and Supplementary Table 4). **h**, Scatterplot of gene module scores from **f** and **g** colored by cluster from panel **b**. **i**, Enrichment of genes from activation score in *ex*AM microglia cluster. CP, choroid plexus; NPC, neural progenitor cells; NSC, neural stem cells; OEC, olfactory ensheathing cells; OPC, oligodendrocyte progenitor cells; RBC, red blood cells.

To analyze all CNS cell types simultaneously via scRNA-seq, while maintaining good cell viability, enzymatic dissociation is required. However, few studies have examined whether CNS cell types other than microglia/myeloid cells exhibit altered gene expression when isolated from their in situ environment^31–34^. To characterize the transcriptomic response of all CNS cell types following enzymatic digestion, we used the same transcriptional/translational inhibitor cocktail (Fig. 2a) and performed scRNA-seq on cells without further FACS purification. We analyzed 10,166 cells (post quality control; *n* = 2 per group) and identified 16 broad clusters, representing all of the major cell types found in the CNS (Fig. 2b and Supplementary Fig. 7a,c).

Microglia were the most prominent cell type in the dataset using this digestion protocol (Fig. 2b,c,e), likely indicating that the Miltenyi dissociation system utilized is not appropriate for general unbiased profiling of brain tissue when more accurate cell-type proportions are desired. One of the two microglial clusters was almost completely composed of cells digested without inhibitors (Fig. 2d, circled) and expressed multiple *ex*AM markers (Supplementary Fig. 7c). Analysis of the proportion of cells from each experimental group per cluster revealed that only the microglial clusters differed significantly (*FDR < 0.003) (Fig. 2e and Supplementary Table 18).

DE analysis, using our Metacell pipeline (Methods), revealed a signature of 18 genes that were significantly upregulated and none downregulated when cells were isolated without inhibitors (Supplementary Tables 4 and 19, Fig. 2i and Supplementary Fig. 7c). We also performed marker gene analysis between the two microglial clusters using MAST (Supplementary Table 20 and Methods). The Metacell DEGs exhibited significant overlap with the previously identified DEG list from the microglia-only analysis, confirming that enzymatic digestion, not FACS, was the key factor in the induction of this aberrant response. Further analysis of the *ex*AM signature by gene module scoring and expression of key *ex*AM markers demonstrated that the signature was present almost entirely in cells from the *ex*AM cluster (Fig. 2f–i).

Subclustering analysis of our dataset revealed that microglia/myeloid cells were the only major cell class that exhibited clustering driven by the presence/absence of the inhibitor cocktail (Supplementary Fig. 8a–f). However, in other cell types, including oligodendrocytes/oligodendrocyte precursors, we did identify slightly increased IEG expression in cells digested without inhibitors (Supplementary Fig. 8g–i). These results are in agreement with previous in vitro work which found that microglial reaction to heat stress was significantly faster than for other CNS cell types^35^, although that does not preclude such artifacts being present in other cell types, depending on the details of cell isolation^33^.

### Presence of artifactual microglia signature is common across multiple experimental parameters

To assess the prevalence of this *ex*AM signature beyond the present study, we examined several published datasets^36–40^. This reanalysis included datasets processed with several different scRNA-seq technologies (10X Genomics 3ʹ v.1, 10X 3ʹ v.2, Smart-Seq2, Drop-Seq and Microwell Seq), dissociation enzymes (papain, collagenase), brain regions (whole brain, cortex, subventricular zone (SVZ), spinal cord) and other key experimental variables (Supplementary Fig. 9a–g). We observed enrichment of the *ex*AM signature via gene module scoring in the myeloid populations, consistent with ex vivo activation (Supplementary Fig. 9a–e). We also reanalyzed our previous study^15^, which utilized cold Dounce homogenization, and found minimal presence of cells enriched for the *ex*AM signature (Supplementary Fig. 9f), confirming that cold Dounce homogenization is sufficient to prevent ex vivo activation.

Finally, we sought to characterize the impact of ex vivo activation on the interpretation of results from two common scRNA-seq study types: unbiased characterization of complex tissue (atlas datasets) and case–control studies. First, examination of the cell annotations from two atlas studies that were part of our reanalysis, as well as our own data (Fig. 2a and Supplementary Fig. 9d,e), revealed that ex vivo activation resulted in mis-annotation of *ex*AMs as extra microglial clusters (Supplementary Note 3)^37,39^. Second, reanalysis of two case–control studies^41,42^ demonstrated significant differences in the level of induction of the *ex*AM signature across the conditions being compared (Supplementary Note 4 and Supplementary Fig. 10). Our reanalyses demonstrate that induction of the *ex*AM signature can occur differentially across experimental conditions and may impact downstream analyses.

### Artifactual signature is shared by other immune populations and tissues

We also wondered whether other myeloid lineage CNS cells, such as those found in the choroid plexus, vasculature and pia, were sensitive to ex vivo gene expression changes. Consistent with a previous report in the literature^43^, we found that non-microglial CNS-associated macrophages present in our dataset exhibited enrichment of the artifactual *ex*AM signature when tissue was enzymatically digested without inhibitors (Supplementary Fig. 11a–d).

Our consensus *ex*AM signature does overlap with the dissociation signature in the study by Van Hove et al.[43] (19 of 25 genes in consensus *ex*AM list; Supplementary Tables 4, 5 and 21a). We also examined two other studies in the literature that have utilized scRNA-seq to examine dissociation-induced genes in non-CNS tissue (mouse muscle and kidney)^44,45^. Comparison of our consensus *ex*AM gene list with those studies yielded overlap of 16 and 11 genes, respectively (Supplementary Table 21b,c). When we compare the three lists of overlapping genes with our study, we find the majority of genes are shared across all tissue/cell types, indicative of a common cellular response to dissociation (Supplementary Table 21d). However, we do find some genes that appear tissue-/cell-type-specific to myeloid cells, such as *Ccl3* and *Ccl4* (Supplementary Table 21d).

Given the shared nature of this signature across multiple cell types and tissues, we also wondered whether dissociation artifacts may confound comparisons between circulating immune cells in the blood and analogous tissue-resident populations, which can only be isolated following solid organ/tissue dissociation. We performed reanalysis of two literature datasets that performed this type of comparison in both humans and mice (Supplementary Note 5 and Supplementary Fig. 12)^42,46^. Our results demonstrate that some signatures previously characterized as tissue-resident signatures are often significantly enriched for dissociation-related signatures, underscoring the need for recognizing these artifactual signatures to disentangle them from true in vivo biologically driven transcriptional signatures of tissue residency (Supplementary Note 5 and Supplementary Fig. 12).

To further examine this possibility and broaden the applicability of our inhibitor cocktail beyond the CNS and to other species, we performed a small pilot scRNA-seq analysis of human peripheral blood mononuclear cells (PBMCs). Before PBMC isolation, whole blood was subjected to a mock ‘digestion’ (incubated at 37 °C with or without our transcriptional/translational inhibitor cocktail). PBMCs were then isolated following a standard protocol before scRNA-seq profiling. Analysis of this scRNA-seq dataset revealed significant differences in clustering in most cell types represented in the dataset (including both lymphoid and myeloid populations; Supplementary Fig. 13a,b), dependent on inhibitor condition (Supplementary Fig. 13c, circled).

These differences in clustering were driven by shared gene expression changes across multiple lymphoid and myeloid cell types (Supplementary Fig. 13d–f and Methods). We also applied module scoring using the homologous genes to those in the *ex*AM signature. The mouse signature showed enrichment preferentially in monocytes, again suggesting that some of the signature we originally identified in microglia may be common throughout myeloid lineage cells (Supplementary Fig. 13e). We also found a high concordance between genes that were upregulated in ‘digested’ PBMCs and those that were observed as upregulated in tissue-resident cells following tissue digestion in the Pasciuto et al.^42^ and Crinier et al.^46^ studies (Supplementary Fig. 13f).

### Analysis of human postmortem snRNA-seq identifies similar gene signatures in both microglia and astrocytes

**Fig. 3:**
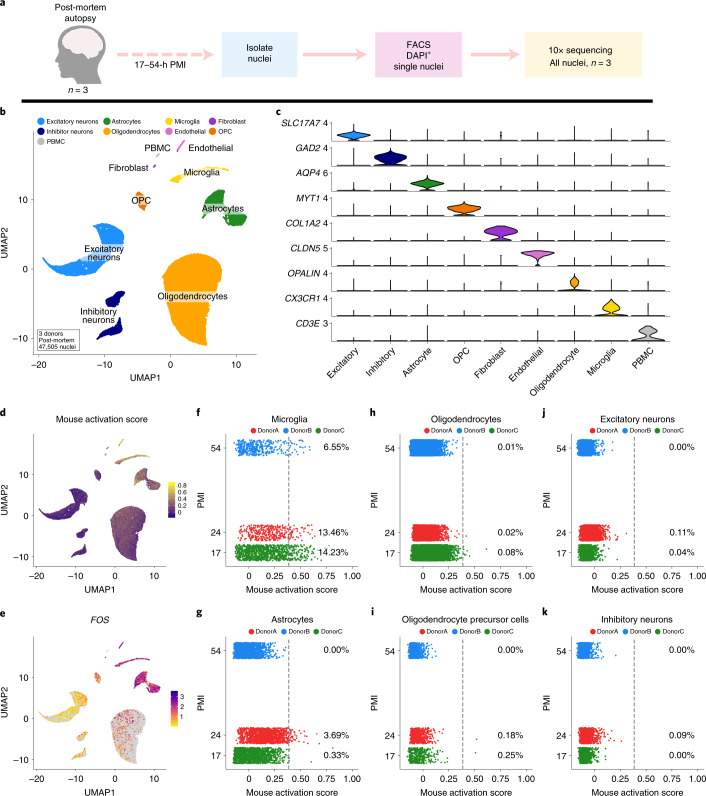
snRNA-seq of human postmortem tissue identifies enrichment of mouse dissociation gene signatures in human microglia and astrocytes. **a**, Experimental design schematic for snRNA-seq of all cell types from frozen postmortem brain tissue. **b**, UMAP plot of 47,505 nuclei analyzed via snRNA-seq from three postmortem subjects following LIGER analysis, colored by major cell type. **c**, Expression of canonical marker genes delineates major cell types. **d**, Visualization of the gene module scoring of the mouse DEG signature on human postmortem snRNA-seq dataset. **e**, Gene expression of *ex*AM signature gene *FOS* across clusters. **f**–**k**, Plots of mouse activation score versus sample PMI (*y* axis) for each of the major CNS cell classes present in the dataset: **f**, microglia, **g**, astrocytes, **h**, oligodendrocytes, **i**, oligodendrocyte precursor cells, **j**, excitatory neurons, **k**, inhibitory neurons. **f-k**, Percentages denote number of nuclei above enrichment threshold denoted with gray dotted line; individual points are colored by donor.

Following our characterization of mouse tissue, we hypothesized that acute pre/postmortem processes might also induce a similar signature in human tissue, despite the lack of need for enzymatic dissociation and/or increased temperatures during nuclei isolation for snRNA-seq. We performed nuclei isolation, sorting and snRNA-seq using the 10X Genomics 3ʹ v.3 kit, from postmortem donors with a wide spread of PMIs (Fig. 3a) (Supplementary Table 2). Integrative analysis using LIGER^47,48^, of 47,505 nuclei across all three donors, identified all of the expected major cell types present in the CNS, as well as some contaminating peripheral immune cells (Fig. 3b,c and Supplementary Fig. 14a–c).

To determine whether the ex vivo dissociation signature we identified in mice was enriched in any particular cell type in human postmortem data, we performed gene module scoring (using homologous genes to the DEG set identified in the analysis of all CNS cell types in the mouse) (Supplementary Tables 4 and 19). Similar to mice, human postmortem microglia exhibited significant enrichment of this signature, as did a small number of astrocytes (Fig. 3d,e). To quantify the enrichment of the signature across the various cell types, we plotted the percentage of nuclei above an enrichment threshold (Methods) versus PMI for each of the donors (Fig. 3f–k). This analysis demonstrated that microglia and some astrocytes exhibited the greatest enrichment of human orthologs of the mouse signature genes and that the percentage of nuclei above threshold per sample was both highly variable and did not correlate with PMI (Fig. 3f–k).

We additionally performed a reanalysis of several published snRNA-seq datasets (Supplementary Table 2)^49–51^ using our LIGER-based pipeline, aggregating a total of 49 samples and 248,026 nuclei, and included both controls (non-neurological disease) and donors with Alzheimer’s disease (Supplementary Fig. 14).

When we performed the same module scoring using the mouse signature, we found that microglia and some astrocytes were again the most enriched for the signature (Supplementary Fig. 14, rightmost column). Due to significant differences in gene/transcript detection sensitivity across these datasets, comparison of the raw expression values remains challenging (Supplementary Fig. 15 and Supplementary Note 6). While these results indicated some enrichment in postmortem tissue, we also wondered if we could identify this signature in postmortem data without a priori knowledge of which genes were a part of the signature. To do this, we performed analysis of the shared LIGER integrative non-negative matrix factorization (iNMF) factors, which have been previously shown to identify gene signatures that correspond to biologically relevant signals^47^. We first extracted major cell types from each dataset and then performed a combined subclustering analysis. In total, we found an iNMF factor in each cell type that shared at least one gene with the combined mouse signature gene lists (Supplementary Table 22). We examined the degree of overlap between the top-ranked genes in each of these shared factors (Methods) and the combined mouse gene lists. Only the factors from the microglial and astrocyte analyses exhibited a 25% or greater overlap with the mouse gene list (Supplementary Table 22).

**Fig. 4:**
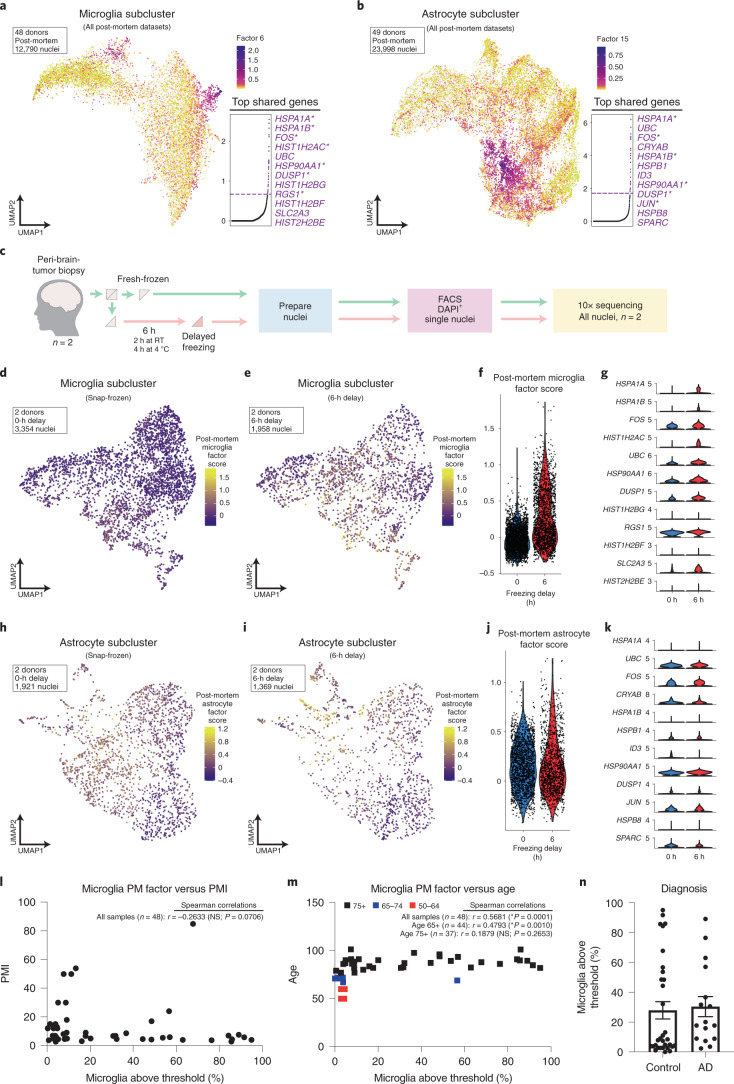
LIGER analysis independently identifies similar gene expression signatures in postmortem data that are enriched in microglia following altered sample processing. **a**, UMAP plot visualizing the enrichment of shared LIGER factor for 12,790 microglial nuclei from 48 samples across all postmortem datasets. **b**, UMAP plot visualizing the enrichment of shared LIGER factor for 23,998 astrocyte nuclei from 49 samples across all postmortem datasets. For both **a** and **b**, inset displays plot of normalized cell-specific factor loading scores across all genes in the dataset (dashed line indicates threshold cutoff for top genes for downstream analysis; Supplementary Table 22). Top loading genes, in order, are shown to the right of the inset plot. **c**, Experimental design schematic for experiment to analyze the effects of altered sample processing on gene expression. **d**–**f**, Visualization of gene module scoring results for score based on postmortem microglia factor, from **a**, in both snap-frozen (**d**) and 6-h delayed freezing (**e**) microglia nuclei on UMAP coordinates or via violin plot split by experimental group (**f**). **g**, Gene expression of top 12 loading genes in microglial factor from **a** split by experimental group (Supplementary Table 23 for DEG results). **h**–**j**, Visualization of gene module scoring results for score based on postmortem astrocyte factor, from **b**, in both snap-frozen (**h**) and 6-h delayed freezing (**i**) astrocyte nuclei on UMAP coordinates or via violin plot split by experimental group (**j**). **k**, Gene expression of top 12 loading genes in astrocyte factor from **b** split by experimental group (Supplementary Table 24 for DEG results). **l**,**m**, Spearman correlation of the percentage of microglia above score threshold for microglia factor score in each postmortem sample versus **l**, PMI and **m**, age of donor; graph annotations list Spearman *r* values and significance. **n**, Plot of percentage of microglia above score threshold for microglia factor score in each postmortem sample split by diagnosis; *n* = 48 independent samples from 5 studies/independent experiments (45 samples are from 4 previously published studies); *n* = 32 control/*n* = 16 Alzheimer’s disease. Data are presented as mean values ± s.e.m. AD, Alzheimer’s disease; NS, not significant; RT, room temperature.

The microglial factor (Fig. 4a) was strikingly similar to the mouse signature, with 79% (30 of 38) of the genes directly overlapping with mouse signature or that were genes from similar families/pathways. The astrocyte postmortem factor (Fig. 4b) exhibited modest overlap with the mouse signature, as 44% (12 of 27) directly overlapped or were genes in similar families. Finally, there were three genes that overlapped between the microglial and astrocyte factors that were not a part of consensus mouse gene lists (*UBC*, *DDIT4* and *HSPB1*), but which unsurprisingly are also part of cellular stress and damage response machinery^52–55^.

### Freezing delay of acutely isolated human tissue is sufficient to induce similar microglial but not astrocyte gene signatures

To further examine potential sources of these iNMF signatures in human microglia/astrocytes, we profiled acutely isolated human brain tissue. Peri-tumor tissue, normally discarded during surgery, was obtained and divided into two fractions. Half of each sample was immediately snap-frozen in liquid nitrogen and the other half was incubated in an artificial cerebrospinal fluid medium for 2 h at room temperature and another 4 h at 4 °C before being snap-frozen (Fig. 4c).

We performed snRNA-seq followed by integrative analysis^47^ to extract the microglia and astrocytes. To determine whether a technical variable (freezing delay) induced a similar signature to that observed in postmortem nuclei, we performed gene module scoring using the LIGER iNMF factor gene lists from postmortem microglia or astrocytes as input (Supplementary Table 22). We found a significant enrichment of the iNMF microglial factor at the 6-h timepoint compared with the 0-h timepoint (*P* < 3.2 × 10^−215^) (Fig. 4d–f), corresponding to 31 of 38 genes from the factor being significantly different (Fig. 4g and Supplementary Table 23). These results suggest that the gene expression signature we identified in postmortem human microglia can be induced by a number of cell-autonomous molecular processes likely including, among others, hypoxia, cell death and temperature stress. In astrocytes, we found a weaker enrichment of the corresponding postmortem signature at the 6-h timepoint (*P* < 0.05) (Fig. 4h–j), wherein only 4 of the 27 genes from the LIGER factor were differentially expressed, and of those only *ATF3* overlapped with the mouse signature (Fig. 4k and Supplementary Table 24), suggestive of differential time scales of activation for these two brain populations, as has been previously described^35^.

### Human postmortem microglial gene signature does not correlate with available metadata variables

We then wanted to determine whether enrichment of this signature correlated with PMI or other available metadata variables. First, we quantified the proportion of microglia or astrocyte nuclei with a gene module score above the enrichment threshold (Methods) per sample in each of the datasets, and then compared that percentage with various metadata variables. The only statistically significant correlation was found with age of the donor (Fig. 4l–n) (Spearman: *r* = 0.3208, *P = 0.0001, *n* = 48). However, this correlation appears to be strongly driven by a small number of samples from younger donors from a single study^50^ (Fig. 4m, red and blue squares). Analysis of correlation in only samples of ages 75+ found no significant correlation (age 75+ Spearman: *r* = 0.1879, *P* = 0.2653, *n* = 37). Similar analyses across the postmortem astrocytes found nearly identical results (Supplementary Fig. 16a–c). Future analyses of a greater number of middle-aged samples will be needed to confirm this correlation and whether age itself is a cause, or rather is simply collinear with other variables such as cause of death or agonal state.

## Discussion

The ability to profile complex tissues at single-cell resolution has the potential to transform our understanding of both normal and disease biology^56–59^, and advances made in multimodal profiling will only serve to enhance insights gained^60,61^. However, these biological discoveries rest on single-cell technologies being able to faithfully measure cell-type-specific expression patterns ex vivo. To obtain reliable measurements of in vivo transcriptional states, it is critical that best practice protocols be established in this emerging field. Previous reports comparing cell isolation protocols or cells postisolation have often focused on aspects such as cell viability and/or yield^4,62^ over whether the isolated cells are truly reflective of their in vivo state^13,14^. We therefore undertook the present study to characterize issues that occur during tissue dissociation or acute pre/postmortem processes at single-cell resolution in the mouse and human brain, to determine the prevalence and impact of the issue in current literature and to identify solutions to aid future studies and analyses.

While our previously optimized cold Dounce homogenization works well for microglia under most conditions, it does have its limitations. Some experimental conditions may dictate the need for greater yield, especially for older or less-sensitive approaches (including many older bulk RNA-sequencing (RNA-seq) studies or protein-based analyses), which is likely why many widely used and widely cited protocols for microglial isolation continue to include enzymatic digestion^4,63,64^. The other major drawback to our mechanical dissociation protocol is that while it purifies microglia, the combination of the tight pestle during the Dounce process and Percoll centrifugation leads to depletion of the majority of other CNS cell types. One recent study proposed a modified mechanical method to isolate all CNS cell types and appeared to succeed in significantly increasing neuronal cell yields during dissociation. However, the study lacked biological replicates needed to perform proper analysis of cell-type abundance and reproducibility^34^. Consequently, the majority of studies have utilized enzymatic dissociation to study all CNS populations at single-cell resolution. However, emerging results from other tissues/cell types have begun to characterize dissociation artifacts following enzymatic digestion^43–45,65,66^. Two previous scRNA-seq studies had examined the brain, but the lack of biological replicates and/or low numbers of cells belonging to rarer cell types, such as microglia, meant that a detailed, reproducible, comparative analysis of the dissociation signature in the CNS has not been performed^32,34^.

Consistent with the reports from other tissues in mice, we found that typical enzymatic digestion of mouse brain tissue induced a host of transcriptional alterations^34,43,44,65^. Interestingly, we found this induction preferentially occurred in microglia and other brain myeloid cells. We also confirm, through reanalysis of the CNS literature, that induction of this aberrant ex vivo response is extremely widespread, and not specific to the enzyme, sequencing technology or specifics of the isolation/tissue processing protocol, and some of the signature appears to be inducible even just by the presence of prolonged room-temperature steps during otherwise favorable mechanical dissociation protocols (Supplementary Notes 2 and 8). In line with previous work on the response of CNS cells to stress^35^, we found that induction of this ex vivo signature occurred only in microglia and other brain-associated myeloid cells, but, while not significant, we found that other cell types may show initial signs of this response, which could become significant under different conditions.

Through the use of a transcriptional and translational inhibitor cocktail at multiple steps of the protocol, we were successfully able to eliminate the ex vivo activation during enzymatic digestion, both in FACS-sorted myeloid cells and in an unsorted preparation of all CNS cell types. Our inhibitor-based protocol offers much greater experimental and tissue-/cell-type flexibility than previously developed experimental and computational solutions for this pervasive issue (see additional discussion of some alternative methods in Supplementary Note 7)^33,34,45,65,67,68^.

Given the prevalence of the signature in current literature, our results have substantial implications for many single-cell datasets: two examples include annotation of cell types and subtypes and DE in case–control studies.

The presence of dissociation-induced changes in atlas or survey studies and experiments analyzing digested versus undigested tissues (or only digested tissue) has the potential to obscure biologically meaningful differences that would otherwise contribute more substantially to clustering and downstream analysis (Supplementary Note 3). Second, in case–control frameworks, we demonstrate with our reanalysis of two examples from the literature that, without knowledge of the presence of the *ex*AM signature, it can result in spurious identification of the *ex*AM signature as differentially expressed (Supplementary Note 4)^41,42,69,70^. A proper baseline that is not confounded by this ex vivo signature is key for the dissection of differences between case and control groups, especially when the biological differences are more subtle (see Supplementary Note 8 for additional discussion of an example). Taken together, our results demonstrate that computational solutions, such as regression of a digestion signature or cell removal, can bias the interpretations of true biological states (Supplementary Notes 4, 5, and 7).

Finally, our results re-emphasize the long-standing importance of validating any RNA-seq results from dissociated or homogenized tissue by orthogonal in situ methods, ranging from ‘classic’ RNA in situ hybridization to new spatial transcriptomics approaches that utilize fresh-frozen, intact tissue sections.

The advent of snRNA-seq has unlocked the ability to unbiasedly assess transcriptional states of the human brain at single-cell resolution^10,11^. However, there are important quality control assessments that need to be performed and considered, especially as regards the impact of acute premortem variables and postmortem processes on gene expression^12,71–77^. Analysis of our own and previous human postmortem snRNA-seq datasets revealed a signature similar to the mouse *ex*AM signature present in both microglia and astrocytes. This human signature was highly variable across donors and was not correlated with PMI or most other available postmortem meta variables. To determine whether this signature could be upregulated in human brain, we designed a study using a technical variable (freezing delay) in acutely resected neurosurgical tissue and demonstrated that microglia upregulated the signature in the timeframe examined.

While signatures in postmortem microglia and astrocytes correlated with age across the entire dataset, the correlation was not robust across post hoc subgroup comparisons. As such, these analyses would substantially benefit from profiling a much greater number of middle-aged samples. Furthermore, based on previous literature, it is also possible that other acute premortem variables such as cause of death, agonal state, increased comorbidities and others may be confounded with age, which will be important for future studies to examine^71–76,78,79^. It is also critical to note that acute premortem factors such as these are not ‘artificial’ as they reflect the true biology associated with agonal state, medication, comorbidities, cause of death and so on, but nonetheless these signatures do confound the ability to perform analysis between desired experimental variables/groups (for example, disease status, cognitive function and so on) when not properly powered/controlled (see Supplementary Note 9 for discussion of results in controls versus patients with Alzheimer’s disease from our current analysis).

The potential for meta variables unrelated to the particular human condition/disease being studied to impact the signatures observed and potentially confound downstream analysis highlights the need for more complete metadata to be made public with studies of postmortem brain. While most studies report very common postmortem factors (for example, age at death, PMI), many variables are often not provided and could have significant impacts, such as RNA integrity number (RIN), which has been shown to confound analyses of dementia-related DEGs^77^. Additionally, very few transcriptomic studies provide information on acute premortem variables or cause of death, which are particularly important as they may potentially have a much larger effect on RNA quality and gene expression^71–76,78–80^. It is worth noting that transcriptional signatures as a result of acute pre/postmortem variables may be far more complex than we have identified here, thus highlighting the need for more detailed metadata to connect transcriptomic signatures to specific covariates. Finally, we suggest that future studies emphasize the sharing of as much metadata as possible, including these pre- and postmortem variables, to enable better more comprehensive analyses. Proper correction and controlling for these variables will be critical not just for understanding what may induce signatures similar to our microglial and astrocyte factors, but for appropriate downstream analysis including detection of DEGs^77^.

Overall, for now, the results of our analysis of human snRNA-seq lead us to caution overinterpretation of biological significance when observing gene signatures similar to the microglial and astrocyte factors we observe in postmortem samples. As a greater number of high-quality human datasets become available, this systematic approach and methods will enable better characterization of this signature and the factors that contribute to it. Most currently available human snRNA-seq datasets are limited by both the proportion of microglia per sample^81^ and the dramatically lower sensitivity of earlier single-cell chemistries/nuclei isolation protocols (Supplementary Note 6). New datasets with optimized protocols, greater numbers of nuclei per patient and greater numbers of patients will be critical to resolving both this potentially confounding state, as well as the in vivo biologically relevant cell states of the human brain^82^. Finally, as we have discussed, it is particularly critical that studies provide substantially more public metadata on both pre- and postmortem variables, both for the interpretation of potentially aberrant gene signatures and also for proper correction and control during analyses focused on understanding the complexities of human biology with scRNA-seq-/snRNA-seq-based methods.

In conclusion, we provide a method by which to eliminate ex vivo transcriptional activation during enzymatic digestion that is easy to implement, is functional across different tissue types and species, and therefore should require minimal effort to incorporate into existing protocols. We also demonstrate how lack of widespread understanding of this signature both in the CNS and periphery has resulted in the pervasiveness of this signature in the current literature and can confound interpretations of results and conclusions of such studies. Using a combination of our own snRNA-seq and a reanalysis of published literature, we find that a similar signature is also expressed by microglia and astrocytes in postmortem brain. We also demonstrate that this signature can be induced by technical variation in sample processing (freezing delay) in human brain tissue, indicating that its presence in postmortem brains may not be reflective of the in vivo variable of interest in a particular study. These datasets and methodologies should inform the design of future scRNA-seq/snRNA-seq experiments to avoid confounding impacts of technical signatures of identification and the interpretation of true biological signals.

## Methods

### Animals

C57BL/6J mice (stock no. 000664) were purchased from Jackson Laboratories. All mouse single-cell sequencing studies were performed with male mice at post-natal day 89-90 (P89-90). Both male and female mice were used for smFISH studies. All animals were group housed on a 12-h/12-h light/dark cycle with access to food and water ad libitum. All experiments were reviewed and overseen by the institutional animal use and care committee at Boston Children’s Hospital in accordance with all National Institutes of Health guidelines for the humane treatment of animals. For a full breakdown of samples and metadata per replicate per dataset for mouse experiments, see Supplementary Table 1. All sample processing was performed in alternating order by experimental group. Sample order and batching are defined in Supplementary Table 1 and the GitHub code repository (link can be found in *Single-cell analysis tools* (below)).

### Acute human tissue

Acutely isolated human brain tissue was obtained with informed consent under protocol REC 16/LO/2168 approved by the National Health Service Health Research Authority. Samples were transferred subject to Material Transfer Agreements between institutions and use and processing of acute brain tissue and sequencing were reviewed and approved by the Boston Children’s Hospital Institutional Review Board and the Broad Institute’s Office of Human Research Subjects Protection.

Adult brain tissue biopsies were taken from the site of neurosurgery resection for the original clinical indication. Samples were dissected into equal volumes and half were immediately (<5 min from time of tissue extraction) snap-frozen in liquid nitrogen and stored at −80 °C. For examination of technical component to gene expression profiles, the remaining half of the samples were placed in Hibernate A low fluorescence supplemented with 1x SOS (Cell Guidance Systems), 2% Glutamax (Life Technologies), 1% P/S (Sigma), 0.1% BSA (Sigma), insulin (4 g ml^−1^, Sigma) and pyruvate (220 g ml^−1^, Gibco) at room temperature for 2 h and then 4 °C for 4 h before being snap-frozen in liquid nitrogen and stored at −80 °C. Samples were shipped on dry ice and stored at −80 °C before processing for nuclei isolation, sorting and sequencing.

All sample processing was performed in random order. Sample order and batching are defined in Supplementary Table 2 and the GitHub code repository.

### Postmortem human brain

Postmortem autopsy tissue from control cases was obtained from the Massachusetts Alzheimer’s Disease Research Center at Massachusetts General Hospital. Tissue was collected with informed consent of patients or their relatives and approval of the Massachusetts General Hospital Institutional Review Board.

Human patient demographic information for tissue in the current study is provided in Supplementary Table 2. Postmortem tissue processing and sequencing experiments were performed at the Broad Institute and approved by the Broad Institute’s Office of Research Subject Protection.

All sample processing was performed in random order. Sample order and batching are defined in Supplementary Table 2 and the GitHub code repository.

### Human PBMCs

Human PBMCs were obtained from blood drawn from a healthy volunteer (male, 35 yr old) in lithium heparin-coated tubes. Blood was collected in accordance with a protocol approved by Yale University Institutional Review Board with the informed consent of the patient. All sample processing was performed in random order. Sample order and batching are defined in Supplementary Table 2 and the GitHub code repository.

### Inhibitor cocktail to prevent activation

Inhibitor stocks were reconstituted and stored as follows. For actinomycin D and anisomycin, stocks were kept for no longer than 1 month following reconstitution, and ideally used within 2 weeks post-reconstitution. Actinomycin D (Sigma-Aldrich, cat. no. A1410) was reconstituted in dimethylsulfoxide at stock concentration of 5 mg ml^−1^ and aliquoted and stored at −20 °C, protected from light. Triptolide (Sigma-Aldrich, cat. no. T3652) in dimethylsulfoxide at stock concentration of 10 mM was aliquoted and stored at −20 °C, protected from light. Anisomycin (Sigma-Aldrich, cat. no. A9789) was reconstituted in dimethylsulfoxide at stock concentration of 10 mg ml^−1^ and aliquoted and stored at +4 °C, protected from light.

Three different buffer solutions were used at different steps in the protocol and are referred to as perfusion buffer, dissection buffer and digestion buffer.

The inhibitor cocktail was added to three different steps of the protocol, as follows:

#### Perfusion buffer (perfusion buffer + inhibitor)

HBSS (without Ca^2+^, Mg^2+^ and Phenol Red; ThermoFisher Scientific, cat. no. 14175-145), actinomycin D with final concentration of 5 μg ml^−1^ (1:1,000 from stock) and triptolide 10 μM (1:1,000 from stock). Add inhibitors immediately before beginning perfusion and keep on ice protected from light. Make fresh for each experiment.

#### Dissection buffer (dissection buffer + inhibitor)

HBSS (without Ca^2+^, Mg^2+^ and Phenol Red), actinomycin D with final concentration of 5 μg ml^−1^ (1:1,000 from stock), triptolide 10 μM (1:1,000 from stock) and anisomycin 27.1 μg ml^−1^ (1:368.5 from stock). Add inhibitors immediately before beginning perfusion and keep on ice protected from light. Make fresh for each experiment.

#### Digestion buffer (digestion buffer + inhibitor)

Digestion buffer/enzyme mix of choice, actinomycin D with final concentration of 5 μg ml^−1^ (1:1,000 from stock), triptolide 10 μM (1:1,000 from stock) and anisomycin 27.1 μg ml^−1^ (1:368.5 from stock). Only add inhibitors to digestion mix immediately before the addition of tissue for digestion.

### Single-cell isolation (all CNS cell types)

Mice were anesthetized and perfused intracardially with ice-cold HBSS with or without inhibitor cocktail in perfusion buffer. Brains were quickly dissected and meninges removed as completely as possible and whole brains were placed in dissection buffer on ice with or without inhibitors and kept covered to protect from light. After all perfusions were completed, brains were quickly placed into a sagittal adult mouse brain slicer matrix (Zivic Instruments, cat. no. BSMAS005-2) and sliced into six even sections.

Immediately following brain slicing, slices were added to Miltenyi gentleMACS C Tubes with or without inhibitor cocktail added to the digestion mixture. Samples were placed in Miltenyi gentleMACS OctoDissociator and the 37C_ABDK_01 protocol was run according to the manufacturer’s instructions. Once the program finished, the samples were briefly spun according to the Miltenyi protocol before being filtered through a 70-μm filter. Samples were washed with HBSS and spun to pellet cells. For isolation of all CNS cells, cell pellets were resuspended and overlaid with an appropriate volume of Miltenyi Debris Removal Solution according to the Miltenyi protocol. Debris was removed from the top layer and the solution was diluted with HBSS and spun to pellet cells. Cells collected following density gradient centrifugation were counted manually via a hemocytometer for loading into the 10X Chromium instrument.

### Single-cell isolation (microglia/myeloid cells)

Enzymatic dissociation of microglia from adult brain was performed identically to all CNS cells as described above until the density gradient centrifugation step.

Mechanical dissociation of microglia from adult brain was performed as previously described^15^, with the addition to ±inhibitor solutions during perfusion, dissection and Dounce steps. Perfusion and dissection were performed identically to the description above. Following dissection, brains were minced with a scalpel and then Dounce homogenized 15–20 times with a loose pestle and then 15–20 times with a tight pestle, all while simultaneously rotating the pestle. The cell suspension was then passed through a pre-wet 70-μm filter.

Following mechanical or enzymatic digestion as described above, cell pellets were resuspended in 40% Percoll (GE Healthcare) in HBSS. Samples were spun for 1 h at 500*g* and 4 °C in a swinging bucket centrifuge. Pelleted cells were washed with HBSS and centrifuged for 5 min at 500*g* and 4 °C and resuspended in 50 μl of FACS buffer (0.5% BSA, 1 mM EDTA, 1× PBS; sterile filtered).

An additional dataset of animals who received a tail-vein injection of PBS 18 h before processing, was processed identically to the mechanical dissociation dataset described above. For the 10X v.3.0 and v.3.1 comparison experiment, dissociation was performed using the cold mechanical Dounce homogenization as described above.

### FACS

All steps were performed on ice or using a prechilled refrigerated centrifuge set to 4 °C, with all buffers/solutions prechilled before addition to samples. Cell suspensions (50 μl) were incubated for 20 min on ice with anti-CD16/CD32 to block Fc receptors (1:50; BioLegend, cat. no. 553141) and with a viability dye, eFlour780 (1:1,000; ThermoFisher Scientific, cat. no. 50-169-66), to identify live cells. The antibody master mix was created by adding all antibodies at 2× their final concentration and 10 μl of Brilliant Stain Buffer Plus (BD Biosciences, cat. no. 566385) to FACS buffer. The master mix was composed of the following antibodies: CD11b-BV421 (BioLegend, cat. no. 101236; clone: M1/70; 1:100 master mix; final staining concentration 1:50), CD45-PE (BioLegend, cat. no. 103106; clone: 30-F11; 1:200 master mix; final staining concentration 1:100), CX3CR1-Alexa Fluor 647 (BioLegend, cat. no. 149004; clone: SA011F11; 1:500 master mix; final staining concentration 1:250). Following Fc block, 50 μl of antibody master mix was added to each sample to achieve 1× antibody concentration. Samples were incubated with staining antibodies for 20 min at 4 °C and then spun down for 5 min at 300*g*, before being resuspended in 500 μl of FACS buffer.

Sterile 96-well plates were precoated with 200 μl of FACS buffer and chilled for 1 h during sample staining. All but 5 μl was removed and plates were kept chilled on ice until samples were ready to sort. To keep samples chilled during as much of the protocol as possible and to prevent contamination, each sample was sorted into a single well of an individual plate. The gating strategy for myeloid cell sorting was as follows (Supplementary Fig. 1b): live (live/dead eFluor780) cells versus debris (forward scatter (FSC) FSC-A versus side scatter (SSC) SSC-A), singlets (FSC-H versus FSC-A), CD45^+^/CD11b^+^. We intentionally set a liberal gate of any CD11b^+^/CD45^+^ cells so as not to bias our scRNA-seq due to changes in cell-surface receptor expression (Supplementary Fig. 1b,c). CX3CR1 was excluded as a parameter used for cell sorting due to differences in staining between isolation methods as the result of cleavage of extracellular epitopes by enzymes (Supplementary Fig. 1d,e). A total of 12,000 myeloid cells were sorted on a special-order BD FACSAria II using a 70-μm nozzle with purity mode, at a total speed of ~10,000 events per second. Each sample took ~5–10 min in total to sort 12,000 live, double-positive, single cells. After sorting, plates were sealed with plastic covers and placed back on ice until proceeding to 10X single-cell capture. For the 10X v.3.0 and v.3.1 comparison experiment, all aspects were the same except 10,000 total events were sorted.

The median fluorescence intensity (MFI) intensity of the CX3CR1 fluorescence (Alexa Fluor 647) of the CD45^+^ and CD11b^+^ population of cells was analyzed using FlowJo-V10, with statistical analysis performed using Prism 8.

During the sorting of the tail-vein PBS-injected dataset, a clog occurred during the sort of the final animal in that dataset. The cells sorted before the clog were discarded and the sample was placed back on ice. Following cleaning of the sorter the sample was sorted a second time into a fresh well. Consequently, the sample was transferred between room temperature and ice twice for a total time at room temperature of approximately 20 min (Supplementary Note 2).

### Single-nucleus isolation and sorting

Nuclei were isolated from human samples according to a similar protocol previously published for use with mouse brain tissue^83^ (https://www.protocols.io/view/frozen-tissue-nuclei-extraction-for-10xv3-snseq-bi62khge). See the protocols.io link for all buffers and solution concentrations. All steps were performed on ice or cold block and all tubes, tips and plates were precooled for >20 min before starting isolation. Briefly, 60-μm sections of cortex (~50 mg) were placed into a single well of a six-well plate and 5–6 ml of extraction buffer was added to each well. Mechanical dissociation was performed through trituration using a P1000 pipette, pipetting 1 ml of solution slowly up and down with a 1-ml Rainin tip (cat. no. 30389212), without creating froth/bubbles, a total of 20 times. Tissue was left to rest in buffer for 2 min and trituration was repeated. A total of 4–5 rounds of trituration and rest were performed (~10 min). The entire volume of the well was then passed twice through a 26-gauge needle into the same well. Following observation of complete tissue dissociation, ~5–6 ml of tissue solution was transferred into a precooled 50-ml Falcon tube. The Falcon tube was filled with wash buffer to make the total volume 30 ml. The 30 ml of tissue solution was then split across two different 50-ml Falcon tubes (~15 ml of solution in each Falcon tube). The tubes were then spun in a precooled swinging bucket centrifuge for 10 min, at 500*g* and 4 °C. Following the spin, the majority of supernatant was discarded (~500 μl remaining with the pellet). Tissue solutions from two Falcon tubes were then pooled into a single tube of ~1,000 μl of concentrated nuclear tissue solution. Approximately 500 μl of wash buffer was then added to bring the total volume of nuclei solution to 1 ml, in an Eppendorf tube. DAPI was then added to the solution at the manufacturer’s (ThermoFisher Scientific, cat. no. 62248) recommended concentration (1:1,000).

Flow sorting of isolated nuclei was performed similarly to the protocols.io protocol (above). Briefly, a 0.2-ml PCR tube was coated with 5% BSA–dissection buffer solution. Solution was then removed and 20 μl of FACS capture buffer was added as a cushion for nuclei during sorting. Nuclei were sorted into a chilled 96-well FACS plate (Sony M800 FACSorter). Sorting was done at a pressure of 6–7, with forward scatter gain of 1% on the DAPI gate. The ‘purity’ mode was used, and no spinning was performed after flow sorting nuclei into PCR tubes. Following sorting, nuclei concentration was counted using a hemocytometer before loading into the 10X Genomics 3ʹ v.3 Chip.

### Human PBMC processing and mock ‘digestion’

Blood was collected in two lithium heparin Vacutainer tubes and the transcription inhibitor cocktail was immediately added to one tube. One-third of the volume in each tube was then incubated with Collagenase IV (2.5 mg ml^−1^) and DNase I (0.2 mg ml^−1^) at 37 °C for 30 min, while the remaining blood was held at 4 °C. PBMCs were then isolated using Lymphoprep gradient centrifugation followed by red blood cell lysis with Ammonium-Chloride-Potassium lysis buffer (ACK). Next, PBMCs were stained with a viability dye (LIVE/DEAD Fixable Red Dead Cell Stain Kit, ThermoFisher, cat. no. L23102) and total live cells (gated on FSC and SSC, followed by doublet exclusion and exclusion of LIVE/DEAD-positive cells) were sorted on a BD FACSAria. Sorted PBMCs were pooled to generate two samples, one derived from the blood treated with inhibitors, one from the control blood, each containing 30% of cells derived from the blood processed at 37 °C and 70% of cells derived from the blood left at 4 °C.

### Mouse single-cell partitioning and library generation

All mouse scRNA-seq experiments, except for the explicit 10X Genomics v.3.0 versus v.3.1 comparison (Supplementary Fig. 5), were performed used 10X Genomics 3ʹ Single-Cell v.2 kits. For the experiments sequencing all CNS cell types, the volume of cell suspension containing 10,000 cells was calculated from manual hemocytometer cell counts and added to an appropriate volume of nuclease-free H_2_O according to the 10X Genomics 3ʹ Single-Cell v.2 user guide. For experiments with sorted myeloid cells, the entire volume of sorted cells (~17–20 μl) was removed from the well and added to the appropriate volume of nuclease-free H_2_O.

Cell suspensions were loaded onto Chromium Single-Cell Chip A (v.2), Chip B (v.3) or Chip G (v.3.1) with other reagents according to the manufacturer’s protocol. Experiments using v.2 and v.3 chips were run using an original Chromium controller and for v.3.1 were run using a NextGEM Chromium controller. Following droplet generation, barcoded single-cell libraries were generated following the manufacturer’s specifications. Library quality control was performed using the Agilent 2100 Bioanalyzer system using the Agilent High Sensitivity DNA quantification kit (cat. no. 5067-4626).

### Human single-nucleus partitioning and library generation

All single-nucleus experiments were performed using 10X Genomics 3ʹ Single-Cell v.3. Following droplet generation, barcoded libraries were generated following the manufacturer’s specifications with one experiment-specific step. For postmortem human samples the cDNA amplification PCR was run for 18 cycles for all samples, acutely isolated human samples were run according to manufacturer’s specifications. Additionally, to ensure sufficient numbers of microglia for downstream analysis, we generated two libraries from each of the postmortem samples.

### Human PBMC partitioning and library generation

Human PBMC single-cell gene expression libraries were prepared and sequenced at the Yale Center for Genome Analysis following standard protocols from 10X Genomics (https://medicine.yale.edu/keck/ycga/sequencing/10x/singcellsequencing/). Single cells were captured using 10X Genomics 5ʹ v.1 kit, targeting 10,000 cells. Following droplet generation, barcoded single-cell libraries were generated following the manufacturer’s specifications.

### Next generation sequencing

All mouse single-cell libraries were sequenced using Illumina NextSeq500, following dilution, pooling and denature/dilution according to the Illumina denature and dilution guidelines for NextSeq500 High Output flow cells. All 16 mouse libraries (each representing an individual animal for both all CNS cells and sorted myeloid cells) were sequenced as follows, according to 10X Genomics instructions. For 3ʹ Single-Cell v.2 kits: Read 1: 26 base pairs (bp) (16-bp cell barcode, 10-bp unique molecular identifier (UMI)); Index 1: 8 bp (Illumina i7 sample index); Read 2: 98 bp (transcript insert).

The libraries from the tail-vein PBS-injected dataset were submitted to Harvard Medical School’s BioPolymers sequencing core for quantification, pooling and sequencing. Samples were quality controlled via Agilent Tapestation and quantitative PCR by the Biopolymers Core before running on an Illumina NextSeq500/550 High Output v.2.5 150-cycle flow cell as follows, according to the 10X Genomics instructions for 3ʹ Single-Cell v.2 kits: Read 1: 26 bp (16-bp cell barcode, 10-bp UMI); Index 1: 8 bp (Illumina i7 sample index); Read 2: 98 bp (transcript insert).

For the 10X v.3.0 versus v.3.1 comparison experiment, the library pool containing all six samples was loaded onto an Illumina NextSeq500/550 High Output v.2.5 150-cycle flow cell and sequenced as follows, according to the 10X Genomics instructions for 3ʹ Single-Cell v.3.0/v.3.1 kits: Read 1: 28 bp (16-bp cell barcode, 12-bp UMI); Index 1: 8 bp (Illumina sample index); Read 2: 91 bp (transcript insert).

Human single-nucleus libraries underwent two different sequencing procedures. First, libraries were pooled to equimolar DNA concentrations and sequenced at low depth (~5,000–10,000 reads per nucleus). For this run, snRNA-seq libraries from both acute and postmortem samples were pooled, diluted and denatured according to Illumina specifications and loaded on an Illumina NextSeq500/550 High Output v.2.5 150-cycle flow cell and sequenced as follows, according to the 10X Genomics specifications for 3ʹ Single-Cell v.3 kits: Read 1: 28 bp (16-bp cell barcode, 12-bp UMI); Index 1: 8 bp (Illumina i7 sample index); Read 2: 91 bp (transcript insert). The approximate number of nuclei recovered per sample was determined via the output of the Cell Ranger ‘count’ pipeline (below). A new library pool was then created from the original libraries, accounting for differences in nuclei number per sample to achieve equal read depth per nucleus across samples. The new library pool was then sequenced on a NovaSeq 6000 using an S2 100-cycle flow cell and using slightly modified read parameters (Read 1: 28 bp; Read 2: 89 bp; i7: 8 bp). All NovaSeq 6000 sequencing was performed by the Broad Institute’s Genomics Platform. Only the results of sequencing via NovaSeq 6000 were used for analysis.

Human PBMC libraries were sequenced on a NovaSeq 6000 using an S4 200-cycle flow cell and using the 10X recommended read parameters for 5ʹ v.1 gene expression: Read 1: 26 bp; Read 2: 91 bp; i7: 8 bp. Human PBMC library sequencing was performed by the Yale Center for Genome Analysis.

### Single-cell/nucleus data preprocessing

All preprocessing of sequencing data (except for the human PBMC experiment) was performed on Harvard Medical School’s O2 High Performance Compute Cluster. Human PBMC data were processed on the Yale University Center for Research Computing High Performance Compute Cluster.

#### Mouse data preprocessing

Raw Illumina bcl files for the all CNS cells and sorted myeloid cells datasets were demultiplexed using Cell Ranger v.3.0.0 and bcl2fastq v.2.20.0.422 using the ‘mkfastq’ step with default specifications. Individual sample gene expression matrices were generated using the Cell Ranger v.3.0.0 ‘count’ step using the default mm10 genome supplied by 10X Genomics Cell Ranger 3.0.0 (reference annotation corresponds to the filtered version of Ensembl v.93; see the 10X support website for further information (https://support.10xgenomics.com/single-cell-gene-expression/software/release-notes/build)). Sample-specific results were then aggregated into a combined output matrix using the Cell Ranger v.3.0.0 ‘aggr’ function, specified with ‘normalize = none’ so that all reads for all samples were included in downstream analysis. Cell Ranger ‘aggr’ was performed individually for all CNS cell samples (*n* = 4) and FACS-sorted microglia samples (*n* = 12).

The PBS tail-vein-injected dataset was processed using Cell Ranger v.2.2.0 and bcl2fastq v.2.20.0.422 using the ‘mkfastq’ step with default specifications. Individual sample gene expression matrices were generated using the Cell Ranger v.2.2.0 ‘count’ step using the default mm10 genome supplied by 10X Genomics Cell Ranger v.2.2.0 (reference annotation corresponds to the filtered version of Ensembl v.84; see the 10X support website for further information (https://support.10xgenomics.com/single-cell-gene-expression/software/release-notes/build)). Sample-specific results were then aggregated into a combined output matrix using the Cell Ranger v.2.2.0 ‘aggr’ function, specified with ‘normalize = none’ so that all reads for all samples were included in downstream analysis.

For the 10X version comparison, the dataset was processed using Cell Ranger v.5.0.0 and bcl2fastq v.2.20.0.422 using the ‘mkfastq’ step with default specifications. Individual sample gene expression matrices were generated using the Cell Ranger v.5.0.0 ‘count’ step using the default mm10 genome supplied by 10X Genomics Cell Ranger v.5.0.0 (reference annotation corresponds to the filtered version of Ensembl v.98; see the 10X support website for further information (https://support.10xgenomics.com/single-cell-gene-expression/software/release-notes/build)).

#### Human data preprocessing

For snRNA-seq of brain samples, raw Illumina bcl files were demultiplexed using Cell Ranger v.3.1.0 and bcl2fastq v.2.20.0.422 using the ‘mkfastq’ step with default specifications. A custom premRNA reference genome was generated using instructions from 10X Genomics. The default 10X GRCh38 genome (reference annotation corresponds to the filtered version of Ensembl v.93) was used for modification. Individual sample gene expression matrices were generated using the Cell Ranger v.3.1.0 ‘count’ step using this custom GRCh38 genome. Sample-specific results were then aggregated into a combined output matrix using the Cell Ranger v.3.1.0 ‘aggr’ function, specified with ‘normalize = none’ so that all reads for all samples were included in downstream analysis. Cell Ranger ‘aggr’ was performed once for all human samples (*n* = 10).

The human PBMC dataset was processed using Cell Ranger v.3.1.0 and bcl2fastq v.2.20 using the ‘mkfastq’ step with default specifications. Individual sample gene expression matrices were generated using the Cell Ranger v.3.1.0 ‘count’ step using the default GRCh38-3.0.0 genome supplied by 10X Genomics Cell Ranger v.3.1.0 (corresponds to filtered version of Ensembl v.98; see the 10X support website for further information (https://support.10xgenomics.com/single-cell-gene-expression/software/release-notes/build)).

### Single-cell analysis tools

Single-cell analysis was performed using R (v.3.4.3, v.3.5.1 and v.3.6.1) (https://www.r-project.org), and primarily using the single-cell analysis package Seurat (primarily v.2.3.4, v.3.1.5 and v.3.2.3)^84,85^. Additional analysis of human single-nucleus samples was performed using the LIGER development branch ‘online’^47,48^. Additional scRNA-seq analysis and plotting were performed using scCustomize v.0.5.0 (https://samuel-marsh.github.io/scCustomize/; ref. ^86^). Other supplemental R packages were used as described in the methods below.

Code required to reproduce Seurat or LIGER objects used for analyses and plotting can be found at: https://github.com/samuel-marsh/Marsh_et-al_2022_scRNAseq_Dissociation_Artifacts. Questions or correspondence regarding analysis/code can be directed to samuel.marsh@childrens.harvard.edu.

### Mouse scRNA-seq analyses

Similar basic analysis pipelines were used in many of the analyses. Full details to replicate the analysis pipelines described briefly below can be found in code scripts available on GitHub (https://github.com/samuel-marsh/Marsh_et-al_2022_scRNAseq_Dissociation_Artifacts).

### Initial quality control and clustering (mouse datasets)

In brief, cells were filtered using dataset-specific parameters on the basis of genes per cell, UMIs per cell and percentage of mitochondrial gene reads per cell. Data were log-normalized and highly variable genes were selected using mean expression and dispersion cutoffs. Data were scaled and UMIs per cell and mitochondrial gene percentage per cell were regressed out. Following prinicpal component analysis (PCA), relevant principal component (PC) cutoffs were selected for downstream analyses based on a combination of JackStraw analysis, ElbowPlot of PC variance and manual examination of PCs. Clustering was then performed, first by creation of a shared nearest neighbor (SNN) graph using PCs previously selected, followed by Louvain clustering with a dataset-specific resolution parameter. The resolution parameter of Louvain clustering was iteratively performed to settle on an appropriate final resolution. Dimensionality reduction was then performed using *t*-SNE. Datasets were additionally quality controlled at this step by checking for and removing doublets on the basis of dual cell-type marker gene expression. Following removal of doublets, the analysis pipeline was re-run using the filtered dataset and slightly altered parameters.

### Cluster annotation (mouse)

Cluster annotation was performed through manual analysis of the output of differentially expressed marker genes from the output of the Wilcoxon rank sum test run via Seurat. Results were filtered in two different but complementary ways to identify marker genes, sorting for top genes by log fold-change or by calculating a difference metric for percentage of cells expressing versus not expressing in a given cluster and then sorting on top differences. For the all CNS cells dataset, marker genes were then used for annotation of cell type/subtype using previous single-cell studies^15,33,37,87,88^. For the myeloid/microglia dataset, clusters were annotated with names reflecting the genes enriched in that particular cluster (for example, interferon-responsive, chemokine and so on) or on the basis of known biology (for example, proliferative).

### Differential abundance analysis (mouse)

To determine if the proportions of cells per cluster were significantly different between the different protocols, the clusters were compared using the R package speckle (https://github.com/Oshlack/speckle). The Seurat objects for the all CNS analysis and sorted microglia were used as inputs for the analysis. The design matrix for the all CNS analysis simply included the two experimental groups as comparison (inhibitor versus no inhibitor). For the sorted microglia analysis, in addition to the four experimental groups, the design matrix also included batch as a variable. One of the results of this analysis does require significantly more cells to confirm as, while the proliferative cluster was statistically significant between the DNC-NONE and enzymatic digestion with inhibitors (ENZ-INHIB) groups (^#^FDR ≤ 0.02) (Fig. 1e and Supplementary Table 3), this increase should be interpreted with caution, given that this population makes up only 0.1% of total cells (27 of 19,563) across all 12 samples.

### Subclustering (mouse)

Subclustering analysis of the all CNS cell types mouse dataset was performed by first calculating the number of cells per replicate for each of the clusters. Clusters were then combined into major cell classes by combining related/highly similar cell types. To enhance confidence in subclustering analysis, only cell classes with greater than 80 cells per replicate (>300 cells total) were considered for subclustering. This criterion led to six cell classes for subclustering: (1) myeloid (microglia, *ex*AM microglia, monocytes/macrophages); (2) astrocytes (astrocyte 1, astrocyte 2, Bergmann glia); (3) endothelial/pericytes (endothelial cells, pericytes); (4) oligodendrocytes (oligodendrocytes and oligodendrocyte precursor cells); (5) epithelial (epithelial cells/choroid plexus); (6) neurons (neurons and neural progenitor cells). Each of the classes was subsetted from the original dataset and reanalyzed using a similar pipeline to the original analysis as described above.

### Metacell DE analysis (mouse)

To perform sample-level DE analyses, we utilized the Metacell analysis as previously described^15^. Each ‘meta-cell’ is a pseudobulk replicate created by aggregating expression from all cells within a biological replicate, and pseudobulk profiles can then be analyzed via traditional bulk RNA-seq analysis pipelines. A recent comparative analysis found that pseudobulk analysis methods are among the best-performing methods for differential state analysis and for making sample-level comparisons from single-cell data, and help to overcome some of the inherent sparsity in scRNA-seq data^19,89^. This pseudobulk approach also overcomes the inherent limitations of DE analysis comparisons between clusters, which suffer from using the same data to both select and test the null hypothesis^24,25^. For our pipeline, sample normalization and DE analysis were performed using DESeq2 (ref. ^21^) using a simple pipeline. Genes were defined as differentially expressed with adjusted *P* value <0.05 and log_2_ fold-change less than −0.58 or greater than 0.58 (corresponding to 1.5-fold-change).

### Cluster comparison analysis

To perform analysis of DEGs across clusters of microglia we used MAST^90^. The MAST framework uses a two-part generalized linear model to test for DE while controlling for specified covariates. In our analysis of mouse microglial isolation protocols (Fig. 1), we specified log(number of UMIs), percentage of reads mapping to mitochondrial genes per cell, batch and Method_x_Inhibitor (four-group variable containing method plus inhibitor status; for example, ENZ-NONE) as fixed effects (Supplementary Tables 13–17). In our analysis of the two microglial clusters in our all CNS cell types analysis (Fig. 2), we used log(number of UMIs), percentage of reads mapping to mitochondrial genes per cell and presence of inhibitors as fixed effects (Supplementary Table 20). Due to the issues mentioned above with statistical analysis performed between clusters using the same data used to generate clustering^24,25^, we did not employ any statistical cutoff or use these lists in any downstream module scoring or analysis. In the output tables the coefficient is the discrete component of MAST and the *P* value is from the combined hurdle model.

### Gene module scores (mouse)

Creation of microglial identity scores and gene module scores was executed in Seurat based on a previously published technique^29^. Microglial identity score was based on well-established canonical microglial markers (Supplementary Table 3). ‘Activation’ score was based on results of the Metacell pseudobulk DE analysis performed using DESeq2 (Supplementary Tables 4–8). For the microglia/myeloid dataset, the consensus DE signature was identified by taking the intersection of the three pair-wise comparisons: DNC-NONE versus ENZ-NONE, DNC-INHIB versus ENZ-NONE, ENZ-INHIB versus ENZ-NONE.

### Reanalysis of publicly available datasets (mouse myeloid/microglia)

Raw count matrices or loom files containing count matrices were obtained from the National Center for Biotechnology Information (NCBI) GEO database, laboratory websites or directly from authors, as specified in Supplementary Fig. 9g, Supplementary Tables 1 and 2, and the GitHub ReadME information table (see the link above to GitHub code repository). For the datasets in Supplementary Fig. 9 that were composed of different genotypes or treatments, only cells from WT or control mice were included in downstream analysis.

Datasets were processed with a similar basic Seurat pipeline with minor dataset-specific changes (for full details and a breakdown of the analyses performed, see code in the GitHub Repository). Briefly, each dataset was filtered on dataset-specific parameters for genes per cell, UMIs per cell and percentage of mitochondrial genes. Data were log-normalized and variable features were selected using ‘mean.var.plot’ or ‘vst’ methods using dataset-specific thresholds and then scaled and centered. Examination of PC loadings and results of JackStraw analysis were used to determine significant PCs for downstream clustering and visualization. Clustering was performed using the Louvain algorithm using dataset-specific resolution parameters. Clustering results were visualized using *t*-SNE or Uniform Manifold Approximation and Projection (UMAP). Activation and microglial scores were added as described above using the microglial Metacell consensus DEG list.

For datasets containing more than just microglia/myeloid cells, the analysis pipeline was first run to identify myeloid cells. Clusters containing myeloid cells were isolated and the entire pipeline run again to analyze myeloid cells using new parameters. All datasets were then further subclustered to manually examine and remove any remaining doublets.

For datasets in Supplementary Figs. 8 and 10, multiple genotypes, tissues and samples were used as specified in the Results section/code. For analysis of the results found by Keren-Shaul et al.^41^, Crinier et al.^42^ and Pasciuto et al.^46^, *ex*AM module score enrichment statistical testing was performed using Wilcoxon rank sum test, in base R, comparing between groups specified in the Results section/figures.

### Human snRNA-seq/scRNA-seq analysis

Similar basic analysis pipelines were used in many of the analyses. Full details to replicate the analysis pipelines described below can be found in code scripts available on GitHub (https://github.com/samuel-marsh/Marsh_et-al_2022_scRNAseq_Dissociation_Artifacts).

### Initial quality control filtering (human datasets)

In brief, data were imported using Seurat v.3 and nuclei datasets were filtered using dataset-specific parameters on the basis of genes per nucleus, UMIs per nucleus and percentage of mitochondrial gene reads per nucleus. Datasets were then converted to LIGER objects, with each sample serving as a separate ‘dataset’ in LIGER.

### Doublet/low-quality nuclei filtering (human datasets)

To detect and filter doublets and/or low-quality cells in snRNA-seq datasets, we performed iterative rounds of clustering using LIGER. Most analyses utilized the new online iNMF algorithm^48^, although some utilized traditional iNMF, when low cell numbers per sample were present. Following iNMF and quantile normalization, cells were clustered using Louvain clustering, followed by UMAP dimensionality reduction. Clusters were then annotated with one of the following broad cell class labels: excitatory neuron, inhibitory neuron, oligodendrocyte, oligodendrocyte progenitor cell, astrocyte, microglia, endothelial, fibroblast, pericyte, immune/PBMC (peripheral immune cells) or doublets. Annotation of broad cell classes and subtypes was performed using ref. ^91^ and with canonical marker genes as a reference. Each broad cell class was then subsetted and reanalyzed and clustered again. Doublet identification was then performed using a combination of marker gene expression and shared LIGER ‘factors’ to identify nuclei that expressed markers or combinations of markers that are exclusive to two different cell classes. These subclusters were then classified as doublets and all barcodes corresponding to nuclei is those clusters were removed from the analysis. Some datasets required additional rounds of analysis and subclustering to completely remove likely doublet nuclei that were not found during the first round.

### Clustering and LIGER factor analysis

Following doublet removal, the ‘cleaned’ subclusters for each major cell class were merged and full analysis and clustering was performed using LIGER. After cleaning, LIGER factors for each major cell class were examined by plotting the factor on UMAP plots and examining the top genes that loaded on each factor. Factors with genes indicative of activation, stress response or similarity to mouse signature were selected for further analysis. For each selected factor we plotted the normalized cell-specific factor loadings for each gene and selected cutoff thresholds (Fig. 4a,b, dashed lines). The list of genes above the cutoff in each factor (Supplementary Table 22) was compared with the union of the two mouse DEG activation lists (Supplementary Table 4) to analyze overlap and similarities.

### Gene module score thresholding (human postmortem)

To quantify the enrichment of the microglia and astrocyte postmortem LIGER factors across all samples in each dataset, we performed gene module scoring using Seurat, as described above. To determine whether a particular cell’s score was defined as ‘enriched’, we first determined dataset-specific thresholds.

To determine thresholds for enrichment we first created an intersect gene list of all genes present across all five datasets. We then created 1,000 random gene lists of equivalent length to the microglial LIGER factor (38 genes) and the astrocyte LIGER factor (27 genes). We then downsampled each dataset so that all of the major cell classes had the same number of cells, so that cell number/proportion did not influence outlier detection. We then performed gene module scoring using the random lists. To determine a cutoff for microglia we calculated an outlier threshold for each of the random scores. The outlier threshold was calculated as median score (across all cell types) + 3 × median absolute deviation of score (across all cell types). We then plotted all of the outlier thresholds for each of the 1,000 random scores. A cutoff was selected at the top 2.5% of outlier thresholds. We then determined the proportion of microglia per sample that exhibited a postmortem LIGER microglia factor score above that cutoff. This process was repeated for astrocytes using the random gene lists of equivalent length to the astrocyte postmortem LIGER factor. These enrichment proportions per sample were then used for correlational analysis to metadata variables (PMI, age and so on) (Fig. 4l–n and Supplementary Fig. 16a–c).

### Time delay freezing analysis

Nuclei from acutely resected human tissue were analyzed using a similar pipeline to previously described for human tissue analysis. Following final clustering, the microglial and astrocyte clusters were subsetted and converted to Seurat class for analysis comparing with postmortem LIGER factors. Module scoring was performed using the LIGER factor genes (Supplementary Table 22) as the input for postmortem microglial and astrocyte scores on the microglial and astrocyte subclusters, respectively. DEG analysis was performed using Wilcoxon rank sum test with Bonferroni correction, comparing between the identity classes of 0 h versus 6 h as implemented in the FindMarkers function of the Seurat package. Analysis of microglia and astrocyte LIGER factor module score enrichment testing was performed using Wilcoxon rank sum test, comparing between 0-h and 6-h cells.

### Human PBMC mock ‘digestion’ analysis

Human PBMCs from the mock ‘digestion’ with and without inhibitors were analyzed using a similar Seurat pipeline to those used in the mouse scRNA-seq analyses described above (see full code on GitHub). Following initial analysis, clusters were then annotated with one broad cell class label using canonical marker genes from Immgen and the Human Cell Atlas: Immune System Atlas^56,92^. Each broad cell class was then subsetted and reanalyzed and clustered again. Doublet identification was then performed using combinations of marker gene expression to identify nuclei that expressed markers or combinations of markers that are exclusive to two different cell classes. ‘Cleaned’ subclusters were then merged and reanalyzed. Final cluster annotation was performed using a combination of manual marker gene analysis (as above) and automated reference-based mapping via Seurat/Azimuth^93^. DEG analysis was performed in major cell classes (T cells, B cells and so on) with greater than 100 total cells in the final analysis. DEG analysis was performed using Wilcoxon rank sum test with Bonferroni correction, comparing between the identity classes of 0 h versus 6 h as implemented in the FindMarkers function of the Seurat package.

### RNAscope smFISH

Mice were anesthetized and perfused intracardially with ice-cold HBSS. Brains were immediately dissected and flash-frozen using the vapor phase of liquid nitrogen. Following freezing, brains were embedded in OCT (Tissue-Tek) and frozen on dry ice before storage at −80 °C before sectioning. OCT-embedded samples were mounted on a cryostat and cut into 16-μm sagittal sections. Slides were kept frozen at all times during sectioning and then moved to −80 °C for storage before RNAscope.

RNAscope Fluorescent Multiplex Assay (ACD Biosystems) was performed according to the manufacturer’s protocol for fresh-frozen tissue. Brain sections were hybridized with three mRNA probes per experiment. The following genes/probes were used: *Fcrls* (microglia/myeloid), *Ccl4*. The probes were amplified according to the manufacturer’s instructions and labeled with the following fluorophores for each experiment: Alexa 488 nm, Atto 550 nm, Atto 647 nm. High-resolution images were taken using ×60 magnification on a Zeiss LSM confocal microscope.

### Statistics

Statistical analysis of data was performed mainly using the R packages base R, Seurat, DESeq2 or speckle, with some additional analyses in Prism 8 (GraphPad Software). No explicit statistical methods were used to predetermine sample sizes but our sample sizes exceed those reported in previous publications^32,34,43^.

The experiments used a mixture of blinded and nonblinded analyses, as follows. Experimental collection and processing of samples were not performed blind due to the conditions of the experiment. scRNA-seq/snRNA-seq were not performed blind but using automated and appropriate field standard analysis techniques that require metadata to be present. smFISH experiments and imaging were performed blind.

The only datapoints that were excluded from this analysis were those cells defined as low-quality (see above scRNA-seq/snRNA-seq analyses sections and GitHub code repository) or those that were defined as doublets using manual annotation of doublet cells by the experimenter (see above scRNA-seq/snRNA-seq analyses sections and GitHub code repository). All filtering was performed using field standard techniques as described in the analysis subsections above. All of these datapoints are present in raw data deposited in NCBI GEO, the Database of Genotypes and Phenotypes (dbGaP) and the European Phenome-Genome Archive (EGA). Code to recreate all filtering performed can be found at the GitHub repository: https://github.com/samuel-marsh/Marsh_et-al_2022_scRNAseq_Dissociation_Artifacts

### Reporting Summary

Further information on research design is available in the Nature Research Reporting Summary linked to this article.

## Online content

Any methods, additional references, Nature Research reporting summaries, source data, extended data, supplementary information, acknowledgements, peer review information; details of author contributions and competing interests; and statements of data and code availability are available at 10.1038/s41593-022-01022-8.

## Supplementary information

Supplementary Information Supplementary Information Table of contents, Supplementary Figs. 1–16, Notes 1–9 and references.

Reporting Summary

Supplementary Table 1 Sample information (mouse experiments).

Supplementary Table 2 Sample information (human experiments).

Supplementary Table 3 Differential abundance (mouse microglia).

Supplementary Table 4 Gene module scoring gene lists.

Supplementary Table 5 Microglia meta cell individual comparisons summary.

Supplementary Table 6 DESeq2 sig. genes results DNC-NONE versus ENZ-NONE.

Supplementary Table 7 DESeq2 sig. genes results DNC-INHIB versus ENZ-NONE.

Supplementary Table 8 DESeq2 sig. genes results ENZ-INHIB versus ENZ-NONE.

Supplementary Table 9 DESeq2 sig. genes results DNC-NONE versus DNC-INHIB.

Supplementary Table 10 DESeq2 sig. genes results DNC-NONE versus ENZ-INHIB.

Supplementary Table 11 DESeq2 sig. genes results DNC-INHIB versus ENZ-INHIB.

Supplementary Table 12 Effect of inhibitors DESeq2 overlap.

Supplementary Table 13 Microglia homeostatic versus *ex*AM MAST summary results.

Supplementary Table 14 Microglia homeostatic versus chemokine MAST summary results.

Supplementary Table 15 Microglia homeostatic versus Ifn-responsive MAST summary results.

Supplementary Table 16 Microglia homeostatic versus proliferative MAST summary results.

Supplementary Table 17 Microglia homeostatic versus Mac/Mono MAST summary results.

Supplementary Table 18 Differential abundance (all CNS; mouse).

Supplementary Table 19 All CNS cells DESeq2 sig. genes.

Supplementary Table 20 All CNS microglia versus *ex*AM MAST summary results.

Supplementary Table 21 Comparison and overlap with other publications.

Supplementary Table 22 Postmortem LIGER factors related to mouse signature.

Supplementary Table 23 Fresh 0 h versus 6 h DEG microglia factor.

Supplementary Table 24 Fresh 0 h versus 6 h DEG astrocyte factor.

## Data Availability

Raw sequencing data for all mouse samples were deposited in the NCBI GEO database under the SuperSeries GSE152184 which contains the following subseries: GSE152183 (Mouse microglia four dissociation protocols), GSE152182 (Mouse all CNS cells), GSE152210 (Mouse microglia PBS tail vein), GSE188441 (Mouse microglia 10X version comparison). Cell Ranger output files are available as supplementary files via GEO and raw fastq files can be accessed from SRA linked from GEO records. Raw sequencing data for postmortem human tissue were deposited in the NCBI GEO database under the SuperSeries GSE152184, in the subseries GSE157760. Cell Ranger output files are available as supplementary files via GEO and raw fastq files can be accessed from SRA linked from GEO records. Raw sequencing data for the acutely isolated human tissue were deposited in the European Phenome-Genome Archive (EGA) (accession ID: EGAD00001008541). Raw sequencing data for the mock ‘digestion’ of human PBMCs were deposited in the Database of Genotypes and Phenotypes (dbGaP) (accession ID: phs002222.v2.p1).

## Code availability

All analysis code required to replicate Seurat or LIGER objects used in analysis can be found at: https://github.com/samuel-marsh/Marsh_et-al_2022_scRNAseq_Dissociation_Artifacts
