# Author Correction: Dissection of artifactual and confounding glial signatures by single-cell sequencing of mouse and human brain

**DOI:** 10.1038/s41593-024-01855-5

**Published:** 2024-12-13

**Authors:** Samuel E. Marsh, Alec J. Walker, Tushar Kamath, Lasse Dissing-Olesen, Timothy R. Hammond, T. Yvanka de Soysa, Adam M. H. Young, Sarah Murphy, Abdulraouf Abdulraouf, Naeem Nadaf, Connor Dufort, Alicia C. Walker, Liliana E. Lucca, Velina Kozareva, Charles Vanderburg, Soyon Hong, Harry Bulstrode, Peter J. Hutchinson, Daniel J. Gaffney, David A. Hafler, Robin J. M. Franklin, Evan Z. Macosko, Beth Stevens

**Affiliations:** 1 F.M. Kirby Neurobiology Center, Boston Children’s Hospital, Boston, MA USA; 2 Harvard Medical School, Boston, MA USA; 3 Stanley Center for Psychiatric Research, Broad Institute of MIT and Harvard, Cambridge, MA USA; 4 Wellcome–Medical Research Council Cambridge Stem Cell Institute, Cambridge Biomedical Campus, University of Cambridge, Cambridge, UK; 5 Department of Neurology and Department of Immunobiology, Yale School of Medicine, New Haven, CT USA; 6 UK Dementia Research Institute, University College London, London, UK; 7 Department of Clinical Neurosciences, University of Cambridge and Cambridge University Hospitals NHS Foundation Trust, Cambridge, UK; 8 Wellcome Sanger Institute, Wellcome Genome Campus, Hinxton, UK; 9 Broad Institute of MIT and Harvard, Cambridge, MA USA; 10 Department of Psychiatry, Massachusetts General Hospital, Boston, MA USA; 11 Howard Hughes Medical Institute, Boston Children’s Hospital, Boston, MA USA

**Keywords:** Neuroimmunology, Microglia, Gene expression analysis

Correction to: *Nature Neuroscience* 10.1038/s41593-022-01022-8, published online 8 Marcxh 2022.

This article was originally published under standard Springer nature license (© The Author(s), under exclusive licence to Springer Nature America, Inc.). It is now available as an open-access paper under a Creative Commons Attribution 4.0 International license, © The Author(s). The error has been corrected in the HTML and PDF versions of the article.

