# Supplementary material for: Dissection of artifactual and confounding glial signatures by single-cell sequencing of mouse and human brain
Source: Nat Neurosci. 2022 Mar 8;25(3):306–16. doi: 10.1038/s41593-022-01022-8 (PMC11645269; doi:10.1038/s41593-022-01022-8)
Supplement: Supplementary file 1 — Supplementary Information Table of contents, Supplementary Figs. 1–16, Notes 1–9 and references. [file 41593_2022_1022_MOESM1_ESM.pdf]

nature portfolio

https://doi.org/10.1038/s41593-022-01022-8

Supplementary information

Dissection of artifactual and confounding glial signatures by single-cell sequencing of mouse and human brain

In the format provided by the authors and unedited

Table of Contents (SI Information; Marsh et al.):

	Pages
Supplemental Figures 1-16	1-18
Supplemental Notes & Discussion 1-9	19-22
Supplemental References	22-24

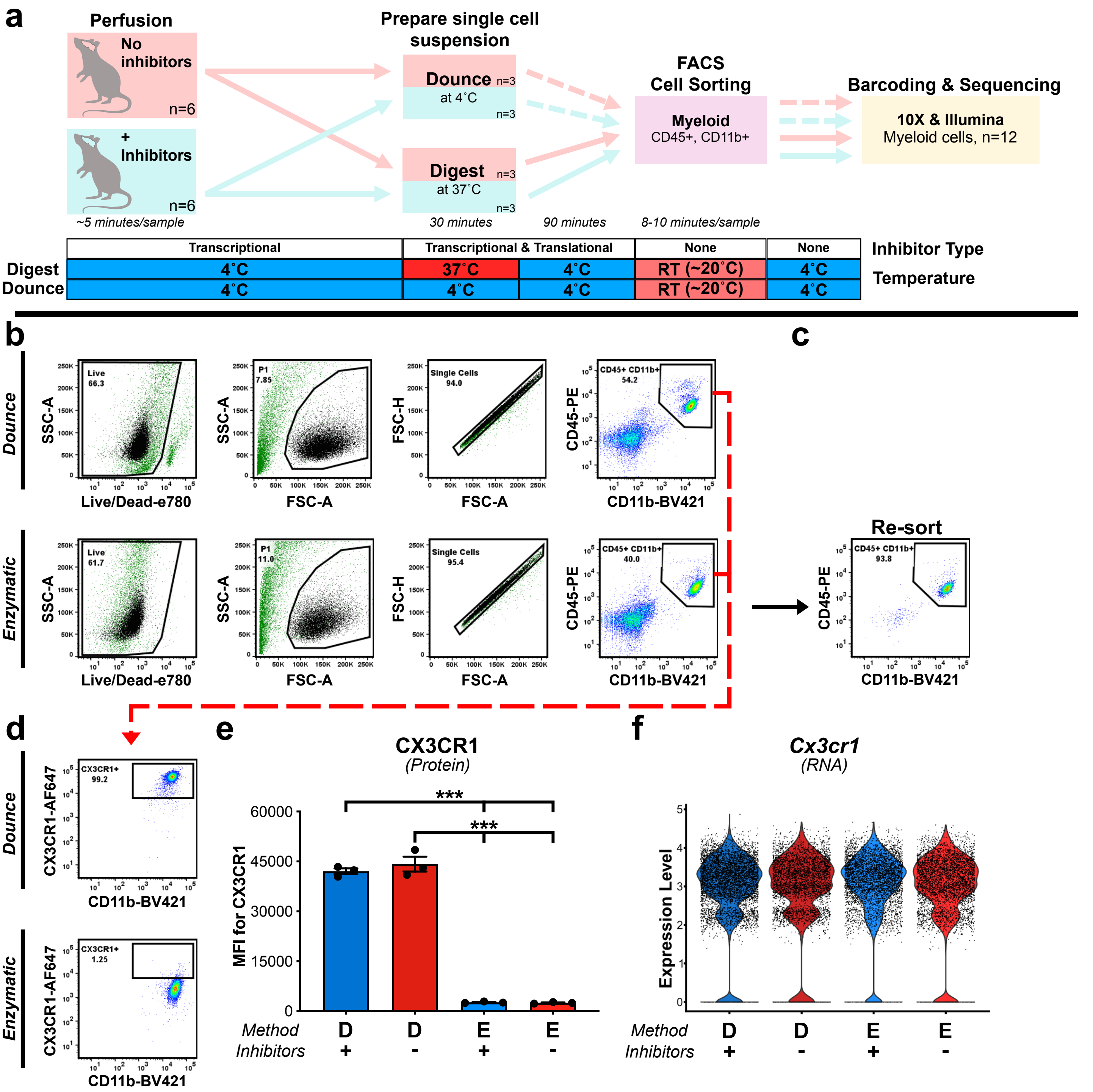

Supplemental Figure 1: FACS gating strategy for single cell sequencing and differential protein expression based on digestion protocol. a. Experimental design for sorted mouse microglia experiment (**Methods**). Mice were perfused with or without the inhibitor cocktail (n=6 per group). Groups were then further subdivided into enzymatic vs. mechanical Dounce digestion for total of 4 experimental groups (n=3/group). **b.** FACS gating strategy for scRNA-seq. Cells were first gated using Live/Dead stain, subsequently gated on overall size/granularity (FSC-A vs. SSC-A), and singlets (FSC-A vs. FSC-H). Cells were then sorted using dual positive CD45 and CD11b gate. **c.** Reanalysis of test sorted sample to examine purity of the sort. **d.** Flow plots from the sort gate showing expression of myeloid marker CX3CR1 vs. CD11b. **e.** Plot of median fluorescent intensity (MFI) for CX3CR1 across all samples. *** $p<0.0001$ of DNC-INHIB compared to either ENZ-INHIB or ENZ-NO-INHIB and DNC-NO-INHIB compared to either ENZ-INHIB or ENZ-NO-INHIB; two-way ANOVA with Tukey's multiple comparisons test post-hoc. n=3 independent samples (mice) per experimental condition. Data are presented as mean values ± SEM. **f.** Gene expression of *Cx3cr1* across all groups. **See SI Note 1** for discussion of CX3CR1 results and impact of enzymatic digestion on extracellular proteins and implications for analysis of cell phenotype.

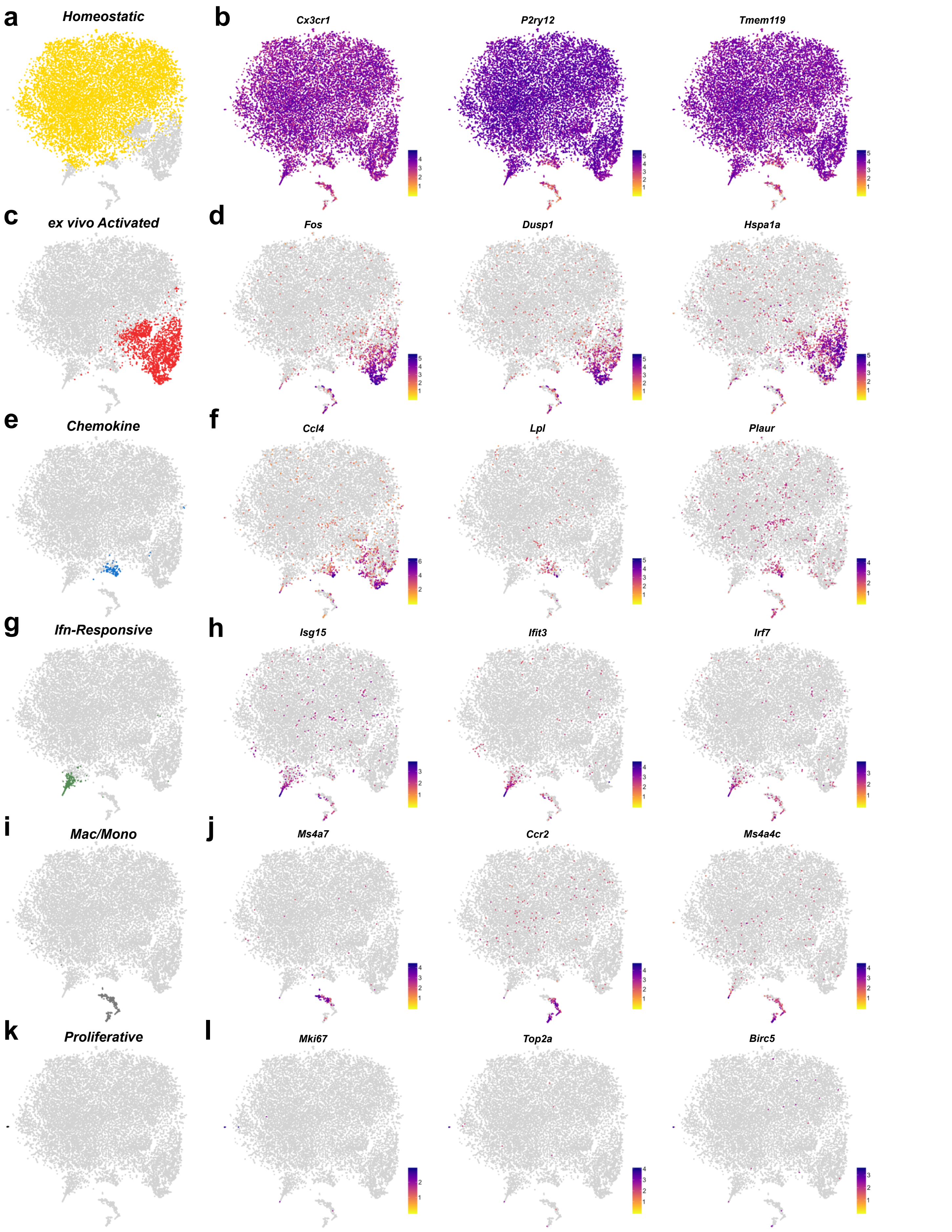

Supplemental Figure 2: Cluster specific marker gene expression for mouse microglia clusters. a, c, e, g, i, k tSNE plots with cells assigned to specific clusters, highlighted using same colors as in **Figure 1b**. **b, d, f, h, j, l** gene expression of cluster specific markers on tSNE coordinates.

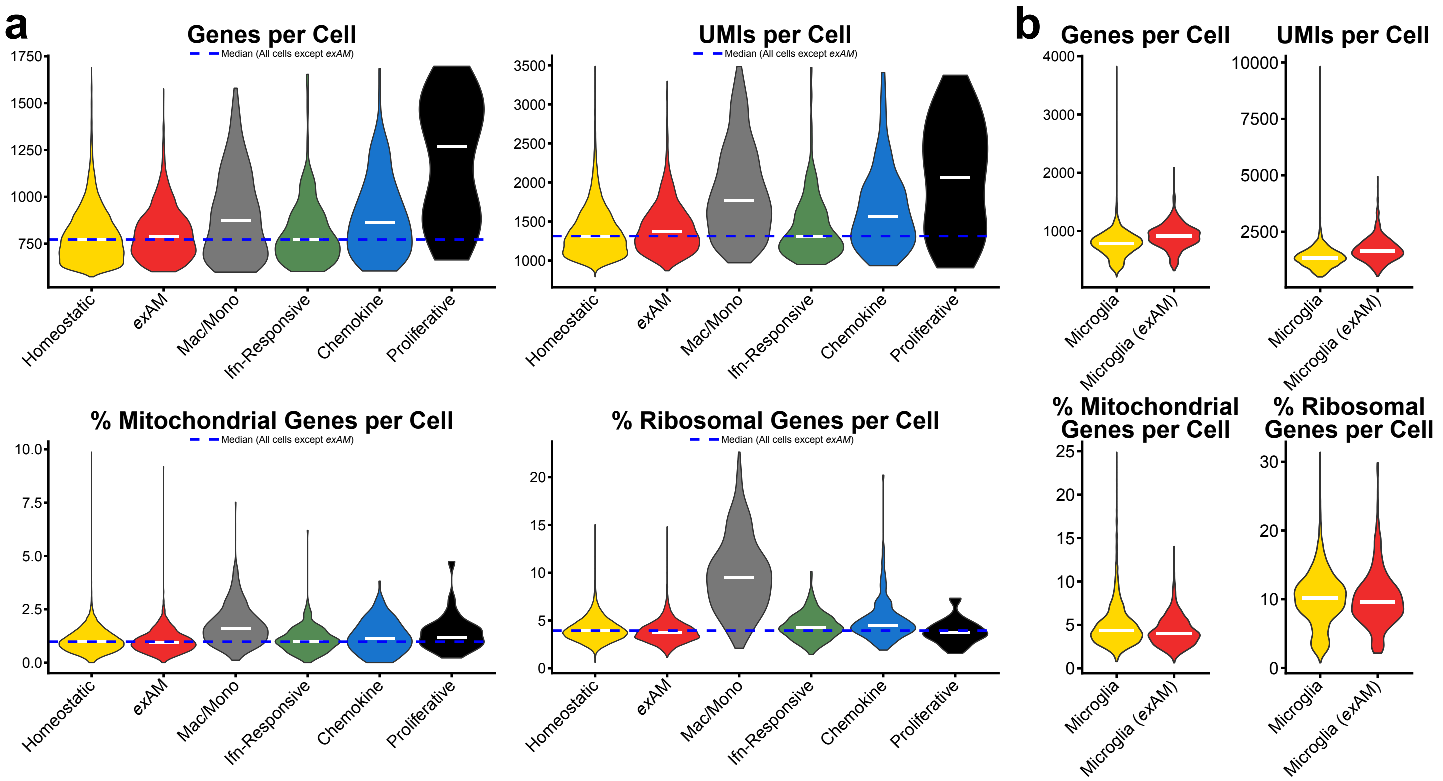

Supplemental Figure 3: *ex vivo* activated microglia are not low-quality cells. a. Plots of basic scRNA-seq quality metrics per cell (Number of genes, Number of unique transcripts (UMIs), Percentage of reads aligned to mitochondrial genes, Percentage of genes aligned to ribosomal genes) in each cluster for the data from sorted myeloid cells (**Figure 1, Supplemental Figure 1-2, 6, 9**). **b.** Same plots as in **a.** except for the microglia from all CNS cell type analysis **(Figure 2, Supplemental Figure 12-13)**. Dashed blue line represents median value for all cells except those annotated as *ex*AM, white bars represent median within each cluster.

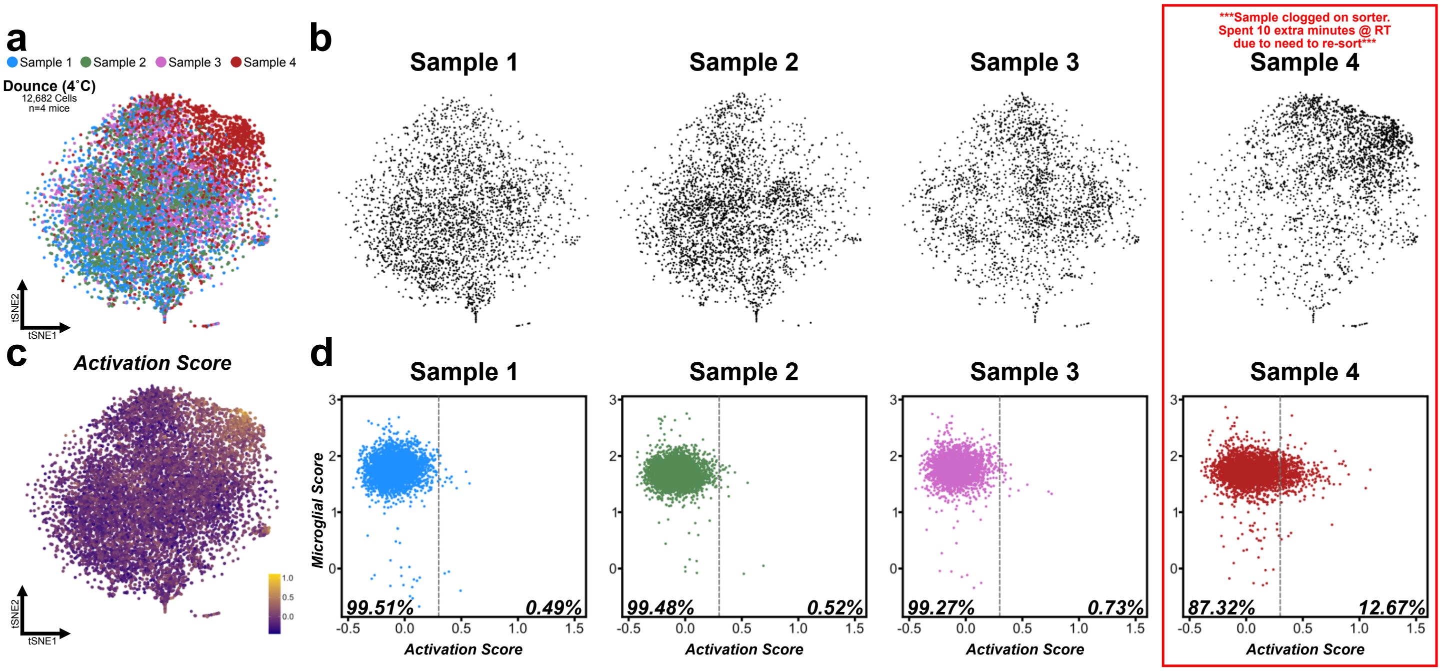

Supplemental Figure 4: Other factors that can induce *ex vivo* alterations in gene expression even when using cold mechanical Dounce homogenization. a. tSNE plot for an additional mouse microglia dataset of 12,682 cells from n = 4 mice that were injected with PBS via tail-vein 24 hours before sacrifice (control group), colored by replicate. **b.** Individual tSNE plot split by sample. **c.** Visualization of gene module scoring results using consensus *ex*AM microglial activation score **(see SI Table 4)** projected onto tSNE coordinates. **h.** Individual scatterplots by sample of the consensus *ex*AM activation score vs. the microglial identity score **(see SI Table 4)**. **See SI Note 2** for discussion of importance of biological replicates and controlling for variance even in optimized protocols.

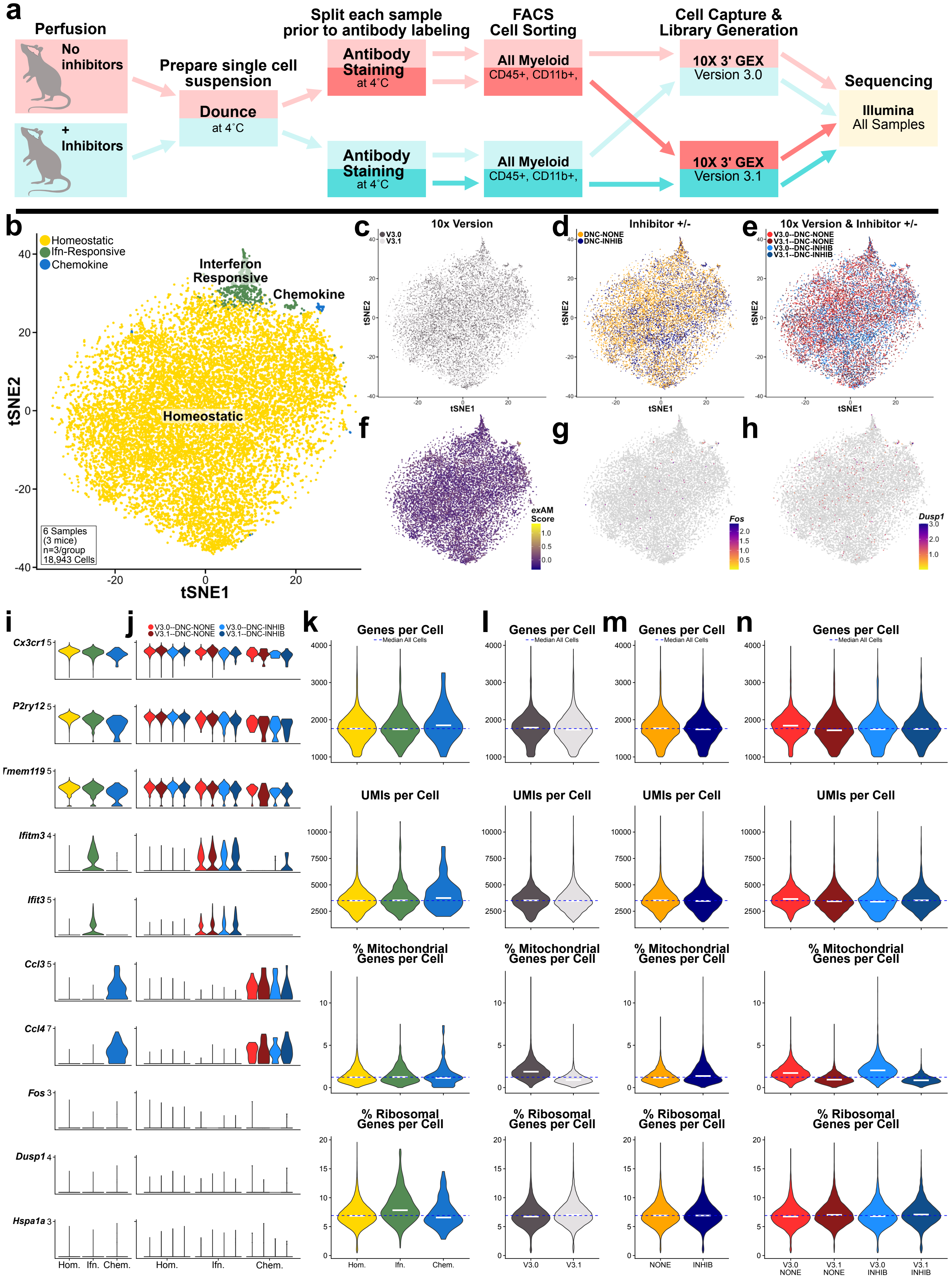

a
Perfusion
No inhibitors
+ Inhibitors
Prepare single cell suspension
Dounce
at 4˚C
Split each sample prior to antibody labeling
Antibody Staining at 4˚C
FACS Cell Sorting
All Myeloid CD45+, CD11b+,
Cell Capture & Library Generation
10X 3' GEX Version 3.0
10X 3' GEX Version 3.1
Sequencing
Illumina All Samples
b
Homeostatic
Ifn-Responsive
Chemokine
Interferon Responsive
6 Samples (3 mice) n=3/group 18,943 Cells
tSNE1
tSNE2
c
10x Version
V3.0
V3.1
d
Inhibitor +/-
DNC-NONE
DNC-INHIB
e
10x Version & Inhibitor +/-
V3.0--DNC-NONE
V3.1--DNC-NONE
V3.0--DNC-INHIB
V3.1--DNC-INHIB
f
exAM Score
g
Fos
h
Dusp1
i
j
Cx3cr1
P2ry12
Tmem119
Ifitm3
Ifit3
Ccl3
Ccl4
Fos
Dusp1
Hspa1a
Hom.
Ifn.
Chem.
k
l
m
n
Genes per Cell
Median All Cells
UMIs per Cell
% Mitochondrial Genes per Cell
% Ribosomal Genes per Cell
V3.0
V3.1
NONE
INHIB
V3.0 NONE
V3.1 NONE
V3.0 INHIB
V3.1 INHIB

Supplemental Figure 5: Comparison of scRNA-seq of microglia generated with 10X Genomics V3.0 vs. V3.1. **a.** Experimental design for the 10X version comparison experiment **(See Methods)**. **b.** tSNE plot of 18,943 microglia cells annotated by cluster. **c-e.** tSNE plots of microglia colored by different experimental conditions showing good integration of data from all experimental conditions **c.** 10X version, **d.** +/- inhibitor cocktail, **e.** 10X version & +/- inhibitor cocktail. **f-h.** Analysis of *ex*AM signature shows no induction of *ex*AM signature either via **f.** gene module scoring or **g-h.** specific marker genes *Fos* and *Dusp1*. **i.** Plots of cluster marker gene expression and *ex*AM marker genes grouped (colored) by cluster. **j.** Expression plots for the same genes as in **i.** except grouped (colored) by 10X version & inhibitor status. **k-n.** Basic scRNA-seq cell quality metrics plotted by **k**. cluster, **l.** 10X version, **m.** +/- inhibitor cocktail, **n.** 10X version & +/- inhibitor cocktail.

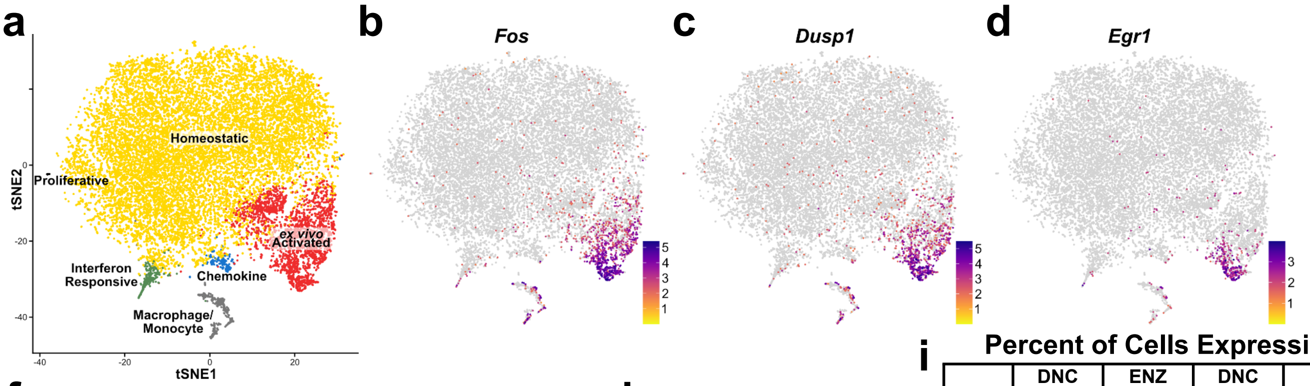

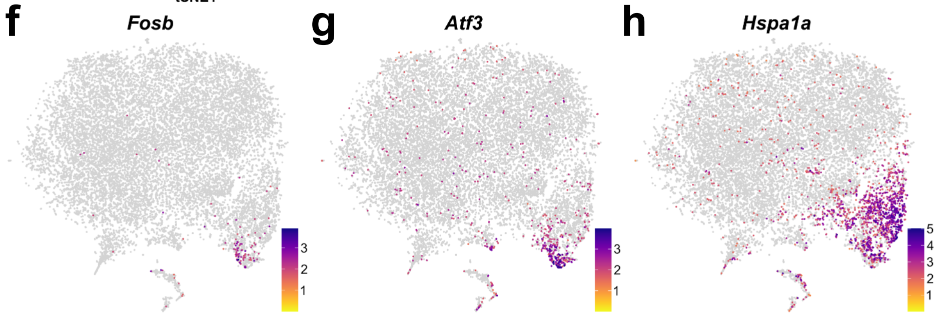

i **Percent of Cells Expressing**

	DNC NONE	ENZ NONE	DNC INHIB	ENZ INHIB
Fos	1.78%	25.47%	0.20%	0.30%
Fosb	0.20%	2.24%	1.19%	0.02%
Dusp1	0.95%	21.27%	0.73%	0.85%
Hspa1a	0.52%	35.19%	1.45%	0.85%
Egr1	0.74%	7.94%	0.24%	0.26%
*Atf3**	1.22%	8.60%	1.19%	1.68%

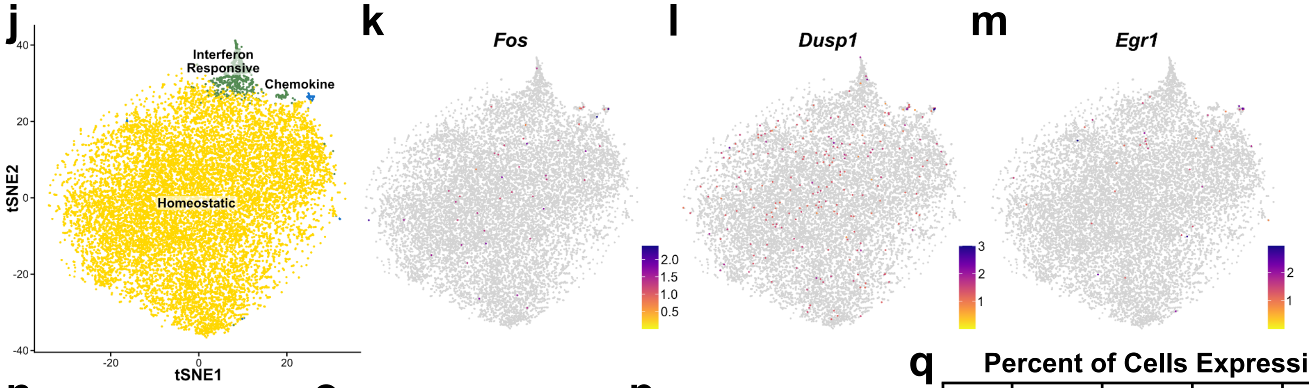

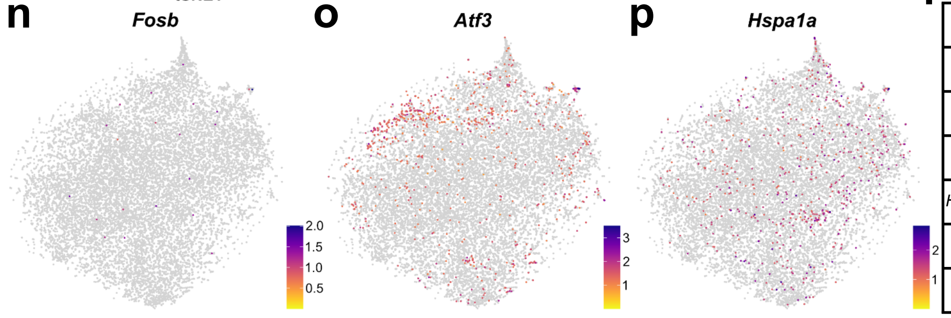

q **Percent of Cells Expressing**

	V3.0 NONE	V3.1 NONE	V3.0 INHIB	V3.1 INHIB
Fos	0.23%	0.14%	0.39%	0.36%
Fosb	0.12%	0.17%	0.05%	0.03%
Dusp1	1.38%	0.84%	1.51%	1.08%
Hspa1a^	0.82%	1.79%	5.96%	5.24%
Egr1	0.28%	0.21%	0.12%	0.21%
*Atf3**	4.06%	4.07%	3.76%	4.95%

r **Percent of Cells Expressing *Hspa1a* > 2**

	DNC NONE	ENZ NONE	DNC INHIB	ENZ INHIB
V2	0.41%	31.65%	1.11%	0.57%
	**V3.0 NONE**	**V3.1 NONE**	**V3.0 INHIB**	**V3.1 INHIB**
V3/3.1	0.02%	0.07%	0.34%	0.27%

Supplemental Figure 6: Many genes in *ex*AM signature are very lowly expressed outside of *ex vivo* contexts. a-h. Cluster annotation and expression plots for experiment containing sorted myeloid cells (**Figure 1, Supplemental Figure 1-3**). **b-h.** Individual gene expression plots demonstrate that expression of these markers is nearly exclusive to the *ex*AM cluster and not expressed when cells are isolated using optimal methods. **i**. Table displaying the percentage of cells that have non-zero expression of genes in **b-h**. per dissociation protocol. *While also being dramatically increased in conditions of *ex vivo* activation *Atf3* is transcription factor expressed by specific subset of microglia and that likely explains its higher overall levels of expressing cells. Nearly all genes are not detected in 99% except for those from ENZ-NONE condition. **j-q.** Same plots and data table as for **a-i** but for the 10X comparison experiment **(Supplemental Figure 5)**. **r.** Data table showing percentage of cells that express *Hspa1a* over normalized value of 2. ^Demonstrating that despite slightly increased levels in 10X comparison experiment (particularly in one replicate) it is still not detected in 95% of cells and expression levels are far below what is observed in true *ex vivo* activation.

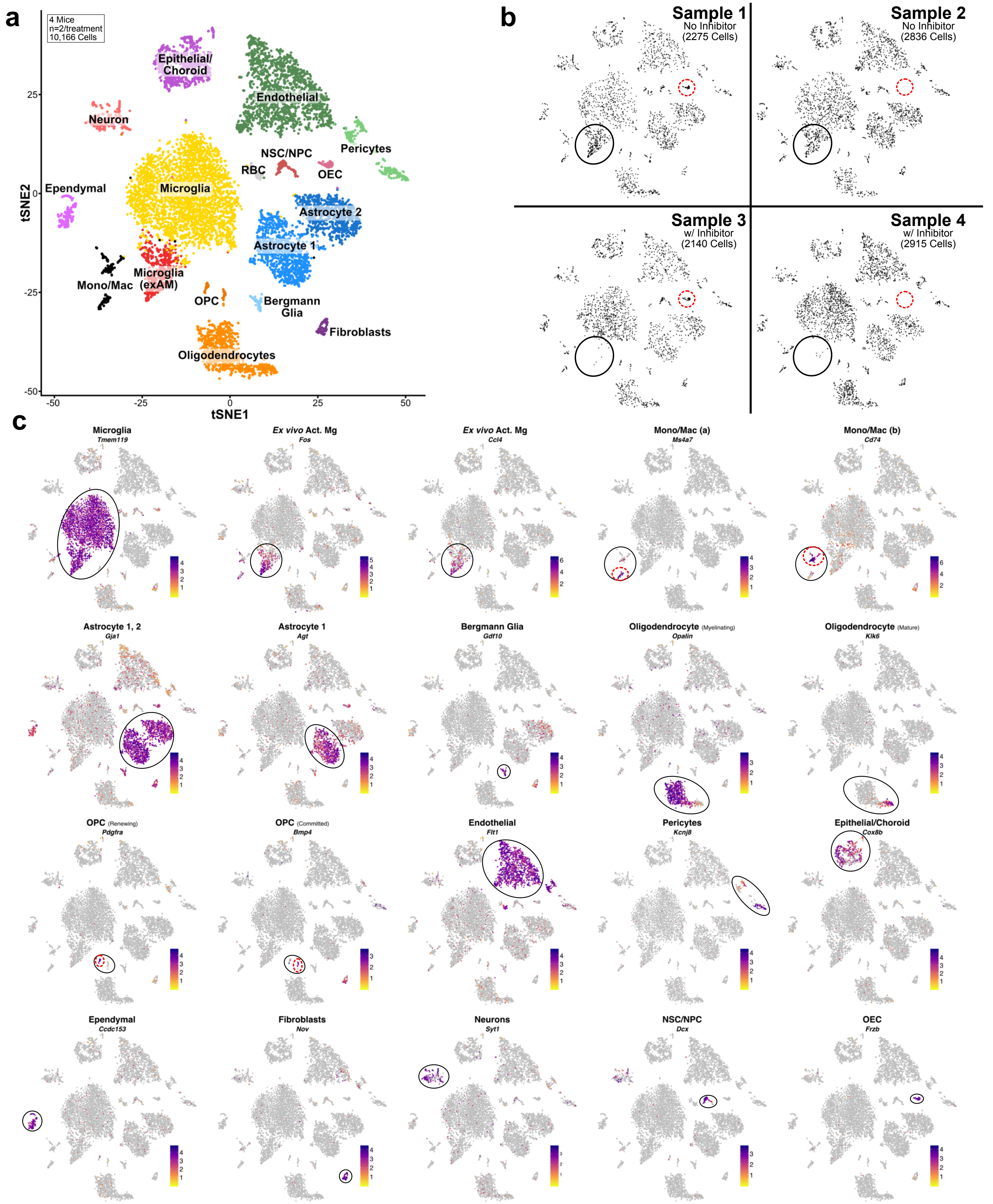

Supplemental Figure 7: Identification of brain cell types from all CNS scRNA-seq using enzymatic digestion with or without inhibitors. a. tSNE plot of 10,166 cells from 4 mice, annotated and colored by cell type from (**Figure 2b**). **b.** tSNE plots for each of the 4 replicates (n = 2/group) individually. *ex*AM cluster location (black circle), OEC cluster location (red dashed circle). **c.** Cluster specific marker gene expression plotted on tSNE coordinates for all cell types and some subtypes.

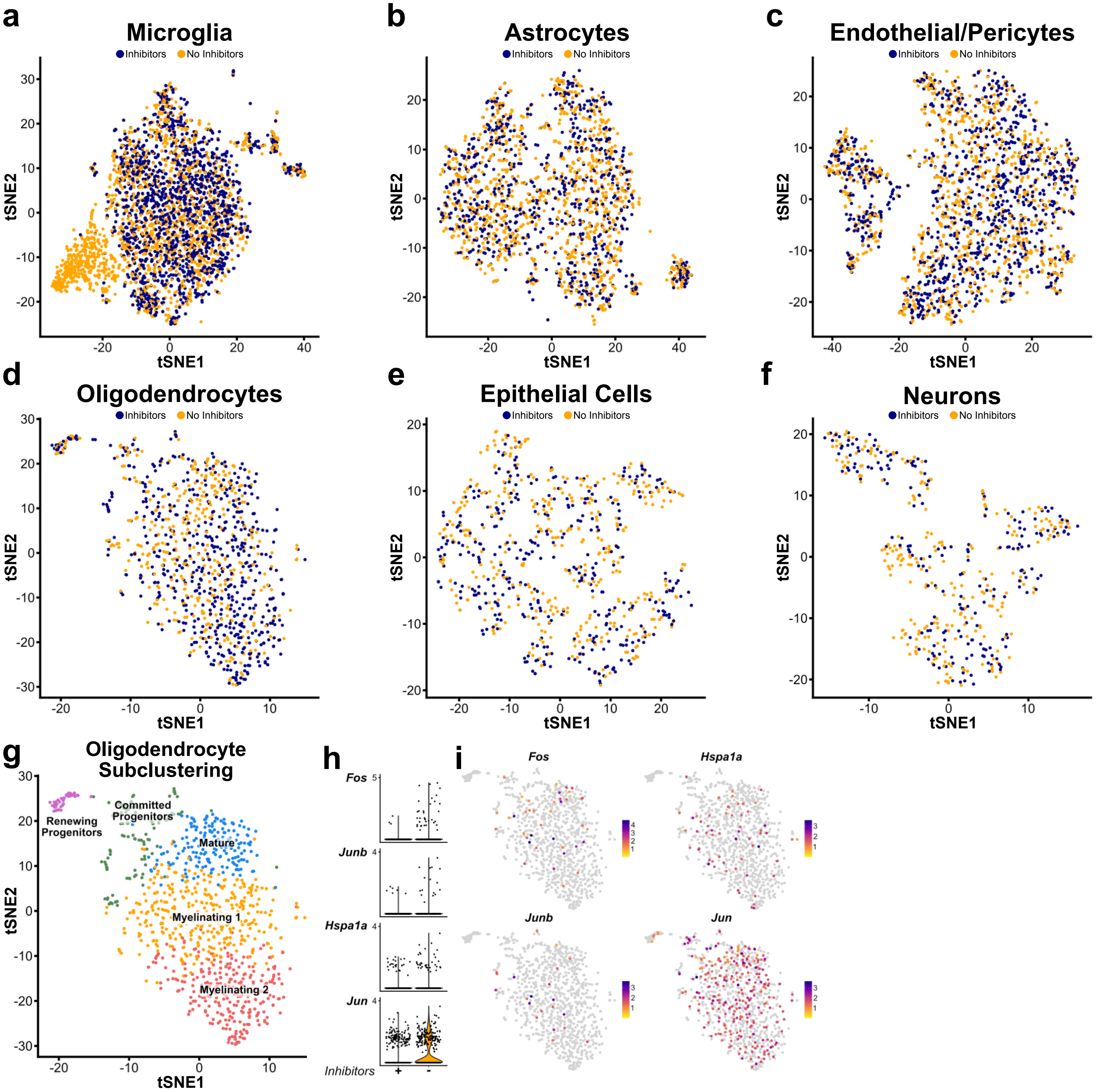

Supplemental Figure 8: Subclustering analysis confirms that microglia are particularly vulnerable to *ex vivo* artifactual gene expression during enzymatic dissociation. a-f. tSNE plots from the subclustering analysis of major cell types present in **Fig 2b**, colored by presence/absence of inhibitors. **g.** Annotation of subclustered oligodendrocyte and oligodendrocyte progenitor populations. **h.** Gene expression for a selection of genes that are part of the activation score in oligodendrocyte subcluster split by experimental group. **i**. Gene expression of genes in **h** overlaid on tSNE coordinates for oligodendrocyte subclustering.

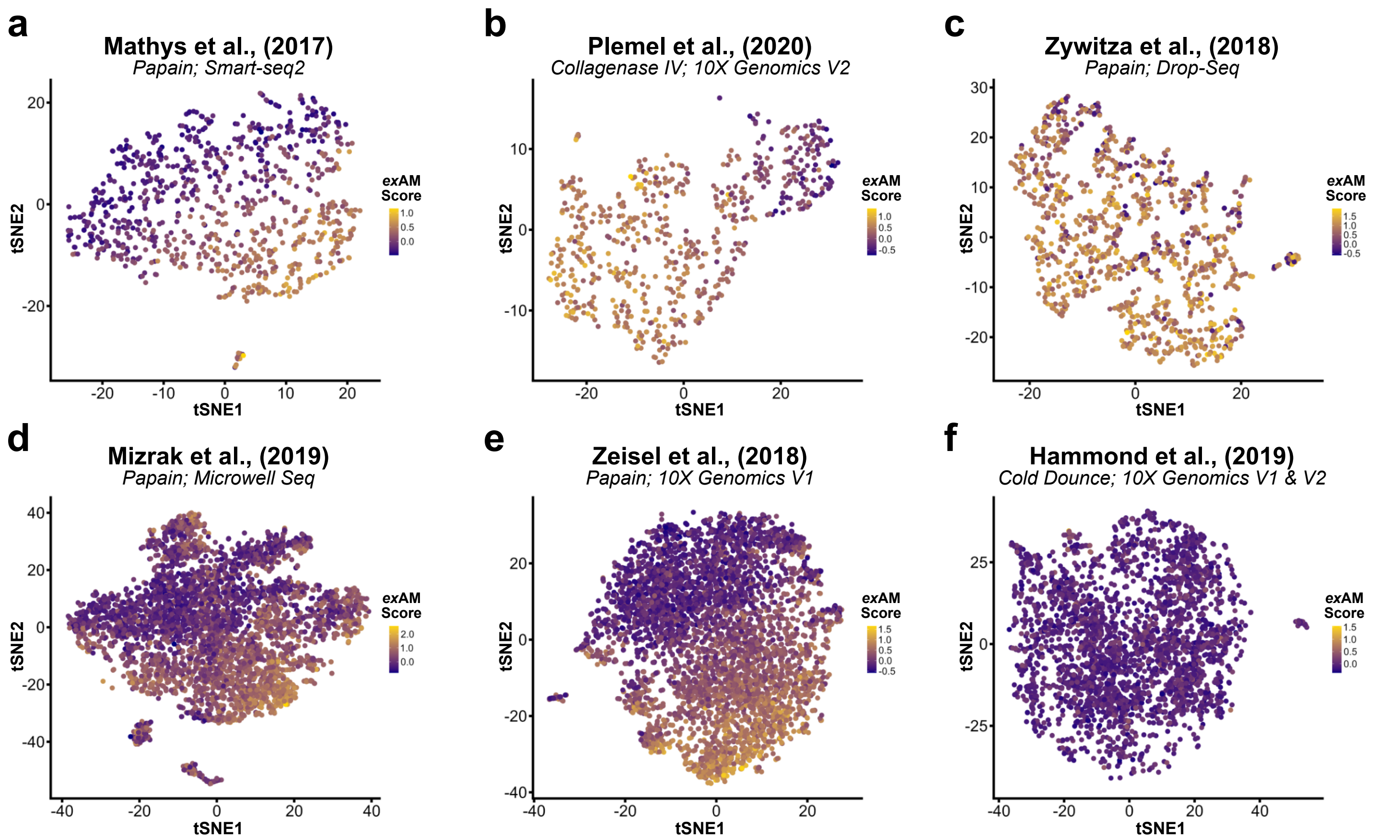

g

Literature Reanalysis

Microglia Datasets

Dataset Name	Enzyme	Seq Used	Tissue	Cell Number	GEO/SRA	Raw Counts Matrix	Citation
Mathys et al.	Papain (Miltenyi)	Smart-Seq2	Brain	883	GSE103334	Provided by Authors	Mathys et al. 2017 (Cell Reports)
Plemel et al.	Collagenase-IV	10X 3' (V2)	Spinal Cord	644	GSE115803	GEO	Plemel et al., 2020 (Science Advances)
Zywitza et al.	Papain (Miltenyi)	Drop-Seq	SVZ	1153	GSE111527	GEO	Zywitza et al., 2018 (Cell Reports)
Mizrak et al.	Papain (Worthington)	Microwell Seq	SVZ	5020	GSE109447	GEO	Mizrak et al., 2019 (Cell Reports)
Zeisel et al.	Papain (Worthington)	10X 3' (V1 & V2)	Brain	5227	SRP135960	mousebrain.org	Zeisel et al., 2018 (Cell)
Hammond et al	Dounce	10X 3' (V1 & V2)	Brain	7586	GSE121654	GEO	Hammond et al., 2019 (Immunity)

Supplemental Figure 9: *Ex vivo* artifactual gene expression occurs in brain myeloid populations when enzymatic digestion is used, across different labs, dissociation enzymes, sequencing technologies, and dissociation protocols. a-f. Visualization of gene module scoring using consensus *ex*AM microglial activation gene module score **(SI Table 4)** projected onto tSNE coordinates. **a-e.** Reanalysis of brain myeloid cells from 5 sample datasets in the published literature, spanning variety of dissociation protocols, different dissociation enzymes, and five different sequencing technologies/technology versions. **f.** Reanalysis of our lab's previously published data with cold mechanical Dounce homogenization to present activation. **g**. Table of dataset characteristics and citations for the reanalysis in **a-f**.

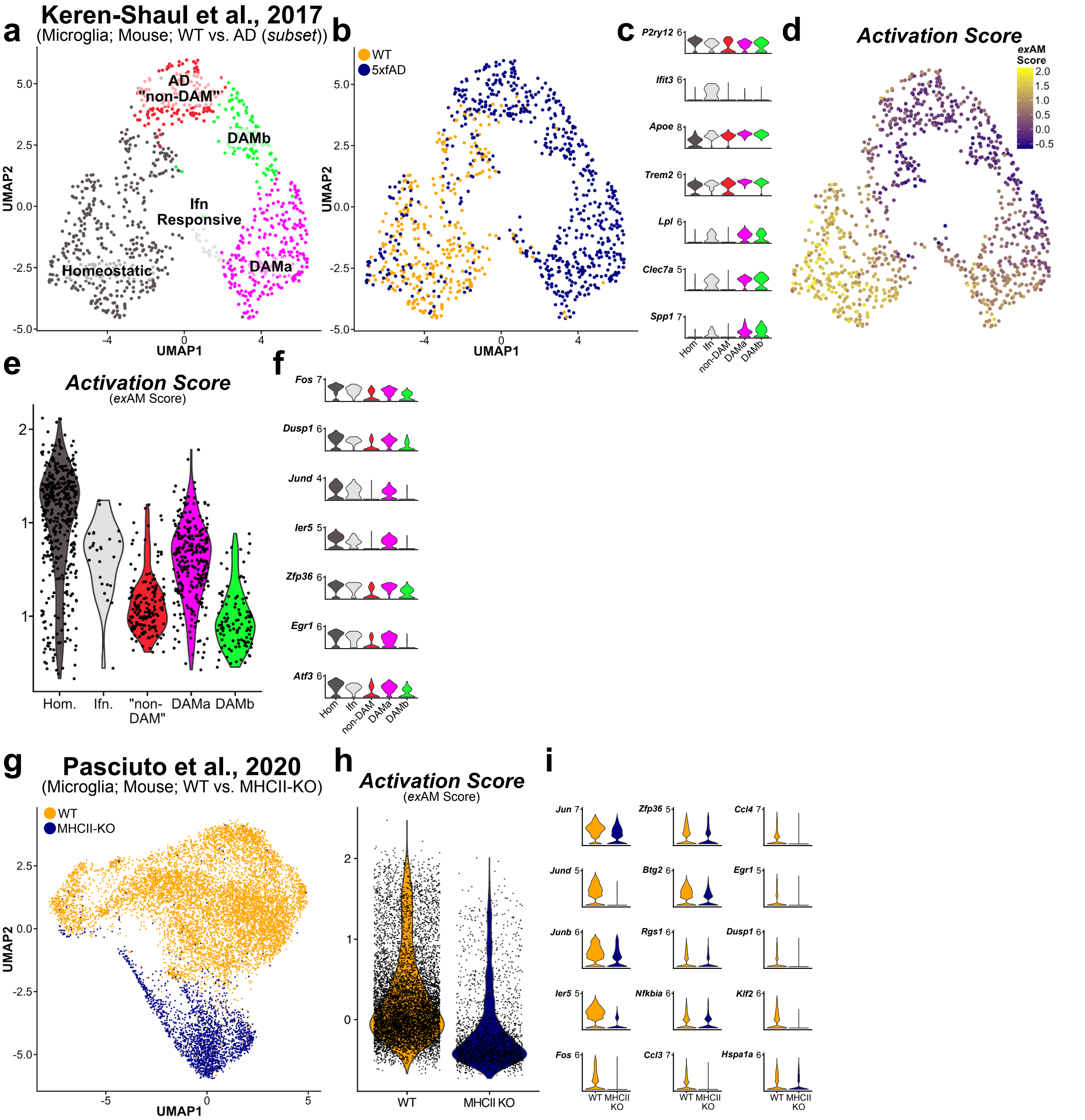

Supplemental Figure 10: Use of enzymatic digestion can alter conclusions when comparing different diseases or genotypes via scRNA-seq. **a.** UMAP plot annotating microglia from a specific subset of the data from Keren-Shaul et al., 2017. **b.** UMAP plot with cells colored by disease status (WT vs. 5xfAD). **c.** Expression of selection of cluster markers used to annotate dataset in **a.** **d-e.** Visualization of gene module scoring results using *ex*AM signature **d.** plotted on UMAP coordinates and **e.** by cluster. **f.** Gene expression of several *ex*AM cluster markers across each cluster. **g.** UMAP plot colored by genotype (WT vs. MHCII-KO) from reanalysis of microglia scRNA-seq data from Pasciuto et al., 2020. **h.** Visualization of gene module scoring results using *ex*AM signature plotted by genotype. **i.** Gene expression of several *ex*AM cluster markers plotted by genotype.

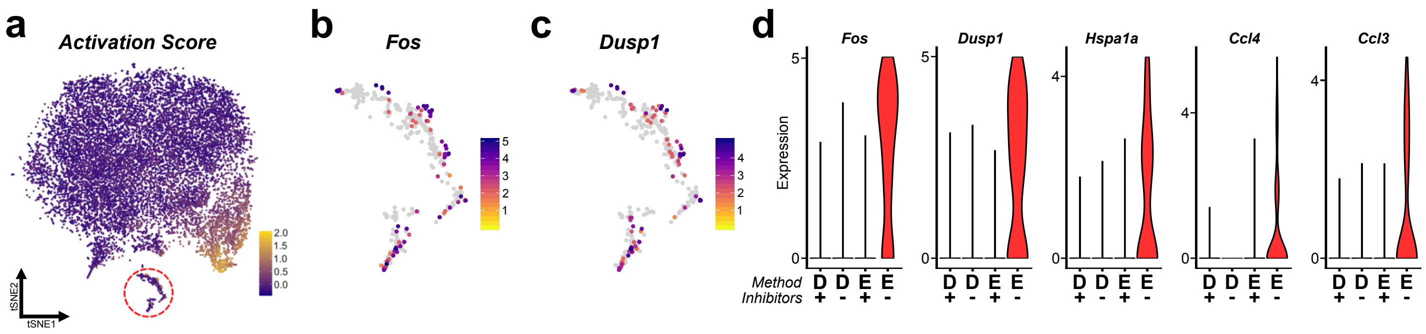

Supplemental Figure 11: Non-microglial myeloid populations also exhibit *ex vivo* activation during enzymatic digestion. **a.** tSNE plot of consensus *ex*AM signature in primary microglia/myeloid dataset (**Figure 1**), non-microglial myeloid cells highlighted (red dashed circle). **b-c.** Gene expression of *Fos* and *Dusp1* specifically in the macrophage/monocyte cluster circled in **a.** **d.** Gene expression of several *ex*AM markers split by experimental group demonstrate that increased expression of these markers only occurs in ENZ-NONE group.

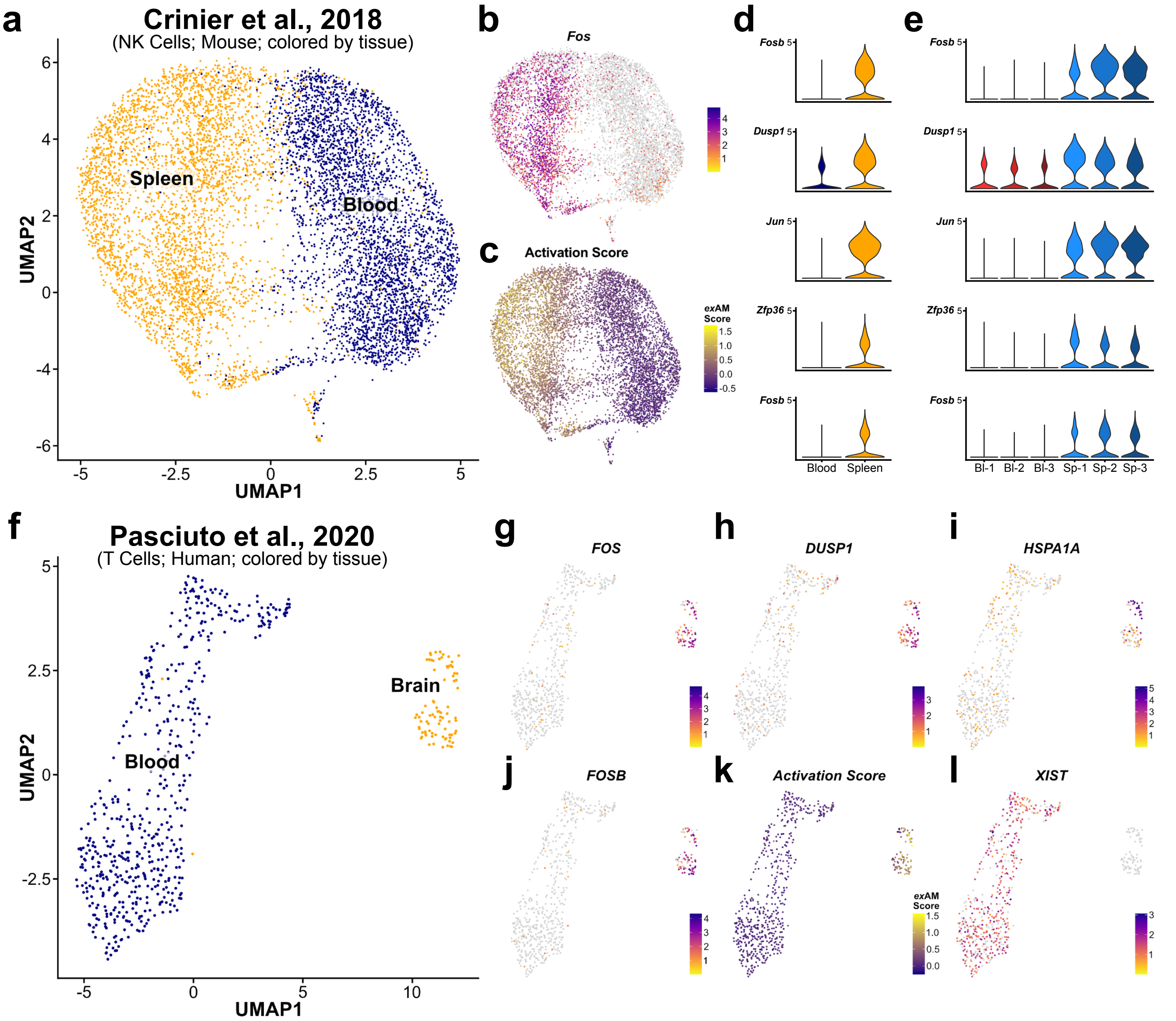

Supplemental Figure 12: Tissue dissociation biases comparative analyses of tissue resident immune populations compared to analogous circulating blood populations in mice and humans. a. UMAP plot from reanalysis of mouse NK cells from Crinier et al., 2018 colored by tissue of origin (spleen vs. blood). **b.** Visualization of canonical dissociation marker *Fos* and **c.** gene module score of activation using the *ex*AM signature projected on UMAP coordinates. **d-e.** Gene expression of several *ex*AM markers plotted by **d.** tissue of origin and **e.** sample replicate. **f.** UMAP plot of human T cells from Pasciuto et al., 2020 colored by tissue of origin (brain vs. blood). **g-j.** Gene expression of several *ex*AM markers plotted on UMAP coordinates. **k**. Visualization of gene module scoring using homologous human genes from the *ex*AM signature. **l.** Gene expression of X chromosome gene *XIST* demonstrates that blood and brain T cells were also not from the same donor.

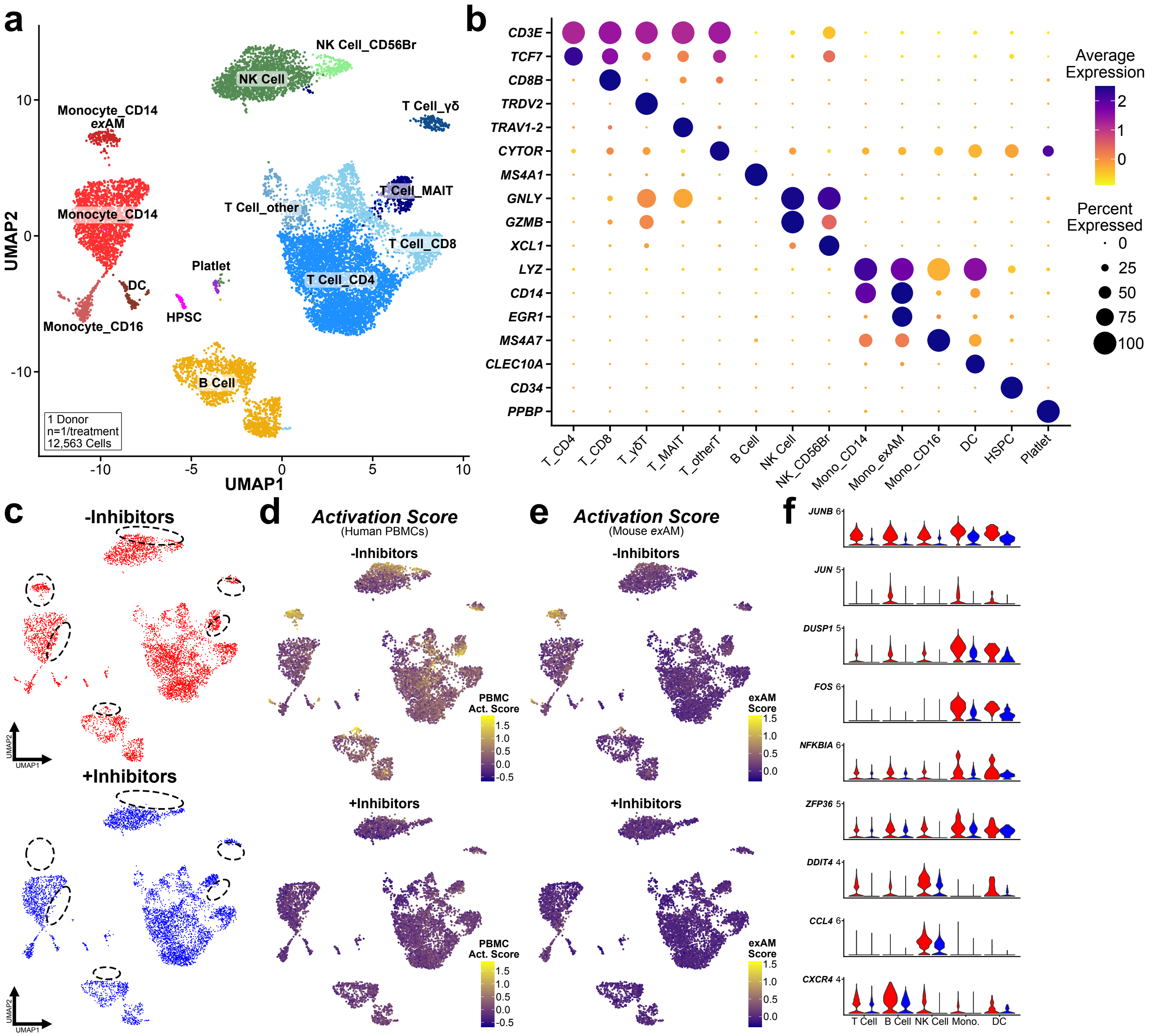

Supplemental Figure 13: Pilot experiment demonstrating the blood immune cells can upregulate dissociation-like signature when subjected to mock enzymatic digestion. **a.** Annotation of 12,563 cells from 1 donor subjected to mock digestion with or without the presence of inhibitor cocktail, colored by cell type/subtype. **b.** Dot plot visualization of the expression of selection of canonical marker genes used for cell type/subtype annotation in **a**. **c.** UMAP plot split by experimental group (+/- inhibitor cocktail) highlights areas of differential cell abundance between the experimental groups (dashed circles). **d-e.** Visualization of activation score split by experimental group. **d.** Activation score based on genes identified as DE between +/- inhibitor groups in 3 or more major cell classes. **e.** Activation score based on homologous human genes from the *ex*AM signature. **f**. Expression of genes induced by dissociation without inhibitors grouped by major cell class and split by experimental condition.

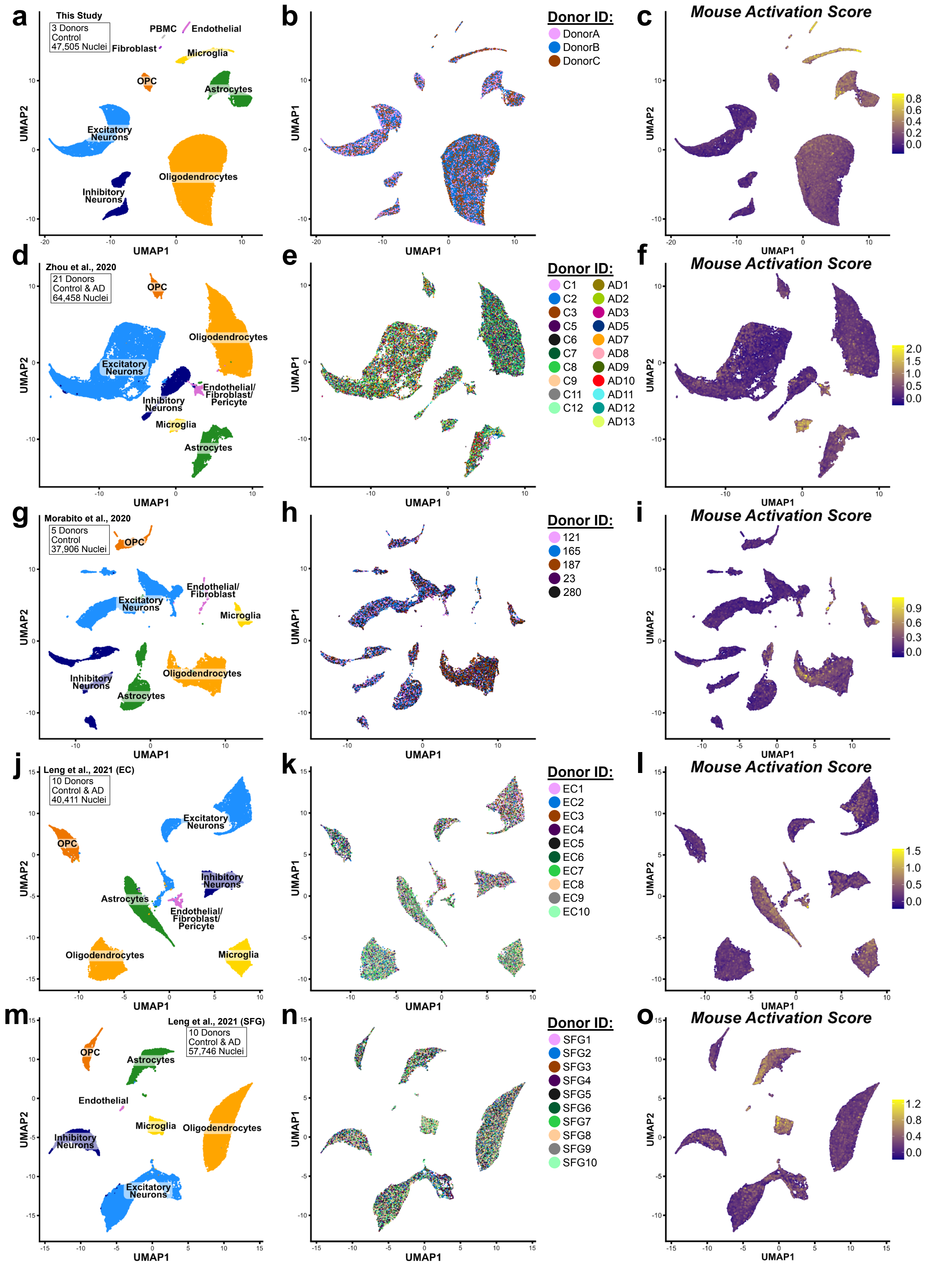
a
This Study
3 Donors
Control
47,505 Nuclei
PBMC
Endothelial
Fibroblast
Microglia
OPC
Astrocytes
Excitatory Neurons
Oligodendrocytes
Inhibitory Neurons
UMAP2
UMAP1
b
Donor ID:
DonorA
DonorB
DonorC
c
Mouse Activation Score
d
Zhou et al., 2020
21 Donors
Control & AD
64,458 Nuclei
OPC
Oligodendrocytes
Excitatory Neurons
Endothelial/ Fibroblast/ Pericyte
Inhibitory Neurons
Microglia
Astrocytes
e
Donor ID:
C1
C2
C3
C5
C6
C7
C8
C9
C11
C12
AD1
AD2
AD3
AD5
AD7
AD8
AD9
AD10
AD11
AD12
AD13
f
Mouse Activation Score
g
Morabito et al., 2020
5 Donors
Control
37,906 Nuclei
OPC
Endothelial/ Fibroblast
Excitatory Neurons
Microglia
Inhibitory Neurons
Oligodendrocytes
Astrocytes
h
Donor ID:
121
165
187
23
280
i
Mouse Activation Score
j
Leng et al., 2021 (EC)
10 Donors
Control & AD
40,411 Nuclei
Excitatory Neurons
OPC
Inhibitory Neurons
Astrocytes
Endothelial/ Fibroblast/ Pericyte
Oligodendrocytes
Microglia
k
Donor ID:
EC1
EC2
EC3
EC4
EC5
EC6
EC7
EC8
EC9
EC10
l
Mouse Activation Score
m
Leng et al., 2021 (SFG)
10 Donors
Control & AD
57,746 Nuclei
OPC
Astrocytes
Endothelial
Microglia
Oligodendrocytes
Inhibitory Neurons
Excitatory Neurons
n
Donor ID:
SFG1
SFG2
SFG3
SFG4
SFG5
SFG6
SFG7
SFG8
SFG9
SFG10
o
Mouse Activation Score

Supplemental Figure 14: snRNA-seq analysis and literature reanalysis of post-mortem human brain. Total analysis includes 248,026 nuclei comprised of all major CNS cell types across 49 samples. For each dataset plots illustrate: overall cluster annotation by major cell type, integration of samples within dataset following LIGER analysis colored by donor, and visualization of gene module scoring using activation score from mouse dataset plotted on UMAP coordinates. **a-c.** Dataset produced in the current study; **d-f.** Dataset from Zhou et al., 2020; **g-i.** dataset from Morabito et al., 2020; **j-l.** dataset from Leng et al., 2021 (EC); **m-o.** dataset from Leng et al., 2021 (SFG).

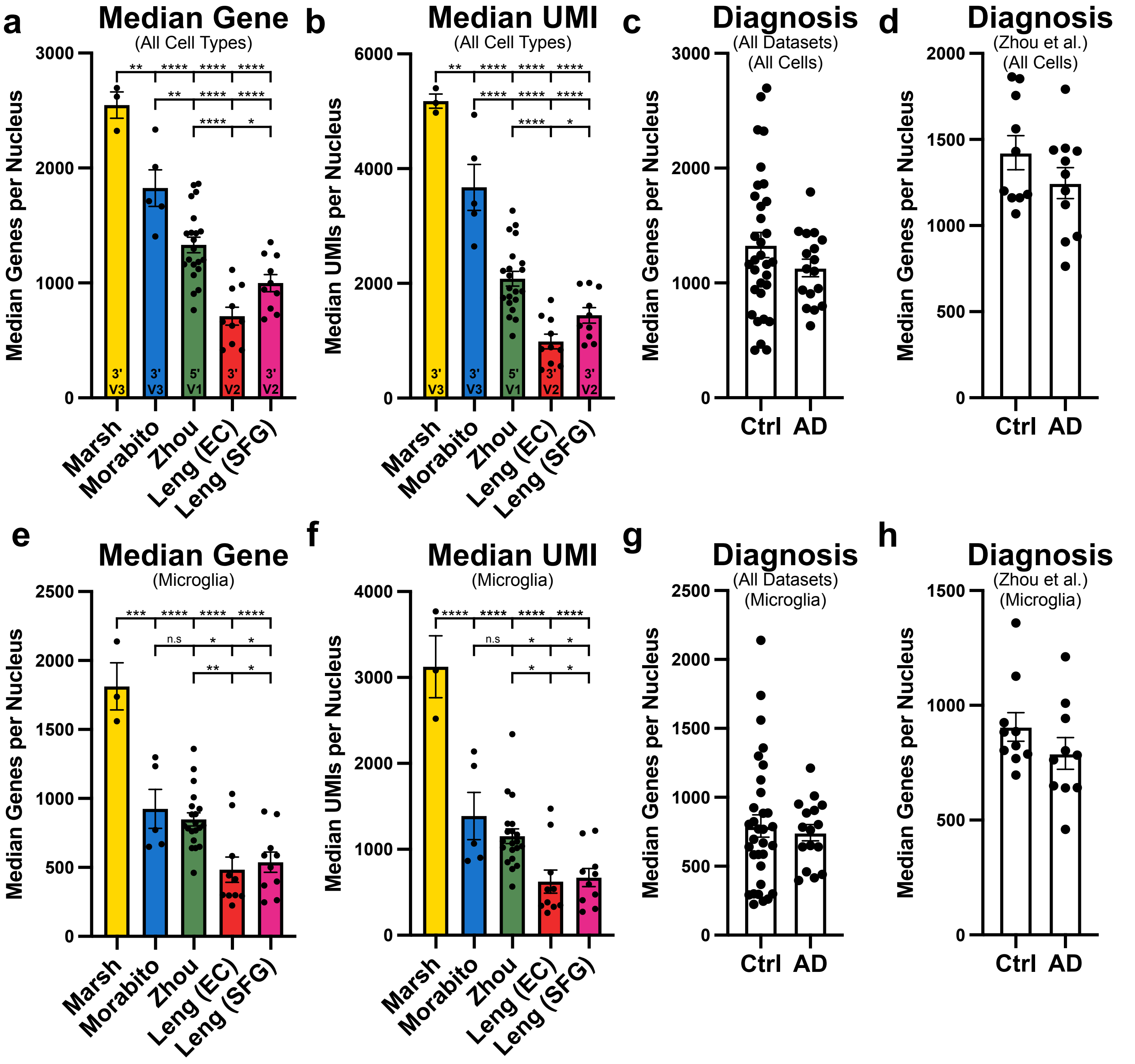

Supplemental Figure 15: Significant differences in gene and UMI detection sensitivity across datasets demonstrates impact of 10X chemistry version and nuclei isolation protocol. **a.** Median number of genes detected per nucleus for each sample across all cell types in each dataset. Version of 10X kit chemistry used in each study is listed inside bars of graph. **b.** Median number of UMIs detected per nucleus for each sample across all cell types in each dataset. Version of 10X kit chemistry used in each study is listed inside bars of graph. **c-d**. Median number of genes detected per nucleus for each sample across all cell types grouped by donor diagnosis for **c** all datasets or **d** just within Zhou et al., 2020 dataset. **e.** Median number of genes detected per nucleus for each sample in microglial nuclei in each dataset. **f.** Median number of UMIs detected per nucleus for each sample in microglial nuclei in each dataset. Version of 10X kit chemistry used in each study is listed inside bars of graph. **g-h**. Median number of genes detected per nucleus for each sample in microglial nuclei grouped by donor diagnosis for **g** all datasets or **h** just within Zhou et al., 2020 dataset. * $p<0.05$, ** $p<0.01$, *** $p<0.001$, **** $p<0.0001$; one-way ANOVA with Tukey's multiple comparisons test post-hoc. All data are presented as mean values ± SEM. **a-b.** n=49 independent samples (n=3, 5, 21, 10, 10 per study). **c.** n=49 samples (32 control/17 AD). **d.** n=21 samples (10 control/11 AD). **e-f.** n=48 independent samples (n=3, 5, 20, 10, 10 per study). **g.** n=48 samples (32 control/16AD). **h.** n=20 samples (10 control/10 AD).

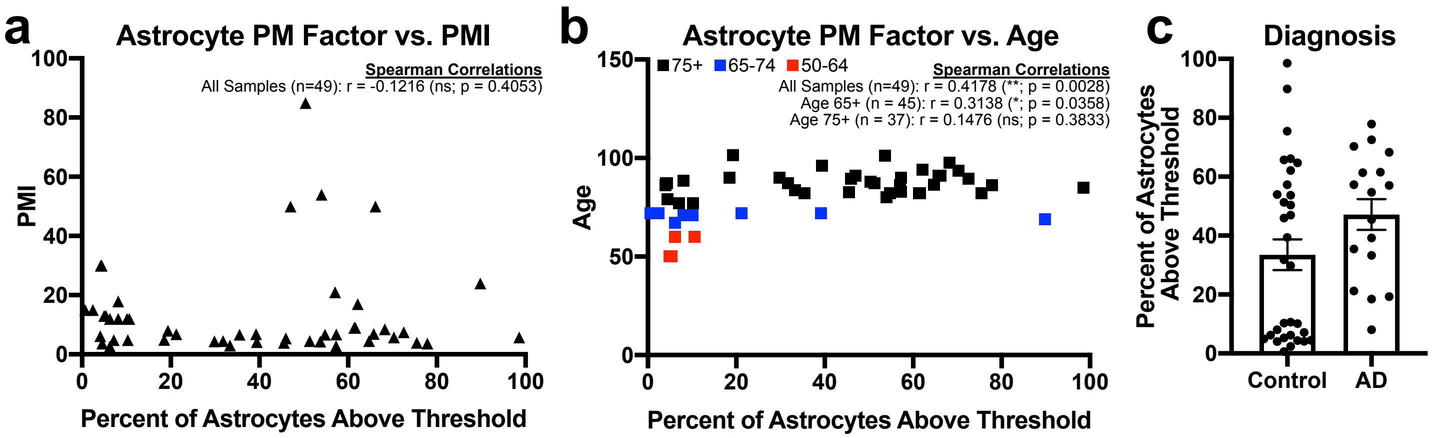

Supplemental Figure 16: Correlations between enrichment of astrocyte LIGER factor score and meta data variables in post-mortem astrocyte nuclei. a-b. Spearman correlation of PMI and age of donor vs percent of astrocytes above score threshold for astrocyte factor score in each post-mortem sample, graph annotations list Spearman r values and significance. **c.** Plot of percent of astrocytes above score threshold for astrocyte factor score in each post-mortem sample split by diagnosis. n=49 samples (32 control/17 AD). Data are presented as mean values ± SEM.

SI Note 1: Important considerations for the analysis of cell surface receptors/proteins following the use of enzymatic digestion (i.e. CITE-Seq, flow cytometry, CyTOF, etc)

In our previous study[1], in addition to CD11b and CD45, we also used canonical myeloid marker CX3CR1 during the FACS sort. However, in the current study we noticed a dramatic reduction in CX3CR1 fluorescence in samples which had been enzymatically digested and therefore excluded CX3CR1 from the sort criteria **(Supplemental Figure 1d-e)**. Post-hoc analysis found that the presence of inhibitor cocktail during dissociation had no effect on CX3CR1, but cells digested with enzymes exhibited a nearly 17 fold, or greater decrease in median fluorescence intensity (MFI) of CX3CR1 **(Supplemental Figure 1d,e)**. A large body of work in the peripheral immunology field has demonstrated that enzymatic digestion is capable of cleaving extracellular epitopes from the cell surface[2–7], which is most likely what occurred in the current experiment as well. In addition to the short time scale of the experiment, RNA expression of *Cx3cr1* was stable across all four groups indicating no global downregulation of *Cx3cr1* as a result of enzymatic dissociation **(Supplemental Figure 1f)**.

This potential caveat is further complicated by the fact that different enzymes have been found to result in cleavage of different epitopes with different affinities[2,3,5,6]. Understanding and controlling for the impact of enzymatic digestion is critical for any downstream method that measures extracellular protein levels. This is important for high-dimensional single cell-based methods that are used to complement scRNA-seq work (Flow cytometry, CyTOF, Infinity Flow)[8,9], but is perhaps most critical for high-dimensional multi-modal methods that pair examine of cell surface protein with transcriptomics (i.e. CITE-Seq) or epigenomics (i.e. ASAP-seq)[10,11]. Cleavage of extracellular epitopes by enzymatic digestion prior to CITE-Seq/ASAP-Seq may result in inaccurate linkage between RNA and protein levels for a particular gene/protein and analysis of such situations requires special care to control for these potential confounding effects. Similarly, proteomic-based analyses making comparisons between cells from tissues digested with different enzymes[12,13] or between cells from undigested tissue (i.e. blood) vs. digested tissues[14] need to be interpreted with caution.

SI Note 2: Importance of consistent tissue processing and biological replicates to enable identification of technical artifacts

While proper cold Dounce homogenization is sufficient to prevent ex vivo activation it is not infallible to experiment-specific artifacts depending on conditions. To demonstrate potential issues that may arise in course of normal experiment, we performed an analysis of a dataset we previously disregarded due to a technical issue that also used cold Dounce mechanical homogenization. This dataset consisted of the control group from an unrelated experiment (adult C57Bl6/J mice received tail vein injection of PBS 18 hours before perfusion and cell isolation). Following QC, this dataset consisted of 12,682 cells from n=4 mice. We noted that during the FACS sort of the final mouse (Sample 4) there was a clog in the sorter. Previously sorted cells were discarded and sample was placed back on ice while unclogging and cleaning was performed, before beginning the sort anew. This disruption meant that these cells went from ice to room temperature during first sort, back on ice, and then back to room temperature for the resort. Due to either extra time at room temperature, the extra set of temperature changes, or combination of factors we found that Sample 4 to exhibit significantly different characteristics compared to the first 3 samples.

When visualized by plotting the contribution of each sample to the overall tSNE plot, we observed an enrichment of Sample 4 in one corner of the plot **(Supplemental Figure 4a-b)**. To determine whether this enrichment represented ex vivo activated cells, we performed gene module scoring using the consensus exAM signature. Plotting of this score revealed that the area enriched for Sample 4 was also enriched for cells positive for the activation signature **(Supplemental Figure 4c)**. We further confirmed that the cells exhibiting exAM enrichment were almost exclusively limited to Sample 4 **(Supplemental Figure 4d)**. This finding demonstrated that even what might be considered minor deviations during cell isolation and sorting, can induce significant alterations in the transcriptome of microglia. This result also reinforces the need for well powered scRNA-seq experiments, including the need for independent biological replicates for each condition being examined, which is not always the case in the current scRNA-seq literature. Without proper replicates experiment-specific variations, such as the one described here, could be incorrectly attributed to the condition/treatment/genotype being examined as opposed to technical/batch effect.

This result also demonstrates that prolonged FACS or other room temperatures protocol steps may be sufficient to induce aspects of the dissociation signature. In fact, prior TRAP-Seq/Ribo-Tag studies of microglia found that many of the genes in our exAM signature were only expressed in the digested and sorted microglia (including transcription factors like *Fos* and *Egr1*)[15–17]. However, it should be noted that some have subsequently implied that induction of these genes was the result of the use of FACS to sort the cells as final step in the cell isolation, and not the digestion of tissue itself. However, as we demonstrate in the current study, either cold mechanical dissociation with or without inhibitors or enzymatic digestion with inhibitors followed by FACS-

does not induce this signature, and the signature was also present in our analysis of all CNS cell types which did not undergo FACS. Indicating that it was most likely the enzymatic digestion plus increased temperature that was used to digest tissue used in the comparison to Ribo-Tag/TRAP-seq that likely induced the *ex*AM signature[15–17]. Although, as we show, care does need to be taken during the FACS step as some level of induction is possible under certain conditions. While there are drawbacks to the TRAP-seq/Ribo-Tag approach, we do believe they represent one of the better bulk-sequencing methods by which to examine microglia gene signatures in a state that more closely reflects their *in situ* profile.

SI Note 3: Presence of aberrant dissociation signature in microglia of scRNA-seq atlas studies confounds interpretation of microglial heterogeneity.

A careful understanding of cell type/sub-type abundance can be an incredibly powerful measurement in scRNA-seq datasets. However, these "atlas" analyses are predicated on the cell states being analyzed reflecting true *in vivo* states. Therefore, the existence of additional clusters of cell states, like we find in our own data (**Figure 1b,c,e & Figure 2b,d,e**), and many previously published "atlas" datasets, can potentially confound both analyses of cell type abundance in addition to masking true *in vivo* signatures.

SI Note 4: Presence of artificial dissociation signature in microglia can significantly impact downstream analysis/conclusions in case-control studies.

Beyond atlas studies, we were also curious as to whether issues would arise in datasets that sought to compare samples with different disease statuses or genotypes, even if the dissociation protocol was the same between the different conditions. In the original scRNA-seq paper describing AD/neurodegeneration-associated microglia in the 5xfAD amyloid mouse model[18] the authors noted that one of the datasets they generated was performed using a different dissociation protocol and resulted in the upregulation of a number of stress response and IEGs and they therefore excluded those genes from downstream analysis. While in this case the authors had another dataset to compare, that is often not the case and therefore this subset of data provides an excellent use case for examining the impact of *ex*AM signature when a sub-optimal dissociation protocol is used across all samples.

We performed a reanalysis of this subset of data examined it for the presence of the *ex*AM signature. We found that clusters which were enriched or completely composed of cells from 5xfAD mice showed a decrease in the enrichment of the *ex*AM signature ($p < 1.27 \times 10^{-27}$) and many of the key genes from this signature (**Supplemental Figure 10a-f**). However, as we have shown a number of the key genes in the *ex*AM signature are not expressed at rest in adult microglia (**Supplemental Figure 6**). Therefore, the only way for them to be decreased is for them to be induced in WT cells. The most likely explanation for the differential induction of *exAM* signature is therefore that microglia that are already responding to various stimuli in the 5xfAD mouse brain are less capable of switching activation programs in response to second stressor (i.e., dissociation).

As we demonstrate with our analysis of the microglia subset from Keren-Shaul et al., microglia that are polarized towards a non-resting state (i.e., responsive to amyloid and/or amyloid-associated neurodegeneration) seemingly cannot immediately shift into a new response (i.e., the dissociation response). Therefore, it appears outwardly, that the *ex*AM signature is lower in microglia from AD model mice. Fortunately, Keren-Shaul et al., also had data without this artifact and thus was able to identify it as such and still analyze the true biology at hand. However, this is not normally the case and without knowledge of this transcriptional artifact it could lead to the misidentification of factors important for maintaining microglial homeostasis and their shift towards disease reactive phenotypes. The spurious nature of the observed downregulation of *ex*AM genes in microglia of amyloid mouse models is also confirmed by Ribo-Tag-based analysis of microglia in related AD model[17].

Another case-control example is from Pasciuto et al.,[14] where the authors examined the differences between microglia from WT and major histocompatibility complex II (MHCII) knockout mice by scRNA-seq following enzymatic digestion. We confirm through a reanalysis of the data that the signature identified as downregulated overlaps significantly with our *ex*AM signature (**Supplemental Figure 10g-i**). When we perform gene module scoring using the *ex*AM signature, we find a significant decrease in the signature in cells from MHCII-KO mice ($p < 2.2 \times 10^{-16}$) (**Supplemental Figure 10h**). Furthermore, many of the transcription factors highlighted by the authors as markers of mature microglia *Fos, Egr1, Klf4* have little to no expression at baseline in adult mouse microglia (**Supplemental Figure 6**). Our results regarding low to no expression of many of these markers are also validated by TRAP-Seq/Ribo-Tag sequencing data from microglia in multiple datasets[15–17].

Both of these studies highlight that induction of the *ex*AM signature in control scenario can potentially confound downstream analysis of the true biological signals reflective of the *in vivo* cell states different between groups.

SI Note 5: Comparisons between circulating and tissue-resident immune cells can be confounded by dissociation artifacts.

To examine whether comparisons between blood and tissue resident immune cells were potentially obscured due to dissociation-related signatures we performed reanalysis of two literature datasets in both humans and mice (**Supplemental Figure 12**)[14,19].

In the first analysis of spleen vs. blood natural killer (NK) cells from mice, we found, similar to published analysis, that basic clustering analysis completely separated the cells on the basis of tissue of origin (**Supplemental Figure 12a**). However, when we examined the differential markers between spleen and blood from both the original study and our reanalysis, we noted that they had high overlap with the *ex*AM dissociation signature (**Supplemental Figure 12b-e**). Additionally, gene module scoring on the *ex*AM signature showed enrichment was nearly completely restricted to the dissociated spleen NK cells (**Supplemental Figure 12c**).

Data from Pasciuto et al., 2020 aimed to compare T cells from the brain of human patients to circulating T cells from peripheral blood mononuclear cells (PBMCs) (**Supplemental Figure 12f-l**). As with the mouse NK cells, we found, similar to the published analysis, that basic clustering analysis completely separated the cells on the basis of tissue of origin (**Supplemental Figure 12f**). Further analysis of the genes identified in the original study as different between brain and blood T cells showed that only brain T cells (which had undergone tissue digestion) showed significant upregulation of markers shared with the dissociation signature, including genes that are specifically stress-induced (i.e., *HSPA1AI*) as well as enrichment of the *ex*AM signature via gene module scoring **(Supplemental Figure 12g-k)**.

While we don't have a ground truth for these datasets/cell types it would appear based on the concordance of our *ex*AM signature, dissociation signatures across non-CNS cell types, and the DEGs identified in these studies that the conclusions regarding the transcriptional divergence between tissue-resident and blood immune cells may be significantly biased by differences in dissociation protocols which result in differences in *ex vivo* gene expression.

SI Note 6: Substantial differences across published snRNA-seq literature in gene/transcript sensitivity likely corresponds to improvements/differences in single cell chemistry and nuclei isolation protocols.

During our re-analysis of the literature, we did find that there were significant differences in dataset quality/sensitivity, across all cell types and on cell-type specific level **(Supplemental Figure 15)**. The differences in genes/nucleus and UMIs/nucleus were significantly driven by both improvements in 10X Genomics chemistry across newer versions of their gene expression kits and differences between nuclei isolation protocols. Comparison of our results to that of Morabito et al., 2020[20] found that despite utilizing the same 10X 3' V3 chemistry and control neuropathology tissue with comparable RIN values (**SI Table 2)**, we observed that on average our samples had 39.5% greater median genes per nucleus and 40.9% greater UMIs per nucleus **(Supplemental Figure 15a-b)**. When analyzing just microglial nuclei from each dataset the differences were even starker as on average our median gene per microglia nucleus was nearly double that of Morabito et al., 2020[20] (1813 vs. 925) and median UMI per microglia was nearly 2.5 times greater (3124 vs. 1389) **(Supplemental Figure 15e-f)**. To determine whether donor diagnosis had any effect on gene or UMI detection we also compared across control and AD patients. When comparing either the entire dataset or just microglia we found no differences in between control and AD patients **(Supplemental Figure 15c, g)**. To ensure that the lack of difference was not due to differences in 10X kit chemistry across datasets we also compared internally within the Zhou et al., 2020[21] dataset and found no differences as a result of diagnosis **(Supplemental Figure 15d, h)**.

SI Note 7: Discussion of other potential solutions to avoid *ex vivo* dissociation-induced artifacts in gene expression data & complications with computational solutions.

One emerging alternative approach to isolating cells, is to halt biological processes by keeping tissue/cells cold and using cold-active enzymes, however this often requires difficult and time-consuming optimization, and application to brain tissue is hindered by lack of cold-active and specific DNase[22–24], which is key component of many enzymatic digestion protocols. By contrast, transcriptional/translational inhibitors should be easily applicable to a variety of protocols, simply requiring the addition of the inhibitor cocktail at several steps of the dissociation process.

Implementation of an experimental solution is likely preferable to a computational solution. Some studies have offered a computational approach to exclude or regress genes or components from the clustering steps in the analysis[25–27]. However, components of our identified gene signature are also present in the known cellular stress response to conditions such as cancer, heat stress, hypoxia, and other diseases[28–33]. Thus, it is possible that removing dissociation signatures computationally, could preclude the ability to measure the relevant

contributions of these genes/signatures to true biological signal. Similarly, other studies have opted to remove any cells with high expression of *ex*AM or similar signatures as part of QC before full analysis. However, as we demonstrate in our example re-analysis of two case-control studies **(Supplemental Figure 10; SI Note 4)** the presence of this signature can be modified by experimental factors (e.g. genotype, treatment, etc.) such that removing those cells with high expression of the signature will only serve to further bias downstream analyses.

Another method to reduce *ex vivo* transcriptional changes is to snap-freeze tissue for nuclei isolation and snRNA-seq. While this approach does avoid *ex vivo* activation, it also comes with caveats. Specifically, enrichment of specific cell types via fluorescent activated nuclei sorting (FANS)[34], in unfixed nuclei that is necessary for snRNA-seq, has proven difficult, leading to the need to sequence very large numbers of total nuclei in order to get sufficient numbers of microglia and other rare cell types for properly powered analyses (for review see Liddelow et al., 2020[35]). By contrast, our protocol allows for the use of either unbiased profiling or the use of enrichment/depletion strategies via FACS to increase proportions of rare CNS cell types, though there are caveats to these methods as well depending on surface markers of interest (**SI Note 1)**.

SI Note 8: Discussion of one example where baseline of microglial gene expression has likely been confounded by dissociation induced signatures.

For instance, the presence of the *ex*AM signature in prior studies could lead to misinterpreting the homeostatic/baseline signature of microglia is in adult mice. Among the genes nearly exclusively expressed following enzymatic digestion without inhibitors were transcription factors *Fos* and *Egr1* **(Figure 1d, Figure 2i, Supplemental Figure 2c,d, and Supplemental Figure 6)**. Previous work implied that these transcription factors were an important aspect of the microglial post-natal maturation program and were highly-expressed in homeostatic adult microglia[36–39]. However, as demonstrated both in our own new data presented here, as well as prior scRNA-seq[1], smFISH[40], and TRAP-seq/Ribo-Tag data[15–17], these genes are not highly-expressed in homeostatic/basal conditions but are significantly induced following enzymatic/room temperature digestion.

SI Note 9: Discussion of comparisons between control and Alzheimer's disease patients in regards to confounding microglial and astrocyte signatures in post-mortem data.

In our combined reanalysis we had small numbers of both control donors and those diagnosed with Alzheimer's disease (n=32/17; Ctrl/AD). Given results in other disease states, such as cancer, where a signature similar to the *ex*AM signature is actually reflective of disease[30,33], we wanted to examine whether or not this signature was potentially associated with AD. Our reanalysis revealed no significant differences in these signatures between AD and control patients for either astrocytes or microglia. However, the high overall between-sample heterogeneity that we and others observe with data from human subjects[41,42], highlights the need for scRNA-seq/snRNA-seq analyses in human patients with much greater numbers of samples and therefore such correlations should be revisited in the future with larger integrative analyses. Additionally, as with correlations based on age, it possible that acute pre-mortem and post-mortem factors may be substantially different between disease status and could bias downstream analysis independent of disease-related changes[43].

References

1. Hammond, T. R. *et al.* Single-Cell RNA Sequencing of Microglia throughout the Mouse Lifespan and in the Injured Brain Reveals Complex Cell-State Changes. *Immunity* **50**, 253-271.e6 (2019).
2. Abuzakouk, M., Feighery, C. & O'Farrelly, C. Collagenase and Dispase enzymes disrupt lymphocyte surface molecules. *J Immunol Methods* **194**, 211-216 (1996).
3. Autengruber, A., Gereke, M., Hansen, G., Hennig, C. & Bruder, D. Impact of enzymatic tissue disintegration on the level of surface molecule expression and immune cell function. *Eur J Microbiol Immunol (Bp)* **2**, 112-120 (2012).
4. Botting, R. A. *et al.* Phenotypic and functional consequences of different isolation protocols on skin mononuclear phagocytes. *J Leukoc Biol* **101**, 1393-1403 (2017).
5. Reichard, A. & Asosingh, K. Best Practices for Preparing a Single Cell Suspension from Solid Tissues for Flow Cytometry. *Cytometry A* **95**, 219-226 (2019).
6. Grange, C. *et al.* Phenotypic characterization and functional analysis of human tumor immune infiltration after mechanical and enzymatic disaggregation. *J Immunol Methods* **372**, 119-126 (2011).
7. Schreurs, R. R. C. E. *et al.* Quantitative comparison of human intestinal mononuclear leukocyte isolation techniques for flow cytometric analyses. *J Immunol Methods* **445**, 45-52 (2017).

8. Bendall, S. C. *et al.* Single-cell mass cytometry of differential immune and drug responses across a human hematopoietic continuum. *Science* **332**, 687-696 (2011).
9. Becht, E. *et al.* High-throughput single-cell quantification of hundreds of proteins using conventional flow cytometry and machine learning. *Sci Adv* **7**, eabg0505 (2021).
10. Stoeckius, M. *et al.* Simultaneous epitope and transcriptome measurement in single cells. *Nat Methods* **14**, 865-868 (2017)
11. Mimitou, E. P. *et al.* Scalable, multimodal profiling of chromatin accessibility, gene expression and protein levels in single cells. *Nat Biotechnol* **39,** 1246-1258 (2021).
12. Böttcher, C. *et al.* Human microglia regional heterogeneity and phenotypes determined by multiplexed single-cell mass cytometry. *Nature Neuroscience* **22**, 78-90 (2019).
13. Snijders, G. J. L. J. *et al.* Distinct non-inflammatory signature of microglia in post-mortem brain tissue of patients with major depressive disorder. *Mol Psychiatry* (2020).
14. Pasciuto, E. *et al.* Microglia Require CD4 T Cells to Complete the Fetal-to-Adult Transition. *Cell* **182**, 625-640.e24 (2020).
15. Ayata, P. *et al.* Epigenetic regulation of brain region-specific microglia clearance activity. *Nat Neurosci* **21**, 1049-1060 (2018).
16. Haimon, Z. *et al.* Re-evaluating microglia expression profiles using RiboTag and cell isolation strategies. *Nat Immunol* **19**, 636-644 (2018).
17. Kang, S. S. *et al.* Microglial translational profiling reveals a convergent APOE pathway from aging, amyloid, and tau. *The Journal of Experimental Medicine* **215**, 2235-2245 (2018).
18. Keren-Shaul, H. *et al.* A Unique Microglia Type Associated with Restricting Development of Alzheimer's Disease. *Cell* **169**, 1276-1290.e17 (2017).
19. Crinier, A. *et al.* High-Dimensional Single-Cell Analysis Identifies Organ-Specific Signatures and Conserved NK Cell Subsets in Humans and Mice. *Immunity* **49**, 971-986.e5 (2018).
20. Morabito, S., Miyoshi, E., Michael, N. & Swarup, V. Integrative genomics approach identifies conserved transcriptomic networks in Alzheimer's disease. *Hum Mol Genet* (2020).
21. Zhou, Y. *et al.* Human and mouse single-nucleus transcriptomics reveal TREM2-dependent and TREM2-independent cellular responses in Alzheimer's disease. *Nature Medicine* **26**, 131-142 (2020).
22. Anisimova, V. E. *et al.* Isolation, characterization and molecular cloning of duplex-specific nuclease from the hepatopancreas of the Kamchatka crab. *BMC Biochem* **9**, 14 (2008).
23. Anisimova, V. E., Barsova, E. V., Bogdanova, E. A., Lukyanov, S. A. & Shcheglov, A. S. Thermolabile duplex-specific nuclease. *Biotechnol Lett* **31**, 251-257 (2009).
24. Nilsen, I. W. *et al.* The enzyme and the cDNA sequence of a thermolabile and double-strand specific DNase from Northern shrimps (Pandalus borealis). *PLoS One* **5**, e10295 (2010).
25. Saunders, A. *et al.* Molecular Diversity and Specializations among the Cells of the Adult Mouse Brain. *Cell* **174**, 1015-1030.e16 (2018).
26. Sankowski, R. *et al.* Mapping microglia states in the human brain through the integration of high-dimensional techniques. *Nat Neurosci* **22**, 2098-2110 (2019).
27. Pombo Antunes, A. R. *et al.* Single-cell profiling of myeloid cells in glioblastoma across species and disease stage reveals macrophage competition and specialization. *Nat Neuroscience 24, 595-610* (2021).
28. Majmundar, A. J., Wong, W. J. & Simon, M. C. Hypoxia-inducible factors and the response to hypoxic stress. *Mol Cell* **40**, 294-309 (2010).
29. Hashimoto-Torii, K. *et al.* Roles of heat shock factor 1 in neuronal response to fetal environmental risks and its relevance to brain disorders. *Neuron* **82**, 560-572 (2014).
30. Tirosh, I. *et al.* Dissecting the multicellular ecosystem of metastatic melanoma by single-cell RNA-seq. *Science* **352**, 189-196 (2016).
31. Gomez-Pastor, R., Burchfiel, E. T. & Thiele, D. J. Regulation of heat shock transcription factors and their roles in physiology and disease. *Nat Rev Mol Cell Biol* **19**, 4-19 (2018).
32. Vihervaara, A., Duarte, F. M. & Lis, J. T. Molecular mechanisms driving transcriptional stress responses. *Nat Rev Genet* **19**, 385-397 (2018).
33. Baron, M. *et al.* The Stress-Like Cancer Cell State Is a Consistent Component of Tumorigenesis. *Cell Syst* (2020).
34. Nott, A. *et al.* Brain cell type-specific enhancer-promoter interactome maps and disease-risk association. *Science* **366**, 1134-1139 (2019).
35. Liddelow, S. A., Marsh, S. E. & Stevens, B. Microglia and Astrocytes in Disease: Dynamic Duo or Partners in Crime. *Trends Immunol* **41**, 820-835 (2020).
36. Butovsky, O. *et al.* Identification of a unique TGF-β-dependent molecular and functional signature in microglia. *Nat Neurosci* **17**, 131-143 (2014).

37. Matcovitch-Natan, O. *et al.* Microglia development follows a stepwise program to regulate brain homeostasis. *Science* **353**, aad8670 (2016).
38. Butovsky, O. & Weiner, H. L. Microglial signatures and their role in health and disease. *Nat Rev Neurosci* (2018).
39. Krasemann, S. *et al.* The TREM2-APOE Pathway Drives the Transcriptional Phenotype of Dysfunctional Microglia in Neurodegenerative Diseases. *Immunity* **47**, 566-581.e9 (2017).
40. Li, Q. *et al.* Developmental Heterogeneity of Microglia and Brain Myeloid Cells Revealed by Deep Single-Cell RNA Sequencing. *Neuron* **101**, 207-223.e10 (2019).
41. Welch, J. D. *et al.* Single-Cell Multi-omic Integration Compares and Contrasts Features of Brain Cell Identity. *Cell* **177**, 1873-1887.e17 (2019).
42. de Paiva Lopes, K. *et al.* Genetic analysis of the human microglial transcriptome across brain regions, aging and disease pathologies. *Nat Genet* **54**, 4-17 (2022).
43. Miller, J. A. *et al.* Neuropathological and transcriptomic characteristics of the aged brain. *Elife* **6**, e31126 (2017).
