# Supplementary material for: Dissection of artifactual and confounding glial signatures by single-cell sequencing of mouse and human brain
Source: Nat Neurosci. 2022 Mar 8;25(3):306–16. doi: 10.1038/s41593-022-01022-8 (PMC11645269; doi:10.1038/s41593-022-01022-8)
Supplement: Supplementary file 2 — Reporting Summary [file 41593_2022_1022_MOESM2_ESM.pdf]

nature research

Corresponding author(s): Beth Stevens

Last updated by author(s): 1/18/2022

Reporting Summary

Nature Research wishes to improve the reproducibility of the work that we publish. This form provides structure for consistency and transparency in reporting. For further information on Nature Research policies, see our Editorial Policies and the Editorial Policy Checklist.

Statistics

For all statistical analyses, confirm that the following items are present in the figure legend, table legend, main text, or Methods section.

n/a	Confirmed	
☐	☒	The exact sample size (*n*) for each experimental group/condition, given as a discrete number and unit of measurement
☐	☒	A statement on whether measurements were taken from distinct samples or whether the same sample was measured repeatedly
☐	☒	The statistical test(s) used AND whether they are one- or two-sided<br>*Only common tests should be described solely by name; describe more complex techniques in the Methods section.*
☐	☒	A description of all covariates tested
☐	☒	A description of any assumptions or corrections, such as tests of normality and adjustment for multiple comparisons
☐	☒	A full description of the statistical parameters including central tendency (e.g. means) or other basic estimates (e.g. regression coefficient) AND variation (e.g. standard deviation) or associated estimates of uncertainty (e.g. confidence intervals)
☐	☒	For null hypothesis testing, the test statistic (e.g. *F*, *t*, *r*) with confidence intervals, effect sizes, degrees of freedom and *P* value noted<br>*Give P values as exact values whenever suitable.*
☒	☐	For Bayesian analysis, information on the choice of priors and Markov chain Monte Carlo settings
☒	☐	For hierarchical and complex designs, identification of the appropriate level for tests and full reporting of outcomes
☒	☐	Estimates of effect sizes (e.g. Cohen's *d*, Pearson's *r*), indicating how they were calculated

Our web collection on statistics for biologists contains articles on many of the points above.

Software and code

Policy information about availability of computer code

Data collection	Description is stated in methods and here:<br>Raw sequencing data was processed as stated in methods and NCBI GEO/EGA/dbGAP records. 10X Genomics Cell Ranger software (v2.2, 3.0, 3.1, or 5.0 as specified in SI Tables 1-2) and bcl2fastq (v2.20.0.422)<br>Mouse single cell analysis was performed using R (v3.4.3, v3.5.1, and 3.6.1) and primarily using the single cell analysis package Seurat (v2.3.4, v3.1.5, and 3.2.3). Additional analysis of human single nuclei samples was performed using LIGER development branch “online”. Other supplemental R packages were used as described in methods below. To determine if the proportions of cells per cluster were significantly different between the different protocols the clusters were compared using the R package speckle v0.0.1 (https://github.com/Oshlack/speckle). Additional single cell analysis and plotting was performed using scCustomize v0.5.0.<br>FACS sorting was performed using FACSDiva v8.0.1.<br>GraphPad Prism v8.4.3 was used for other statistics and plotting as described in methods.
Data analysis	Link to code used to generate single cell objects used in analysis can be found in methods section of manuscript and here: https://github.com/samuel-marsh/Marsh_et-al_2022_scRNAseq_Dissociation_Artifacts

For manuscripts utilizing custom algorithms or software that are central to the research but not yet described in published literature, software must be made available to editors and reviewers. We strongly encourage code deposition in a community repository (e.g. GitHub). See the Nature Research guidelines for submitting code & software for further information.

Data

Policy information about availability of data

All manuscripts must include a data availability statement. This statement should provide the following information, where applicable:

- Accession codes, unique identifiers, or web links for publicly available datasets
- A list of figures that have associated raw data
- A description of any restrictions on data availability

Data & Software Availability: Raw sequencing data for all mouse samples was deposited in the NCBI GEO database under the SuperSeries GSE152184 which contains the following subseries: GSE152183 (Mouse Microglia 4 dissociation protocols), GSE152182 (Mouse All CNS Cells), GSE152210 (Mouse Microglia PBS Tail Vein), GSE188441 (Mouse 10X Version Comparison). Cell Ranger output files are available as supplementary files via GEO and raw fastq files can be accessed from SRA linked from GEO records. Raw sequencing data for post-mortem human tissue was deposited in NCBI GEO database under the super series GSE152184 in the subseries: GSE157760. Cell Ranger output files are available as supplementary files via GEO and raw fastq files can be accessed from SRA linked from GEO records. Raw sequencing data for the acutely isolated human tissue was deposited in European phenome-Genome Archive (EGA) (Accession ID: in progress,).. Raw sequencing data and processed count matrix files for human PBMC dataset are deposited in dbGAP (Accession #: phs002222.v2.p1).

Field-specific reporting

Please select the one below that is the best fit for your research. If you are not sure, read the appropriate sections before making your selection.

☒ Life sciences ☐ Behavioural & social sciences ☐ Ecological, evolutionary & environmental sciences

For a reference copy of the document with all sections, see nature.com/documents/nr-reporting-summary-flat.pdf

Life sciences study design

All studies must disclose on these points even when the disclosure is negative.

Sample size	Sample sizes were chosen to allow for the comparisons between multiple replicates in all of the groups, with the exception of pilot study in human PBMCs. Sample sizes and pilot nature of experiment are clearly stated in mnauscript, Figure Panel A, and Figure Legend.
Data exclusions	No data was discarded except for data filtered during dataset quality control (applies to all single-cell/single-nuclei experiments), which was performed according to field standard techniques as described in methods and exact code provided in linked GitHub repo. Deposited data is both raw sequencing reads fastq (no exclusions) and Cell Ranger software outputs (no exclusions).
Replication	Replication was confirmed using multiple biological replicates in all experiments and through reanalysis of literature datasets which confirm the effects observed in currently generated datasets. smFISH experiments were performed twice on independent biological replicates. Human post-portem single nuclei data contains data from two libraries per donor processed in different batches/experiments with identical protocols before sequencing samples at the same time.
Randomization	Only experiment that involved required multiple batches was the mouse microglia experiment (Figure 1, SI Figures 1-3, 6). Samples in this experiment were randomized so that samples from each experimental group was present in both batches and order was staggered. The ordering and batching of samples is provided in SI Table 1. For human experiments with case/control design (freezing delay and mock digestion) all collected samples were split evenly between case-control groups. There is no experimental group assignment for post-mortem experiment. Case/control status for reanalysis of public datasets was performed using the annotations/meta data provided in those publications.
Blinding	For mouse experiments: Investigators were not blind to experimental condition during tissue collection due to the conditions of the experiment. FACS sorting of cells and generation of single cell libraries was performed blind. scRNA-seq analyses were not performed blind but using automated and appropriate and field standard analysis techniques that require meta data to be present. All statistical measures (DEG, cell/nuclei abundance, etc) was performed using were performed using automated pipelines (DESeq2, speckle, MAST).<br><br>For Human experiments: Acute tissue collection/freezing delay and mock digestion was not performed blind due to the nature of the experiment. Nuclei isolation, FACS sort of nuclei, and library generation was all performed blind. snRNA-seq/scRNA-seq analyses were not performed blind but using automated and appropriate and field standard analysis techniques that require meta data to be present.<br>Initial scRNA-seq/snRNA-seq experiments were performed relatively blind but proper integration and analysis of diverse datasets requires periodic unblinding to assess analysis. All statistical measures (DEG, cell/nuclei abundance, etc) was performed using were performed using automated pipelines (DESeq2, speckle, MAST).

Reporting for specific materials, systems and methods

We require information from authors about some types of materials, experimental systems and methods used in many studies. Here, indicate whether each material, system or method listed is relevant to your study. If you are not sure if a list item applies to your research, read the appropriate section before selecting a response.

Materials & experimental systems

n/a	Involved in the study
☐	☒ Antibodies
☒	☐ Eukaryotic cell lines
☒	☐ Palaeontology and archaeology
☐	☒ Animals and other organisms
☐	☒ Human research participants
☒	☐ Clinical data
☒	☐ Dual use research of concern

Methods

n/a	Involved in the study
☒	☐ ChIP-seq
☐	☒ Flow cytometry
☒	☐ MRI-based neuroimaging

Antibodies

Antibodies used	Anti-CD16/CD32 to block Fc receptors (1:50; BioLegend Cat #: 553141).<br>The following antibodies were then added to cell suspensions at 1:200 final concentration:<br>CD11b-BV421 (BioLegend Cat#: 101236 Clone: M1/70)<br>CD45-PE (BioLegend Cat#: 103106 Clone: 30-F11)<br>CX3CR1-Alexa Fluor 647 (BioLegend Cat#: 149004: Clone: SA011F11s).
Validation	Validation as reported by supplier (BioLegend):<br>Anti-CD16/CD32 (1:50; BioLegend Cat #: 553141). to block Fc receptors is quality control tested for flow cytometry and well-validated/reported in literature to block CD16 & CD32 (Fc Receptors) (See BioLgend Citation library on product page).<br>CD11b-BV421 (BioLegend Cat#: 101236 Clone: M1/70) is quality control tested for flow cytometry.<br>CD45-PE (BioLegend Cat#: 103106 Clone: 30-F11) is quality control tested for flow cytometry and shown to bind to both CD45.1 and CD45.2 isoforms.<br>CX3CR1-Alexa Fluor 647 (BioLegend Cat#: 149004: Clone: SA011F11s)) is quality control tested for flow cytometry.

Animals and other organisms

Policy information about studies involving animals; ARRIVE guidelines recommended for reporting animal research

Laboratory animals	As stated in methods section of the paper and copied here:<br>All mouse studies used C57BL/6J (Stock # 000664) purchased from Jackson Laboratories. Mouse single cell sequencing studies were performed with male mice at P89-90. smFISH studies were performed with mice ranging from P90-P120. Both male and female mice were used for smFISH studies. All animals were group housed on a 12-h/12-h light/dark cycle with access to food and water ad libitum. All experiments were reviewed and overseen by the institutional animal use and care committee at Boston Children's Hospital in accordance with all NIH guidelines for the humane treatment of animals. For full breakdown of replicates per dataset for mouse experiments see SI Table 1.
Wild animals	Study did not involve the use of wild animals.
Field-collected samples	Study did not involved field collected animals.
Ethics oversight	All experiments were reviewed and overseen by the institutional animal use and care committee at Boston Children's Hospital in accordance with all NIH guidelines for the humane treatment of animals.

Note that full information on the approval of the study protocol must also be provided in the manuscript.

Human research participants

Policy information about studies involving human research participants

Population characteristics	All demographic information was provided in SI Table 2.
Recruitment	Only recruitment was for acute human tissue. Acutely isolated human brain tissue was obtained with informed consent under protocol REC 16/LO/2168 approved by the NHS Health Research Authority.
Ethics oversight	As also stated in methods section and repeated here:<br>Post-Mortem Human Brain:<br>Post-mortem autopsy tissue from control cases was obtained from the Massachusetts Alzheimer's Disease Research Center (MADRC) at Massachusetts General Hospital (MGH). Tissue were collected with informed consent of patients or their relatives and approval of Massachusetts General Hospital Institutional Review Board. Human patient demographic information for tissue in the current study is provided in Table S2. Post-mortem tissue processing and sequencing experiments were performed at the Broad Institute and approved by Broad Institute's Office of Research Subject Protection. Recruitment was performed by MGH brain bank/MADRC and these were samples supplied as neuropathological control samples.<br>Acute Human Tissue:<br>Acutely isolated human brain tissue was obtained with informed consent under protocol REC 16/LO/2168 approved by the NHS Health Research Authority. Samples were transferred subject to MTA agreements between institutions and use and

processing of acute brain tissue and sequencing were reviewed and approved by Boston Children's Hospital Institutional Review Board and Broad Institute's Office of Human Research Subjects Protection. Samples came from GBM patients and biased by those recruitment characteristics.
Human PBMCs:
Human PBMCs were obtained from blood drawn from a healthy volunteer (male, 35 y.o.) in lithium heparin coated tubes. Blood was collected in accordance with a protocol approved by Yale University Institutional Review Board with the informed consent of the patients.

Note that full information on the approval of the study protocol must also be provided in the manuscript.

Flow Cytometry

Plots

Confirm that:

☒ The axis labels state the marker and fluorochrome used (e.g. CD4-FITC).

☒ The axis scales are clearly visible. Include numbers along axes only for bottom left plot of group (a 'group' is an analysis of identical markers).

☒ All plots are contour plots with outliers or pseudocolor plots.

☒ A numerical value for number of cells or percentage (with statistics) is provided.

Methodology

Sample preparation	As stated in methods (copied and pasted directly below):<br>All steps were performed on ice or using pre-chilled refrigerated centrifuge set to 4C with all buffers/solutions prechilled before addition to samples. Cell suspensions (50µl) were incubated for 20 minutes on ice with anti-CD16/CD32 to block Fc receptors (1:50; BioLegend Cat #: 553141) and with a viability dye; eFlour780 (1:1000; Thermo Fisher Scientific Cat# 50-169-66) to identify live cells. Antibody master mix was created by adding all antibodies at 2X their final concentration, 10µl of Brilliant Stain Buffer Plus (BD Biosciences Cat#: 566385), to FACS buffer. The master mix was composed of the following antibodies: CD11b-BV421 (BioLegend Cat#: 101236; Clone: M1/70; 1:100 master mix; final staining concentration 1:50), CD45-PE (BioLegend Cat#: 103106; Clone: 30-F11; 1:200 master mix; final staining concentration 1:100), CX3CR1-Alexa Fluor 647 (BioLegend Cat#: 149004; Clone: SA011F11; 1:500 master mix; final staining concentration 1:250). Following Fc block, 50µl of antibody master mix was added to each sample to achieve 1X antibody concentration. Samples were incubated with staining antibodies for 20 min at 4C and then spun down for 5 min at 300g, before being resuspended in 500µl of FACS buffer.
Instrument	BD FACSAria-SORP (Special Order system).
Software	BD FACS Diva and FlowJo (v10)
Cell population abundance	See SI Figure 1.
Gating strategy	Gating strategy for myeloid cell sort was as follows (SI Figure1b: Live (live/dead eFluor780), cells vs. debris (FSC-A vs. SSC-A), singlets (FSC-H vs. FSC-A), CD45+/CD11b+. We intentionally set a liberal gate of any CD11b+/CD45+ cells so as to not bias our scRNA-seq due to changes in cell surface receptor expression (SI Figure 1b-c). CX3CR1 was excluded as parameter used for cell sort due to differences in staining between isolation methods as result of cleavage of extracellular epitopes by enzymes (SI Figure 1d-e).

☒ Tick this box to confirm that a figure exemplifying the gating strategy is provided in the Supplementary Information.
